# Microwave-assisted synthesis, molecular docking studies of 1,2,3-triazole-based carbazole derivatives as antimicrobial, antioxidant and anticancer agents†

**DOI:** 10.1039/d2ra05960f

**Published:** 2022-12-19

**Authors:** Dongamanti Ashok, Gugulothu Thara, Bhukya Kiran Kumar, Gundu Srinivas, Dharavath Ravinder, Thumma Vishnu, Madderla Sarasija, Bujji Sushmitha

**Affiliations:** Green and Medicinal Chemistry Laboratory, Department of Chemistry, Osmania University Hyderabad-500007 Telangana India ashokdou@gmail.com; Department of Pharmacy, University College of Technology, Osmania University Hyderabad-500007 Telangana India; Department of Microbiology, University College of Science, Osmania University Hyderabad-500007 Telangana India; Deparment of Sciences and Humanities, Matrusri Engineering College Hyderabad-500059 Telangana India; Department of Chemistry, Satavahana University Karimnagar-505001 Telangana India

## Abstract

Herein, a new series of *N*-substituted 1,2,3-triazolylmethyl indole derivatives 4(a–u) was synthesized by rationally incorporating a pharmacophoric active heterocyclic ring containing indole and triazole moieties in one molecular frame *via* the conventional and microwave irradiation methods. Briefly, the new compounds 4(a–u) were synthesized *via* the *N*-alkylation of tetrahydro-1*H*-carbazoles followed by click reaction and copper-catalyzed Huisgen [3 + 2] cycloaddition in the presence of copper sulphate and sodium ascorbate with various aromatic azides 3(a–m). All the newly synthesized compounds were characterized *via*^1^H and ^13^C NMR, mass, and IR spectroscopy and evaluated for their antimicrobial, antioxidant and anticancer activities. Among the synthesized compounds, 4d, 4j, 4n, 4p, 4s and 4r were found to exhibit good antimicrobial, antioxidant, anticancer activities. The biological activity of the synthesized compounds was further supplemented by molecular docking studies against the target receptors caspase-3 and 17-beta-hydroxy steroid dehydrogenase type 1, revealing that the reported structures best fit into the active site pocket of the target molecules.

## Abstract 2

Easy and approachable synthesis. Conventional and microwave irradiational methods. 21 examples. *In vitro* antimicrobial, antioxidant and anticancer activities. Molecular docking studies.

## Graphical abstract

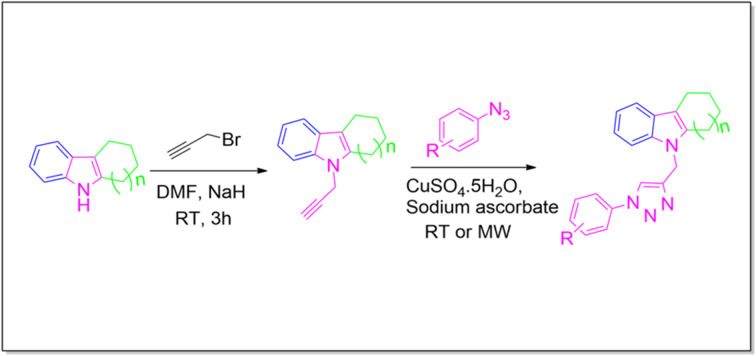

## Introduction

**Fig. 1:**
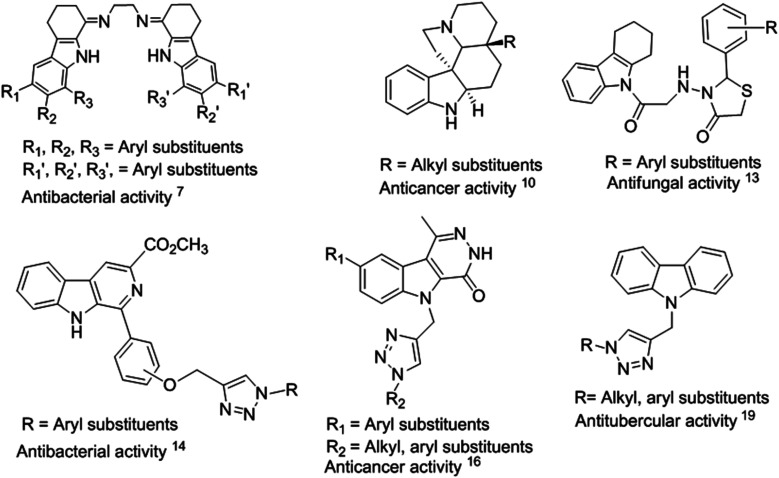
Biologically active naturally occurring and synthetic indole derivatives.

Indole is an aromatic heterocyclic organic compound, which is found in many natural products from animals and marine organisms.^1^ The core moiety of indole is present in various natural products of plants such as jasmine, citrus fruits, orange blossoms, and *Robinia pseudoacacia*. Indole and its derivatives play a significant role in biological activities.^2^ Thus, researchers have been inspired to investigate the pharmacological applications of indole scaffolds for therapeutic applications.^1^ Tetrahydrocarbazoles are a sub-class of indoles and an important part of many naturally occurring alkaloids, such as ondansetron,^2^ ervatamine,^3^ reserpine^4^ and etodolac.^5^ Tetrahydrocarbazoles exhibit diverse biological activities, such as antibacterial,^6,7^ antitubercular, antioxidant,^8^ anticancer,^9,10^ antidepressant,^11^ antivascular,^12^ and antifungal^13^ (Fig. 1).

Some of the known indole-containing triazole-based moieties have been found to exhibit wide variety of biological activities such as antibacterial,^14^ antioxidant,^15^ anticancer,^16–18^ and antitubercular^19^ (Fig. 1). Among them, tetrahydrocarbazoles containing a 1,2,3-triazole moiety are emerging for drug discovery with multifunctional activities.^20^ This is because 1,2,3-triazoles are potent anticancer agents and act as inhibitors for caspase-3 (ref. 21–23) and 17β-hydroxysteroid dehydrogenase.^24^

The main cause of death of living organisms is the growing drug resistance of microbes and increase in the number of microbial diseases.^14^ Therefore, it is necessary to find new alternative scaffolds that have potential as lead molecules to fight against multidrug resistance bacterial infections.^25^ In recent years, the microwave irradiation (MWI) method has become popular in synthetic reaction processes to improve the reaction yield, while avoiding harsh conditions.^26,27^ Furthermore, the MWI reaction technique is convenient, environmentally friendly, clean, and economical. In the MW irradiation method, two or more reactants in one reaction vessel give a new product within a short period, in high yield and with a lower amount of by-products.^28^ The products can be purified easily, and occasionally the selectivity can be changed. In fact, microwaves can be used to execute new reactions and circumstances that are not possible with ordinary heating.

A new series of 1,2,3-triazole-based molecules was designed and synthesized in our laboratory,^29^ which exhibited promising antimicrobial activity,^30,31^ and some of which are the subject of detailed pharmacological investigations. Our designed scaffold mainly originated from a multifunctional unit containing a bioactive molecule (tryptamine core).^20^ The designed scaffold contains three pharmacophoric parts including a multifunctional indole nucleus, triazole unit, and aromatic and aliphatic units to increase the additional pharmacophoric and biological activity of the title compounds. In our continuing efforts to develop new antimicrobial, antioxidant and anticancer agents, followed by molecular docking studies, in the present work, we designed and synthesized a series of novel *N*-substituted 1,2,3-triazolylmethyl indole derivatives 4(a–u) employing the conventional and microwave irradiation methods *via* copper-catalyzed Huisgen [3 + 2] cycloaddition reaction. All the synthesized compounds were tested for their antimicrobial, antioxidant, and anticancer activities, followed by molecular docking studies.

## Result and discussion

### Chemistry

**Scheme 1:**
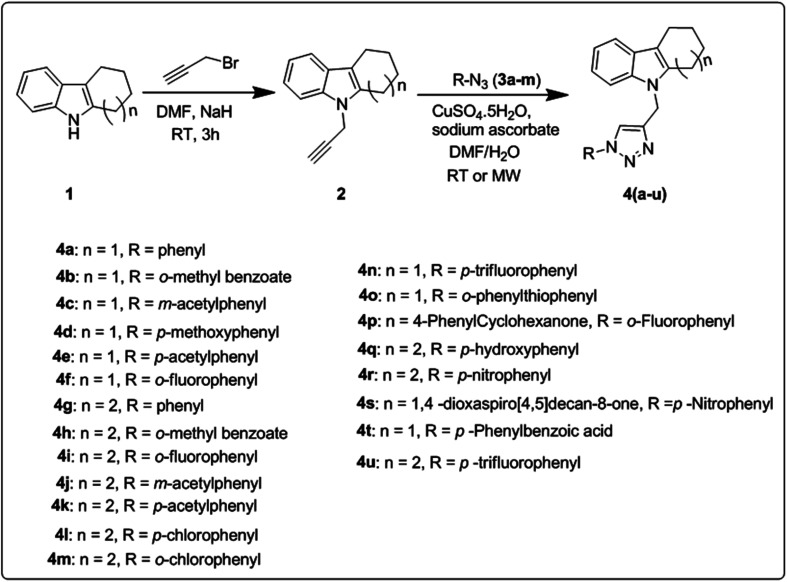
Synthetic route for novel *N*-substituted 1,2,3-triazolylmethyl indole derivatives 4(a–u).

The synthetic route for the preparation of a series of novel *N*-substituted 1,2,3-triazolylmethyl indole derivatives is shown in Scheme 1. Initially, substituted cyclohexanones were reacted with phenyl hydrazine to furnish different tetrahydrocarbazoles (1) according to a literature procedure.^32^ Further *N*-alkylation of the corresponding tetrahydrocarbazole (1) with propargyl bromide in the presence of NaH (sodium hydride as a base) in DMF (dimethylformamide) solvent gave 9-(prop-2-yn-1-yl)-2,3,4,9-tetrahydro-1*H*-carbazole (2) in good yield following a literature protocol.^33,34^ Subsequently, click reaction of the resulting alkynes (2) through copper-catalyzed Huisgen [3 + 2] cycloaddition reaction using CuSO_4_ (copper sulphate) and sodium ascorbate with different aromatic azides 3(a–m) gave *N*-substituted 1,2,3-triazolyl methyl indole derivatives 4(a–u).

To improve the yield and reaction time, optimization of the conditions for the copper-catalyzed Huisgen [3 + 2] cycloaddition reaction was carried out using different catalysts and solvents employing the conventional and microwave irradiation methods, as tabulated in Table 1. According to the preliminary screening, among the tested conditions, CuSO_4_ and sodium ascorbate catalyst and a 2 : 1 ratio of DMF/H_2_O as the solvent were found to be the optimum conditions for both the conventional and MWI (microwave irradiation) methods. Between these two methods, we found that the microwave irradiation method gave better yields (72–96%) together with a shorter reaction time compared to the conventional method (64–94%). The reaction time and yields of both methods are presented in Table 2.

**Table 1:** Optimization of reaction conditions for compounds 4(a–u)

Entry	Catalyst	Solvent	Conventional method	Microwave irradiation method
			(Yield%)	(Yield%)
1	N-Heterocyclic carbene catalyst	Solvent- free	10	18
2	CuI	Glycerol	17	25
3	CuI, NEt_3_	DMSO	38	46
4	CuI, sodium ascorbate	MeCN/H_2_O (2 : 1)	58	67
5	CuSO_4_, sodium ascorbate	*t*-BuOH/H_2_O (1 : 1)	75	80
6	CuSO_4_, sodium ascorbate	THF/H_2_O (1 : 1)	86	88
7	CuSO_4_, sodium ascorbate	DMF/H_2_O (2 : 1)	82	95

**Table 2:** Physicochemical properties of synthesized compounds 4(a–u)

Compound	M.P. (°C)	Conventional		MWI	
		Time, h	(Yield%)	Time, min	(Yield%)
4a	112–114	8	81	5	95
4b	100–102	8	76	5	90
4c	116–118	8	70	4	91
4d	155–157	8	80	5	83
4e	182–184	8	76	4	87
4f	114–116	8	75	4	90
4g	146–148	10	74	5	85
4h	126–128	10	79	6	85
4i	120–122	10	66	6	72
4j	173–175	10	64	5	76
4k	138–140	10	71	5	92
4l	168–170	10	63	5	90
4m	150–152	10	75	6	96
4n	104–106	8	82	5	92
4o	131–133	9	78	4	94
4p	145–147	9	68	4	90
4q	160–162	8	80	6	85
4r	147–149	8	71	6	87
4s	153–155	10	68	4	95
4t	121–123	10	77	4	90
4u	152–154	10	70	6	72

The obtained alkynes 4(a–u) were fully characterized *via*^1^H and ^13^C NMR, IR and mass spectrometry. The ^1^H NMR spectrum of compound 4a showed the N–CH_2_ proton peak at *δ* 5.44 as a singlet (2H), while in the ^13^C NMR spectrum, the N–CH_2_ carbon appeared at *δ* 38.58. The mass spectrum of compound 4a displays the molecular ion peak at *m*/*z* 329 [M + H]^+^ (100). All the other spectral data are shown in the Experimental section.

### Biological activity

All the title compounds 4(a–u) were screened for their antimicrobial, antioxidant and anticancer activities. The *in vitro* antibacterial, antifungal, antioxidant and anticancer activities of the synthesized compounds are tabulated in Tables 3, 4 and 5, respectively.

**Table 3:** Antimicrobial activities of synthesized compounds 4(a–u) (zone of inhibition in mm)^a^

Gram positive bacteria					Gram negative bacteria						Fungal strain	
Entry	*B. subtilis*		*S. aureus*		*E. coli*		*P. aeruginosa*		*K. pneumoniae*		*Aspergillus flavus*	
	10 μM	20 μM	10 μM	20 μM	10 μM	20 μM	10 μM	20 μM	10 μM	20 μM	10 μM	20 μM
4a	5.13	12.04	3.90	9.52	6.03	11.02	8.15	12.04	10.50	16.10	6.51	11.03
4b	6.53	12.563	7.51	13.10	6.06	12.47	8.59	14.24	10.38	17.40	5.55	11.43
4c	9.60	14.08	7.97	15.24	8.43	15.64	7.50	14.82	11.02	15.59	5.63	12.50
4d	11.22	21.02	12.3	15.2	10.13	22.46	7.82	12.32	10.92	19.92	10.25	20.14
4e	10.71	14.58	12.54	17.92	11.72	16.45	11.64	17.88	7.53	12.32	7.58	15.90
4f	8.80	15.46	9.70	16.78	7.74	16.87	13.00	18.69	7.83	13.76	8.32	14.91
4g	8.82	16.90	12.57	16.62	7.69	15.49	8.59	17.43	11.78	16.80	9.51	12.60
4h	10.50	15.10	9.76	17.73	8.81	16.51	12.20	18.73	7.48	16.80	8.74	15.90
4i	9.89	13.91	12.55	17.88	8.53	17.97	12.55	16.92	13.30	16.84	13.72	17.74
4j	10.75	22.62	12.45	21.73	11.34	18.97	11.82	19.86	8.99	17.15	12.71	21.52
4k	10.82	17.89	14.73	18.62	9.86	15.46	10.63	18.21	9.91	14.92	9.93	16.90
4l	9.90	15.95	13.52	18.95	13.42	16.71	10.08	16.99	13.80	18.81	12.87	17.81
4m	12.32	16.72	10.10	18.80	8.98	16.98	13.56	17.50	13.52	18.80	9.70	15.60
4n	12.24	21.38	13.50	24.40	12.58	17.71	10.53	20.85	12.73	22.80	10.97	19.82
4o	13.80	18.42	8.80	16.46	12.62	17.90	9.81	17.73	10.92	18.74	9.91	15.70
4p	12.65	23.90	11.62	21.94	12.13	21.92	9.64	17.75	11.92	20.60	12.75	18.92
4q	11.51	15.60	10.25	14.59	12.41	16.11	11.23	17.46	10.51	15.89	8.93	13.46
4r	10.44	15.16	9.46	16.44	12.13	14.56	10.32	14.56	10.77	15.13	9.16	12.03
4s	10.47	20.23	9.25	15.13	12.34	23.14	11.35	19.12	11.87	20.26	8.23	10.23
4t	9.16	14.32	11.25	17.16	12.34	16.46	10.23	14.38	10.48	14.25	9.12	13.46
4u	10.24	12.34	11.25	14.23	11.99	14.54	12.78	15.67	10.48	13.46	8.12	12.46
Am	13.90	25.60	12.74	22.91	12.54	23.72	13.30	24.93	13.84	25.91	—	—
Ny	—	—	—	—	—	—	—	—	—	—	13.94	25.80

a Am = ampicillin, Ny = nystatin.

### Antimicrobial activity

All the synthesized compounds 4(a–u) were evaluated for their antibacterial activity against two Gram-positive strains, *i.e.*, *Bacillus subtilis* (MTCC 121) and *Staphylococcus aureus* (MTCC96), and three Gram-negative bacterial strains, *i.e.*, *Pseudomonas aeruginosa* (ATCC-27853), *Escherichia coli* (MTCC43), and *Klebsiella pneumonia* (MTCC 530), at a concentration of 10 μM and 20 μM using the well diffusion method.^35^*Ampicillin* was used as the standard antibiotic, the zone of inhibition around the well was measured in mm and the results are demonstrated in Table 3. Among the synthesized compounds 4d, 4j, 4n, 4p, and 4s were found to be the most potent due to the presence of electron-withdrawing groups on the triazole ring. Compounds 4h, 4o, 4k, and 4e exhibited good antibacterial activity against selected all the bacterial strains compared to that of the standard drug. In conclusion, 4n, 4p, and 4s exhibited excellent antibacterial activity against the tested bacterial strains.

In addition, all the synthesized indole derivatives (4a–u) were evaluated for their antifungal activity against one fungal strain, *Aspergillus flavus* (NRRL-3357), at a concentration of 10 μM and 20 μM using the well diffusion method,^35^ and the zone of inhibition was measured in mm compared with the standard drug *Nystatin*. Among the synthesized compounds 4d (Ar = *p*-OCH_3_), 4j (Ar = *m*-COCH_3_), 4n (Ar = *o*-S-Ph) and 4p (Ar = *o*-F) showed high activity against *Aspergillus flavus* and the results are depicted in Table 3.

### Antioxidant activity

The antioxidant activity of all the synthesized compounds 4(a–u) was investigated using the DPPH (2,2-diphenyl-1-picryl-hydrazyl-hydrate)^36^ and HRS (hydroxyl radical scavenging)^37^ methods at different concentrations (25 μM and 50 μM) with ascorbic acid as the standard drug. Based on the antioxidant activity of all the tested compounds, 4e, 4f, 4p, and 4q exhibited highly potent free radical scavenging activity in the DPPH and HRS methods. Among them, compounds 4e (Ar = *p*-COMe) and 4f (Ar = *o*-F) with electron-withdrawing groups in the triazole ring exhibited the best antioxidant activity. Alternatively, compounds 4b, 4i, 4m, 4n, 4t, and 4u showed equipotent activity against *Ascorbic acid* as a standard drug. The radical scavenging activity results are shown in Table 4.

**Table 4:** Radical scavenging activities of the synthesized compounds 4(a–u)

Concentration (μM)				
Entry	DPPH method (%)		HRS method (%)	
	25 μM	50 μM	25 μM	50 μM
4a	27.28	47.15	21.13	46.14
4b	28.32	53.18	24.16	52.23
4c	23.16	41.23	18.23	38.16
4d	28.16	40.16	23.16	41.26
4e	31.58	57.44	25.12	52.16
4f	33.16	67.19	24.64	53.17
4g	25.46	38.46	17.56	41.03
4h	23.66	43.26	18.16	47.46
4i	27.46	51.23	26.23	52.46
4j	28.11	49.46	23.46	37.16
4k	18.46	38.49	24.16	49.16
4l	19.49	40.16	22.19	41.26
4m	24.16	56.16	26.56	51.23
4n	23.50	52.23	24.18	52.16
40	21.26	42.19	19.46	45.16
4p	32.18	58.50	27.49	53.20
4q	31.32	65.20	24.48	50.18
4r	29.66	39.47	18.45	43.18
4s	26.49	42.18	18.45	48.46
4t	26.44	51.43	26.24	56.46
4u	27.16	54.16	23.87	51.48
*Ascorbic acid*	41.6	86.49	43.49	89.87

### Anticancer activity

All the synthesized indole derivatives 4(a–u) were screened for anticancer activity using the MTT assay^38–40^ against two human cancer cell lines, *i.e.*, HeLa cells, which are derived from human cervical cancer cells, and MCF-7, which is derived from human breast cancer cells, using *Doxorubicin* as the standard. Based on the screening results, compounds 4c, 4h, 4n, 4q and 4r significantly inhibited the proliferation of the HeLa and MCF-7 cell lines by more than 50% in a dose-dependent manner for 24 h. In the case of anticancer activity, compounds 4c, 4h, 4n, 4q and 4r showed inhibition of 63.48%, 59.45%, 59.39%, 78.49% and 81.47% with IC_50_ values of 18.68, 18.67, 22.34, 16.45 and 15.78, respectively, against HeLa cells. Alternatively, 65.59%, 57.75%, 69.19%, 79.46% and 83.49% inhibition and IC_50_ values of 15.89, 55.17, 84.00, 17.65 and 16.89 were observed against MCF-7 cells, respectively. The other compounds exhibited moderate to good anticancer ability. The anticancer activity results are shown in Table 5.

**Table 5:** Anticancer activities of synthesized compounds 4(a–u)^a^

Concentration of the tested samples, μM		
Compound	HeLa	MCF-7
	IC_50_	IC_50_
4a	60.94	30.41
4b	69.13	62.34
4c	18.68	15.89
4d	20.06	41.49
4e	70.81	70.47
4f	44.09	13.09
4g	57.45	66.06
4h	18.67	55.17
4i	60.18	19.10
4j	13.45	12.99
4k	25.97	55.05
4l	28.90	66.45
4m	23.02	73.82
4n	22.34	84.99
4o	76.93	67.41
4p	56.86	15.79
4q	16.45	17.65
4r	15.78	16.89
4s	19.04	81.21
4t	24.30	76.50
4u	69.69	41.73
DR	67.96	19.79

a DR = doxorubicin.

The structural activity relationship studies of all the synthesized compounds revealed that the presence of a 1,2,3-triazole ring attached to the tetrahydrocarbazole (basic skeleton) is important in promoting the anticancer activity. Products 4c (Ar = 3-COMe), 4h (Ar = 3-COOMe), 4n (Ar = 4-CF_3_), 4q (Ar = 4-OH), and 4r (Ar = 4-NO_2_) as aromatic azide-linked 1,2,3-triazole ring-containing tetrahydrocarbazoles exhibited promising anticancer activities.

### Molecular docking

The most effective compounds, *i.e.*, 4c, 4h, and 4q, as well as the reference medication doxorubicin, were docked into the active site pockets of caspase-3 (PDB ID: 5IAE)^41^ and Human 17-beta-hydroxysteroid dehydrogenase type 1 to better understand the binding interactions between these ligands and cancer cells (PDB ID: 1FDW).^42^ Caspase-3 is a key executioner of apoptosis and plays a vital role in the growth simulation of cancer cells.^43^ 17β-HSD1 is responsible for the production of estrogens, estradiol and 5-androsten-3β,17β-diol, and consequently it is a target of choice for the treatment of estrogen-dependent diseases such as breast cancer and endometriosis by blocking estrogen biosynthesis.^44^ The active site pockets of the target crystal structures were determined using the CAST Web server.^45^ The grid box was configured to cover all the active sites on the crystal structures of the proteins (Table 6). The docking scores of compounds 4c, 4h, and 4q are higher than that of the standard drug *Doxorubicin* with both the target macromolecules (Table 6) (Table 7).

**Table 6:** 3D grid box configuration

PDB	Parameters
5IAE	Receptor = 5iae.pdbqt
	Exhaustiveness = 8
	center_*x* = 10.7092869365
	center_*y* = 2.71092558039
	center_*z* = 161.73714339
	size_*x* = 21.642039768
	size_*y* = 28.2627645563
	size_*z* = 18.3273597312
1FDW	Receptor = 1fdw.pdbqt
	Exhaustiveness = 8
	center_*x* = 44.9201453145
	center_*y* = −0.776505439239
	center_*z* = 35.4279439983
	size_*x* = 27.3194057719
	size_*y* = 34.7070887507
	size_*z* = 37.3789787104

**Table 7:** Binding affinities of compounds 4c, 4h, and 4q, doxorubicin and interacting amino acids of 5IAE and 1FDW

PDB	Compound	Binding affinity (kcal mol^−1^)	Interacting amino acids	
			H-bond	Hydrophobic
5IAE	4c	−8.6	His121	Gly122, Phe128, Thr166, Tyr204, Ser205, Trp206, Phe256
	4h	−8.7	Thr62, Arg207	His121, Gly122, Cys163, Thr166, Tyr204, Ser205, Trp206, Phe256
	4q	−8.8	Glu248	Ser205, Trp206, Arg207, Asn208, Trp214, Ser249, Phe250, Ser251, Phe256
	Doxorubicin	−8.1	Arg64, Arg207	Ser120, His121, Gly122, Gln161, Gly163, Tyr204, Ser205, Trp206, Ser251, Asp253, Phe256
1FDW	4c	−9.5	Tyr218, Ser222	Leu96, Leu149, Gly186, Pro187, Thr190, Ala191, Lys223, Phe226
	4h	−9.4	Tyr218	Val143, Leu149, Pro187, Gln221, Ser222, Lys223, Phe226, Phe259, Met279
	4q	−9.4	Tyr218	Leu96, Ser142, Val143, Leu149, Asn152, Tyr155, Gly186, Ser222, Lys223, Phe226
	Doxorubicin	−8.4	Gly186, Val188, Tyr218, Gln221	Gly94, Leu96, Val143, Leu149, Asn152, Tyr155, Pro187, Phe226, Phe259, Met279, Val283

#### Docking studies with caspase-3

**Fig. 2:**
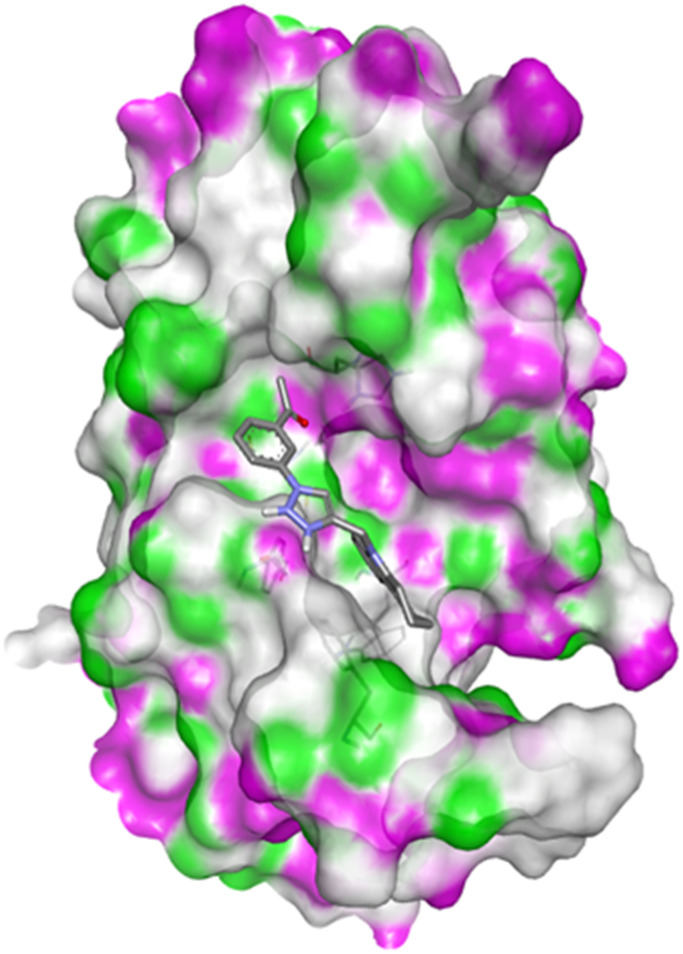
Docking pose of compound 4c with caspase-3.

**Fig. 3:**
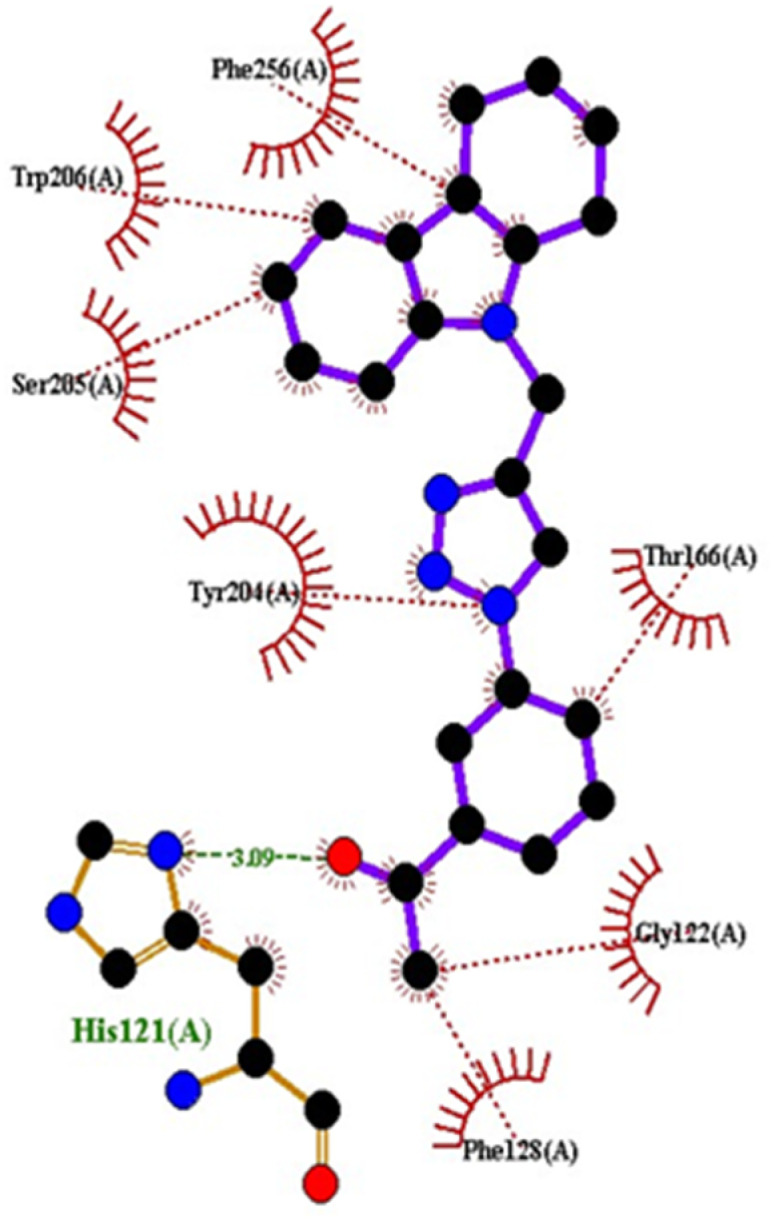
2D interactions of compound 4c with caspase-3.

**Fig. 4:**
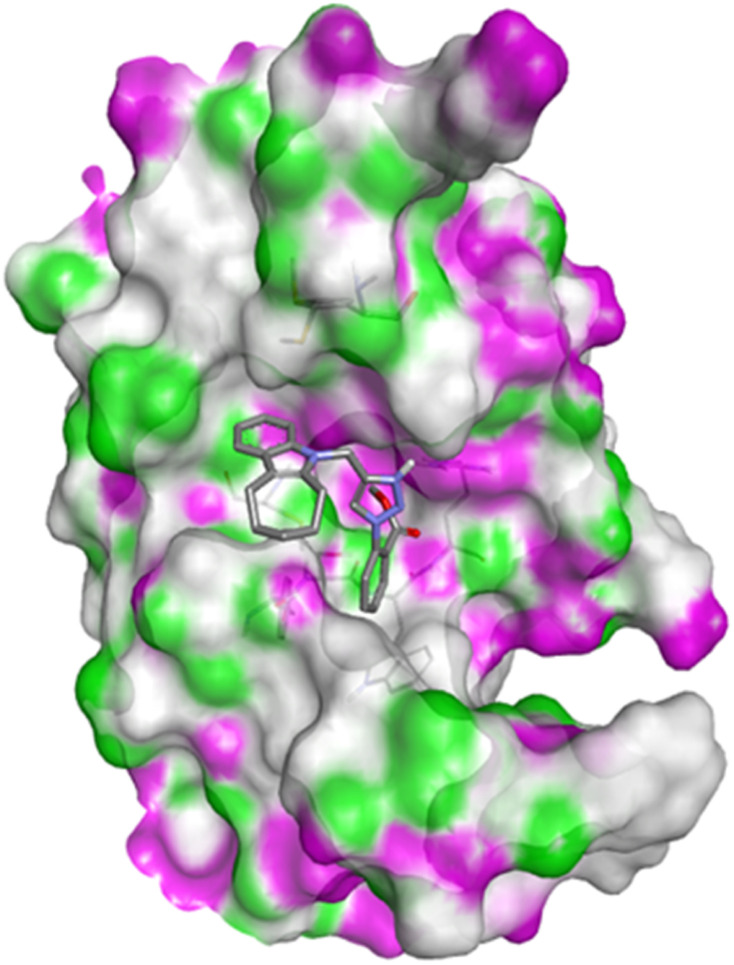
Docking pose of compound 4h with caspase-3.

**Fig. 5:**
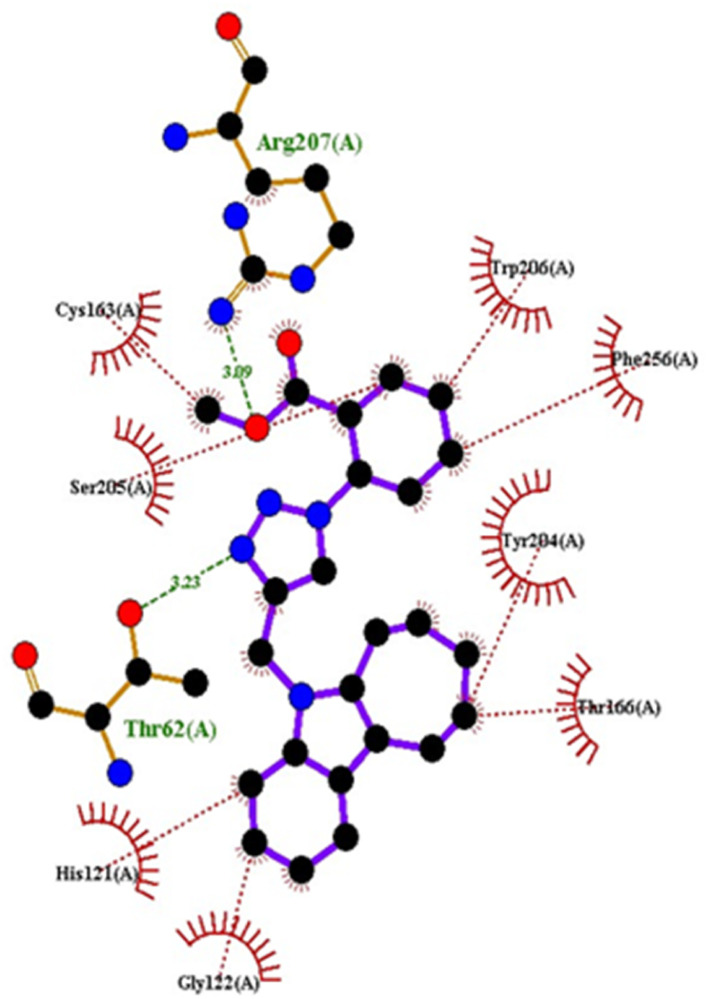
2D interaction of compound 4hwith caspase-3.

**Fig. 6:**
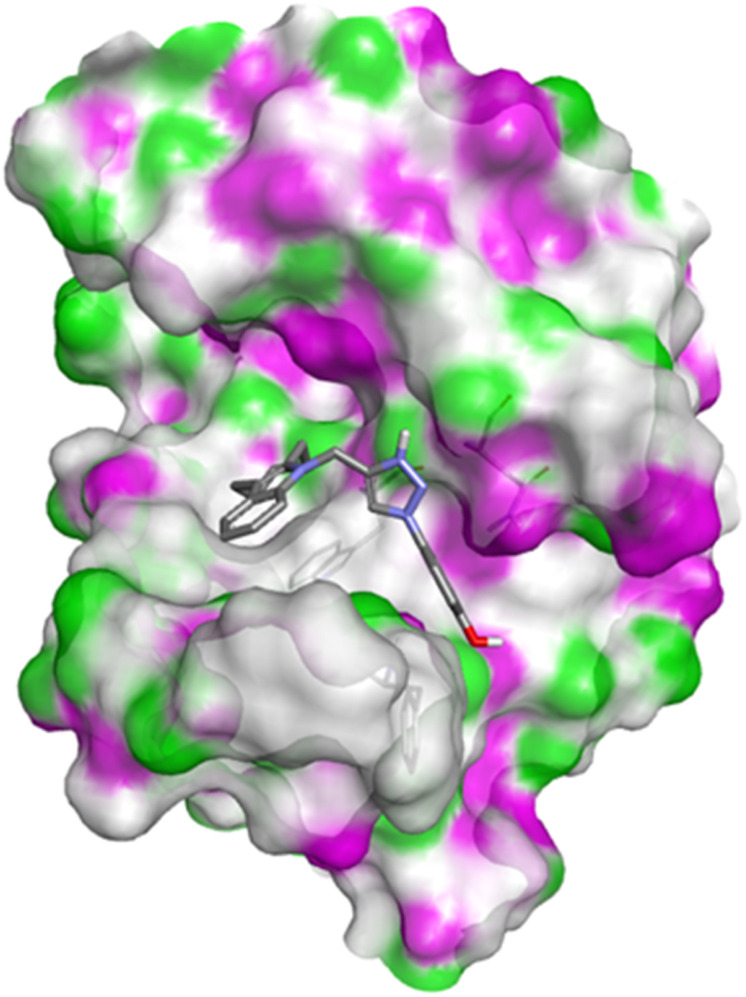
Docking pose of compound 4q with caspase-3.

**Fig. 7:**
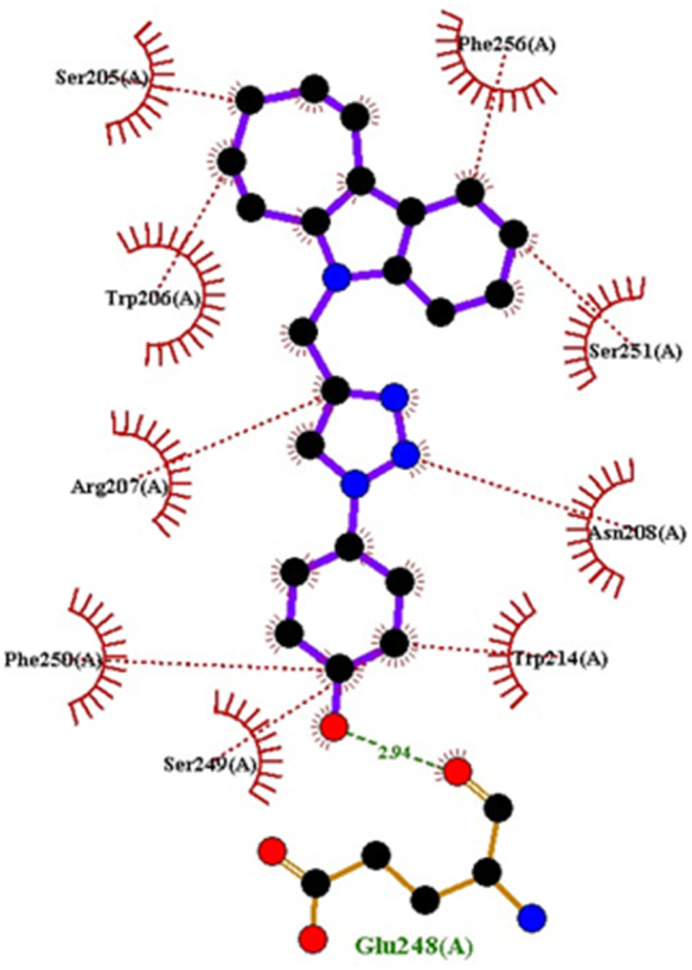
2D interactions of compound 4q with caspase-3.

**Fig. 8:**
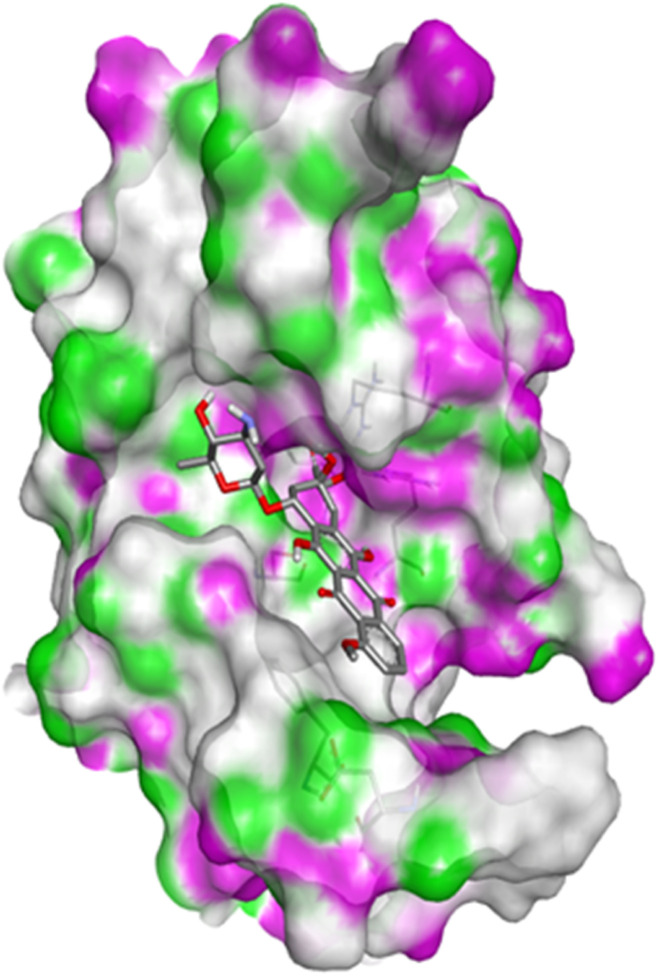
Docking pose of doxorubicin with caspase-3.

**Fig. 9:**
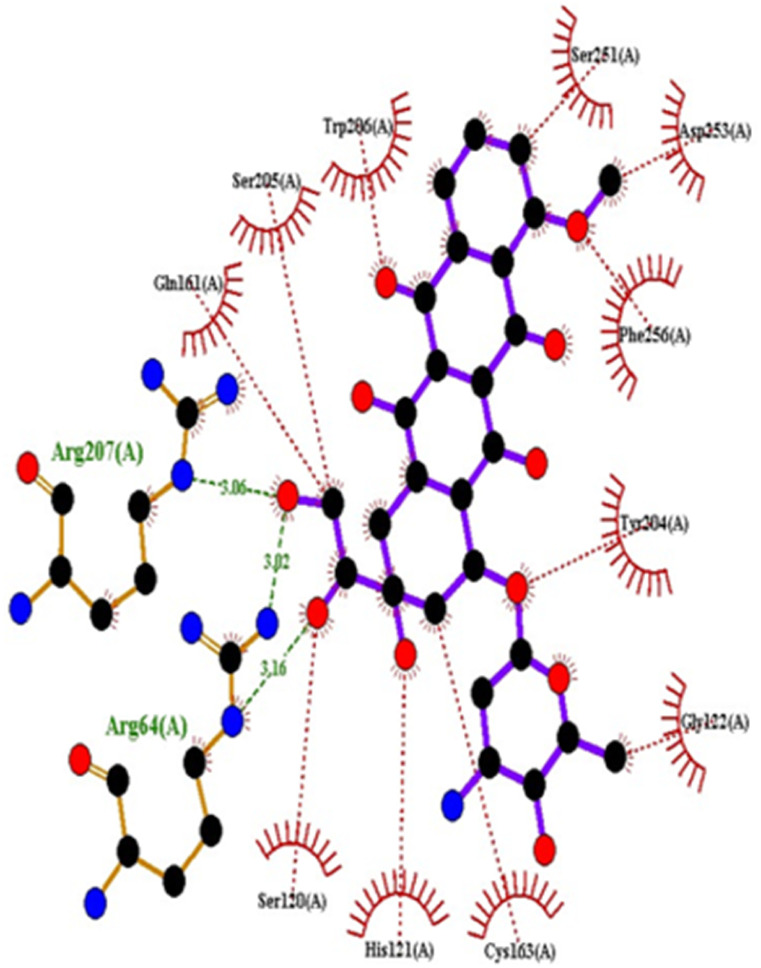
2D interactions of doxorubicin with caspase-3.

Compound 4c has one defined H-bond interaction with His121of caspase-3 with a distance of 3.09 Å. It also exhibits hydrophobic interactions with the amino acids Gly122, Phe128, Thr166, Tyr204, Ser205, Trp206, and Phe256 of 5IAE (Fig. 2 and 3). Compound 4h is involved in two H-interactions with Thr62 (3.23 Å) and Arg207 (3.09 Å) and hydrophobic interactions with His121, Gly122, Cys163, Thr166, Tyr204, Ser205, Trp206, and Phe256 of caspase-3 (Fig. 4 and 5). Compound 4q establishes one H-bond interaction with Glu248 (2.94 Å) and hydrophobic interactions with Ser205, Trp206, Arg207, Asn208, Trp214, Ser249, Phe250, Ser251, and Phe256 of 5IAE (Fig. 6 and 7). The standard drug *Doxorubicin* exhibits three H-bond interactions, *i.e.*, two with Arg64 (3.02 Å and 3.16 Å) and one with Arg207 (3.06 Å) of caspase-3, and it is further involved in hydrophobic interactions with Ser120, His121, Gly122, Gln161, Gly163, Tyr204, Ser205, Trp206, Ser251, Asp253, and Phe256 of 5IAE (Fig. 8 and 9).

#### Docking studies with human 17-beta-hydroxysteroid dehydrogenase type 1

**Fig. 10:**
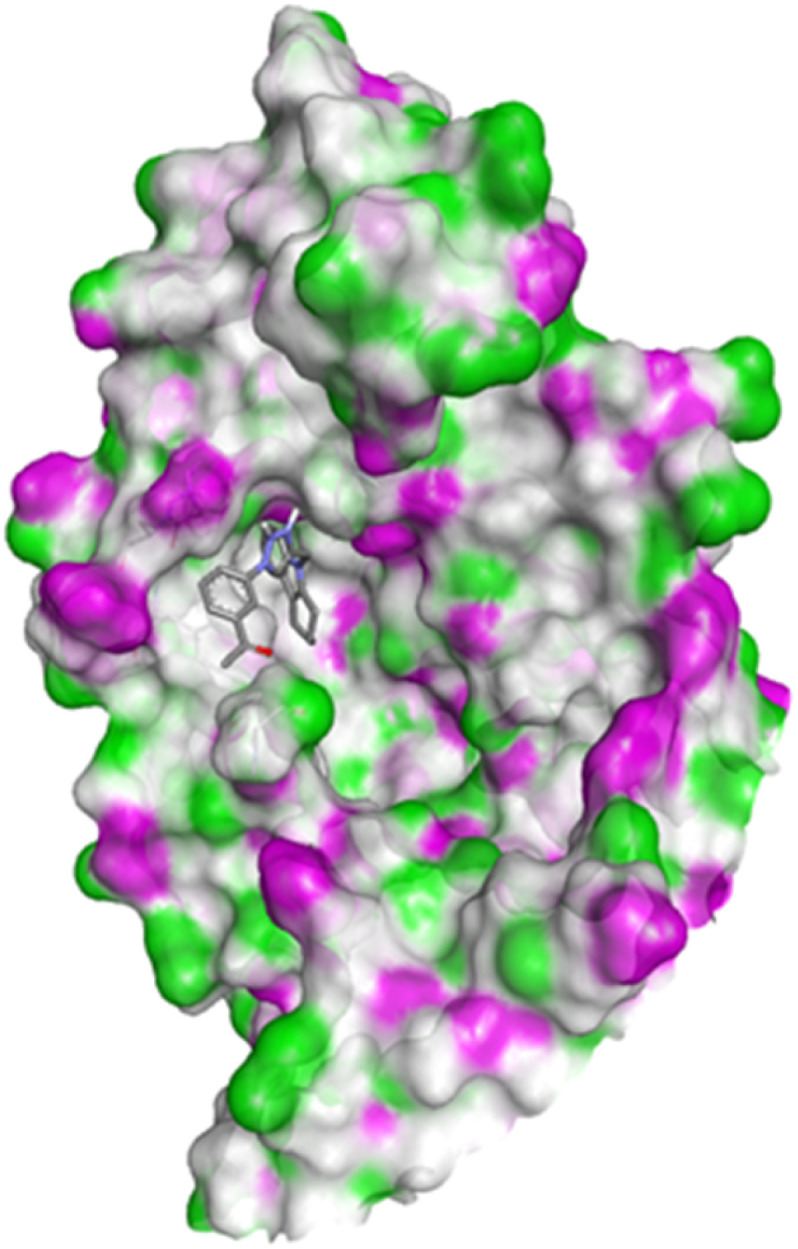
Docking pose of compound 4c with 1FDW.

**Fig. 11:**
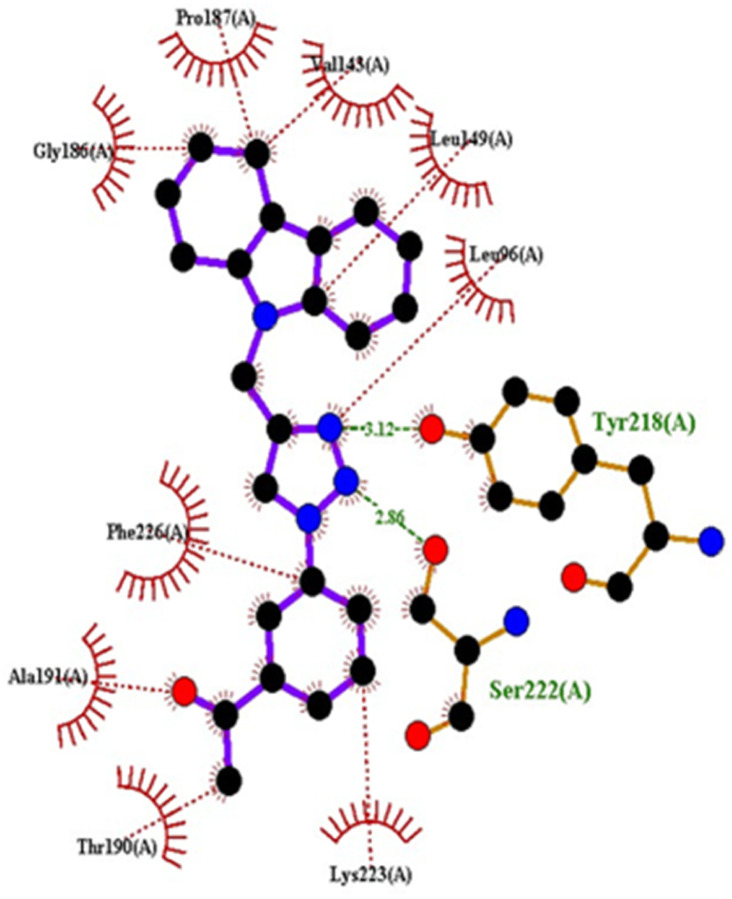
2D interactions compound 4c with 1FDW.

**Fig. 12:**
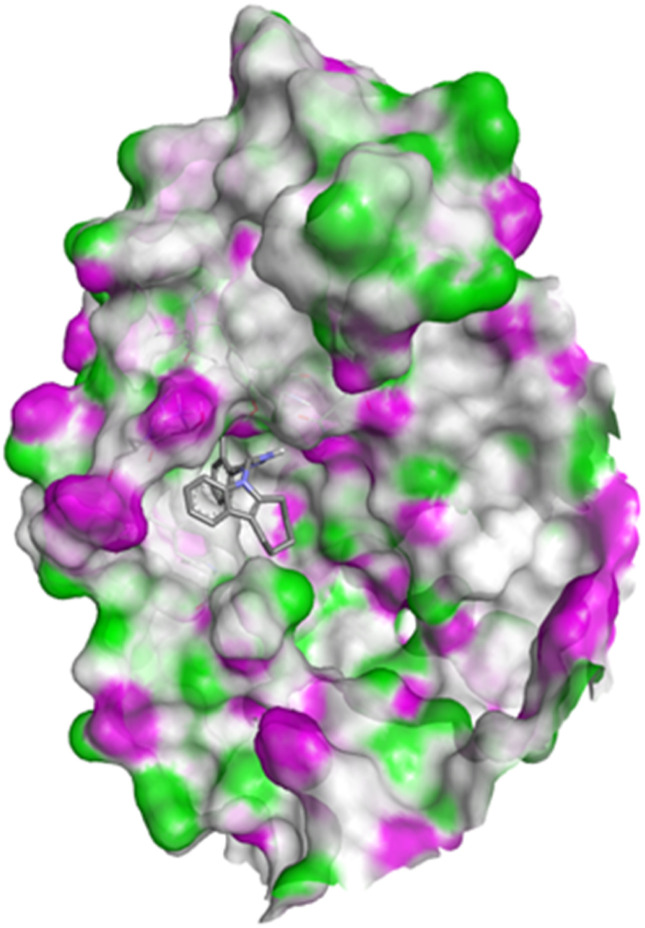
Docking pose of compound 4h with 1FDW.

**Fig. 13:**
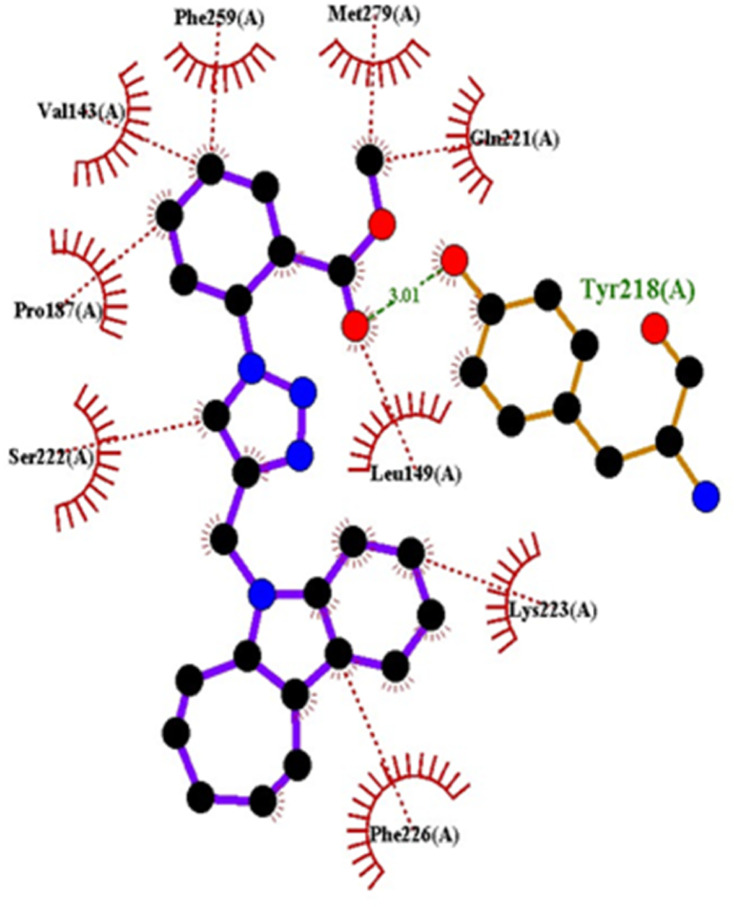
2D interactions of compound 4h with 1FDW.

**Fig. 14:**
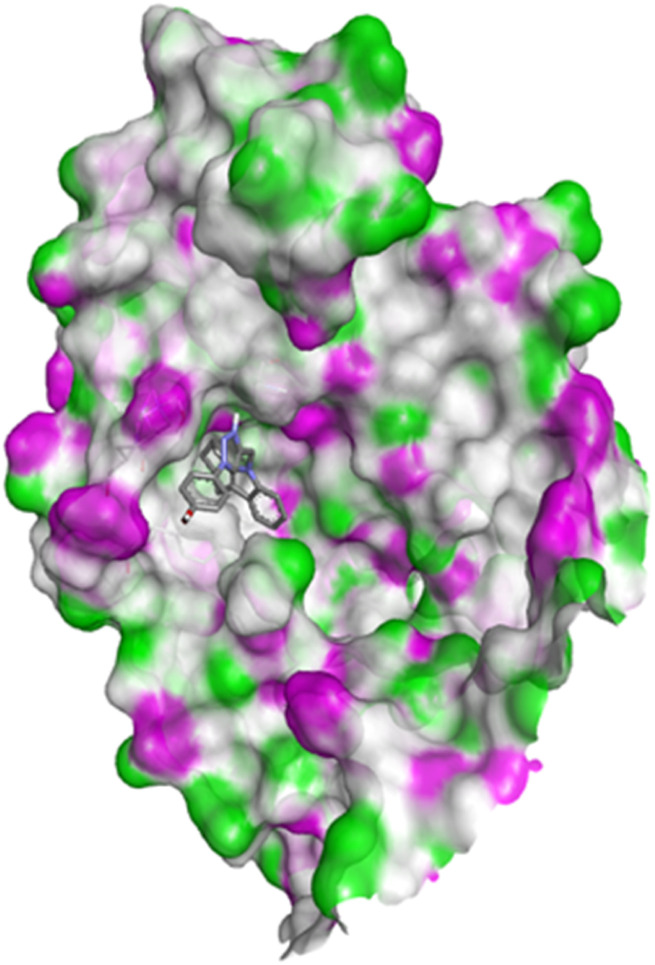
Docking pose of compound 4q with 1FDW.

**Fig. 15:**
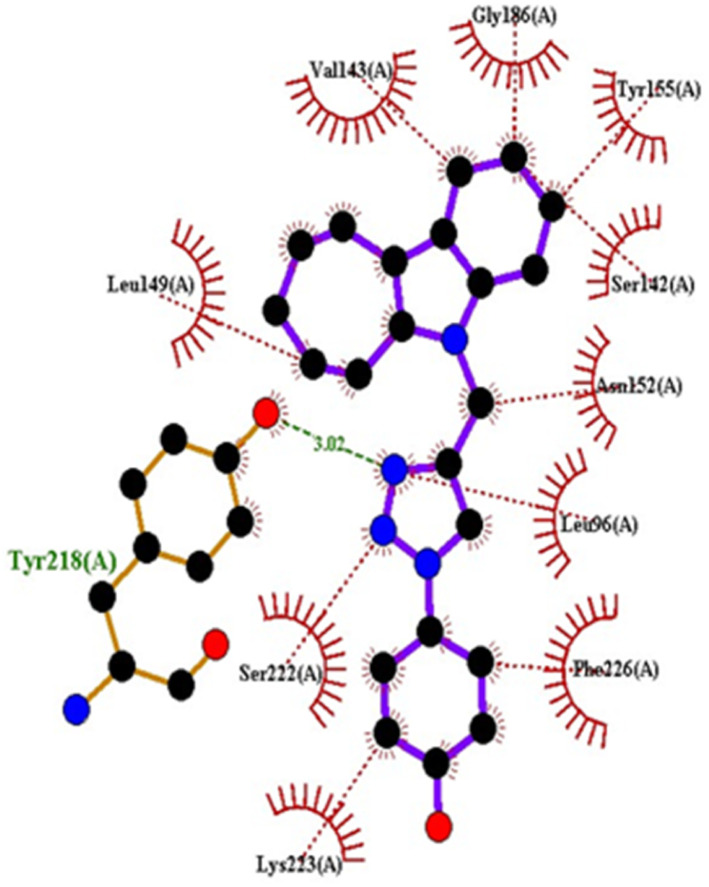
2D interactions of compound 4q with 1FDW.

**Fig. 16:**
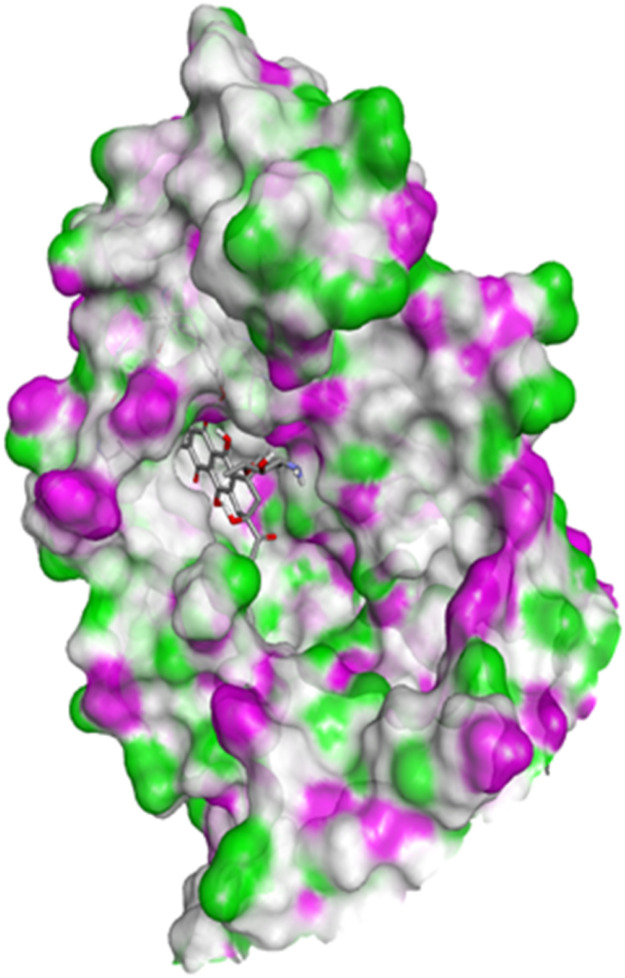
Docking pose of doxorubicin with 1FDW.

**Fig. 17:**
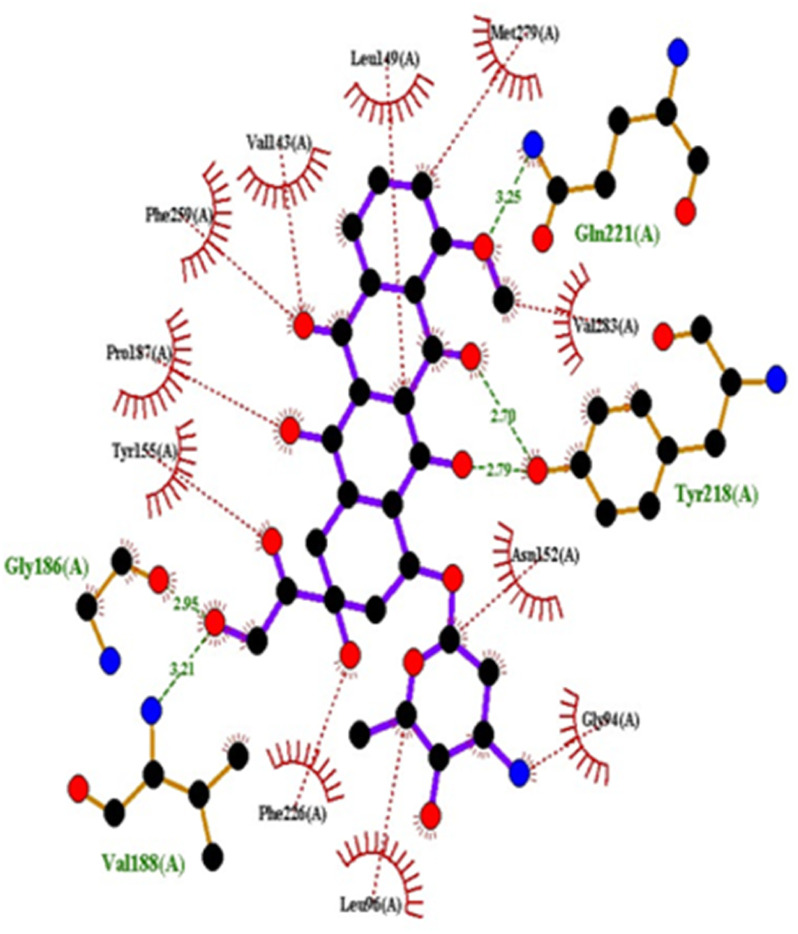
2D interactions of doxorubicin with 1FDW.

Compound 4c exhibited two H-bonds with Tyr218 and Ser222 of 1FDW with a distance of 3.12 Å and 2.06 Å, respectively, and also defined hydrophobic interactions with Leu96, Leu149, Gly186, Pro187, Thr190, Ala191, Lys223 and Phe226 of the target protein (Fig. 10 and 11). Compound 4h shows one H-bond interaction with Tyr218 (3.01 Å) and hydrophobic interactions with Val143, Leu149, Pro187, Gln221, Ser222, Lys223, Phe226, Phe259, and Met279 of dehydrogenase (Fig. 12 and 13). Compound 4q is involved in one H-bond interaction with Tyr218 (3.02 Å) and hydrophobic interactions with Leu96, Ser142, Val143, Leu149, Asn152, Tyr155, Gly186, Ser222, Lys223, and Phe226 of 1FDW (Fig. 14 and 15). The standard drug Doxorubicin is involved in five H-bond interactions with Gly186, Val188, Tyr218 (2), and Gln221 and hydrophobic interactions with Gly94, Leu96, Val143, Leu149, Asn152, Tyr155, Pro187, Phe226, Phe259, Met279, and Val283 of dehydrogenase protein (Fig. 16 and 17).

### Pharmacokinetics evaluation

Oral bioactivity is an important study for the development of new drug candidates. The absorption, distribution, metabolism and excretion properties of the newly synthesized analogues were evaluated using the SwissADME web server protocol.^46^ The calculated pharmacokinetics of the studied compounds are shown in Table 8. Except for compound 4t, all the tested compounds have a molecular weight in the range of 328.41–400.47 g mol^−1^. The molecular weight characteristics of these molecules suggest that they can easily be transported, diffused, and absorbed in the body in a significant manner.^47^ The log *P* values of the compounds were found to be in the range of 2.99–4.36, which meet the essential conditions of Lipinski's rule of five. The calculated number of H-bond acceptors of these all the molecules was less than ten, which is in accordance with ADME as the number of hydrogen bond acceptors must be <10. The pharmacokinetics analysis suggests that compounds 4(a–u) possess a topological polar surface (TPSA) in the range of 99.99–157.65 Å.^47^ The low TPSA values indicate the acceptable range of the results and found to be consistent with previous reports.^48^

**Table 8:** Pharmacokinetics evaluation results of compounds 4(a–u)

Compound	Molecular weight (range ≤ 500)		Rotatable bonds (range 1–10)	H-bond acceptors (range ≤10)	H-bond donors (range ≤5)	TPSA	log *P*_o/w_ (range ≤5)	Molar refractivity (range 40–130)	QP log *S* (solubility)	Lipinski violations	Bioavailability score (range 0.4–0.6)	Synthetic sccessibility
4a		328.41	3	2	0	35.64	3.5	100.04	−4.97	0	0.55	3.19
4b		386.45	5	4	0	61.94	3.53	111.32	−5.02	0	0.55	3.49
4c		370.45	4	3	0	52.71	3.43	110.23	−4.9	0	0.55	3.39
4d		358.44	4	3	0	44.87	3.44	106.53	−5.03	0	0.55	3.3
4e		370.45	4	3	0	52.71	3.53	110.23	−4.9	0	0.55	3.36
4f		346.4	3	3	0	35.64	3.48	99.99	−5.12	1	0.55	3.17
4g		342.44	3	2	0	35.64	3.48	104.84	−5.38	0	0.55	3.32
4h		400.47	5	4	0	61.94	3.66	116.12	−5.43	0	0.55	3.62
4i		360.43	3	3	0	35.64	3.48	104.8	−5.53	1	0.55	3.29
4j		384.47	4	3	0	52.71	3.51	115.04	−5.31	0	0.55	3.52
4k		384.47	4	3	0	52.71	3.55	115.04	−5.31	0	0.55	3.48
4l		376.88	3	2	0	35.64	3.57	109.85	−5.96	1	0.55	3.34
4m		376.88	3	2	0	35.64	3.63	109.85	−5.96	1	0.55	3.35
4n		396.41	4	5	0	35.64	3.72	105.04	−5.8	1	0.55	3.37
4o		436.57	5	2	0	60.94	4.11	130.6	−6.82	1	0.55	3.72
4p		423.48	4	3	0	38.88	3.55	127.04	−5.85	1	0.55	3.4
4q		358.44	3	3	1	55.87	2.99	106.87	−5.23	0	0.55	3.34
4r		386.45	4	4	1	72.94	3.11	111.96	−5.12	0	0.85	3.41
4s		387.43	4	4	0	81.46	3.08	113.66	−5.42	0	0.55	3.44
4t		513.66	6	2	0	64.18	4.36	157.65	−7.54	2	0.17	3.95
4u		358.44	3	3	1	55.87	2.99	106.87	−5.23	0	0.55	3.34

## Experimental

### Materials and methods

All melting points were recorded on a Stuart SMP3 melting-point apparatus and are uncorrected. The IR spectra ῦ in cm^−1^ (KBr) were recorded on a Shimadzu FTIR 8400 S spectrometer. All the microwave irradiation experiments were performed in a CEM Discover microwave system at reaction temperatures at 100 W for 4–6 min and monitored using an equipped IR temperature sensor. ^1^H NMR and ^13^C NMR spectra were measured on a Bruker Avance-400 spectrometer at 400 and 100 MHz, respectively, using tetramethylsilane (TMS) as the internal reference and CDCl_3_ as the solvent. Mass spectra (EI) were recorded on a Finnigan MAT 1020 mass spectrometer in *m*/*z*. All reactions were monitored on silica gel percolated Merck 60 F254 TLC plates and spots were visualized under UV light. The antimicrobial activity study was carried out on nutrient agar medium containing 0.5% peptone, 0.5% NaCl, and 0.3% beef extract (HiMedia, Mumbai, India) and potato dextrose agar (PDA, HiMedia, Mumbai, India) using microbial pathogens such as *Bacillus subtilis* (MTCC 121), *Staphylococcus aureus* (MTCC 96), *Escherichia coli* (MTCC43), *Klebsiella pneumonia* (MTCC 530) and *Pseudomonas aeruginosa* (ATCC-27853, USA) procured from the Institute of Microbial Technology (IMTECH, India) and American Type Culture Collection (ATCC, USA) and the fungal strain *Aspergillus flavus* (NRRL 3357) purchased from Northern Regional Research Laboratory (NRRL, Peoria). The antioxidant activity was investigated using DPPH (0.2 M, Sigma, St. Louis, USA) and HRS mixture (1,10-phenanthroline, 2.5 mM; PBS, pH 7.4; and FeSO_4_, 2.5 mM HiMedia, Mumbai, India). The human cervical cancer cell line (HeLa) and human breast cancer cell line (MCF-7) were procured from the National Center for Cell Sciences (NCCS) Pune, India, and maintained in Dulbecco's modified Eagle medium (DMEM-Gibco, St, Louis, USA) supplemented with antibiotic solution (100×, Thermo Fisher Scientific, St. Louis, USA) and 10% fetal calf serum (FCS-Gibco, St. Louis, USA). The cultured cells were propagated and maintained at 37 °C in a CO_2_ incubator containing 5% CO_2_ and 95% O_2_ atmosphere.

### General procedure for the synthesis of compound (2)

NaH (sodium hydride) (23.38 mmol) was added slowly to a stirred solution of compound (1) (11 mmol) in DMF (dimethylformamide) (20 mL) at 0 °C, and stirring continued for 15 min. Subsequently, propargyl bromide (22 mmol) was added to this mixture and stirred for 3 h at room temperature. After completion of the reaction, the reaction mixture was poured into ice-cold water and extracted with ethyl acetate. The combined organic layer was dried over Na_2_SO_4_ (sodium sulphate) and evaporated under vacuum. The crude reaction mixture was purified by silica gel column chromatography to give compound 2 in 95% yield.

#### 9-(Prop-2-yn-1-yl)-2,3,4,9-tetrahydro-1*H*-carbazole (2)

Yield: 90%; mp: 132–133 °C; IR (KBr, cm^−1^): 3398 (C–H), 1656 (C=C), 1465 (C–C), 1233 (C–N). ^1^H NMR (400 MHz, CDCl_3_) *δ* 7.47 (d, *J* = 7.5 Hz, 1H, Ar–H), 7.34 (d, *J* = 8.0 Hz, 1H, Ar–H), 7.16–7.20 (m, 1H, Ar–H), 7.07–7.12 (m, 1H, Ar–H), 4.76 (d, *J* = 2.5 Hz, 2H, N–CH_2_), 2.70–2.78 (m, 4H, aliphatic-H), 2.23 (t, *J* = 2.5 Hz, 1H, C≡C–H), 1.93–1.99 (m, 2H, aliphatic-H), 1.83–1.89 (m, 2H, aliphatic-H). ^13^C NMR (100 MHz, CDCl_3_) *δ* 135.8, 134.8, 127.6, 120.9, 119.1, 117.9, 110.4, 108.6, 78.5 (C≡C), 71.9 (C≡C), 31.9 (N–CH_2_ carbon), 23.1, 23.0, 21.8, 20.9, mass spectrum, *m*/*z* (*Irel*, %): *calcd 209 observed: 210* [M + H]^+^.

### General procedure for the synthesis of *N*-substituted 1,2,3-triazolylmethyl indole derivatives 4(a–u)

#### Conventional method

CuSO_4_·5H_2_O (0.1514 mmol), sodium ascorbate (0.1314 mmol) and aryl azides 3(a–m) (0.7 mmol) were added to a mixture of 9-(prop-2-yn-1-yl)-2,3,4,9-tetrahydro-1*H*-carbazole (2) (0.7 mmol) in DMF:water (1 : 1 v/v). This mixture was stirred at room temperature for 8–10 h. The reaction was monitored by TLC, and after completion of the reaction, the reaction mixture was poured into ice-cold water and extracted with ethyl acetate. The combined organic layer was dried over Na_2_SO_4_ and evaporated under vacuum, and the crude material was purified by column chromatography on silica gel using hexane/ethyl acetate (7 : 3, v/v) as the eluent to afford compounds 4(a–u).

#### Microwave irradiation method

The procedure was similar to the usual approach described above, with the exception that the mixture was placed in a closed vessel and microwaved for 4–6 min at 100 W. After the reaction was completed, the mixture was poured into ice-cold water and extracted with ethyl acetate under the supervision of TLC. The combined organic layer was vacuum evaporated and dried over Na_2_SO_4_, and the crude substance was purified by column chromatography on silica gel using hexane/ethyl acetate (7 : 3, v/v) as an eluent to afford compounds 4(a–u).

##### 9-((1-Phenyl-1*H*-1,2,3-triazol-4-yl) methyl)-2,3,4,9-tetrahydro-1*H*-carbazole (4a)

Yield: 95%; mp: 112–114 °C; IR (KBr, cm^−1^): 2927 (C–H), 1595 (N=N), 1465 (C=C), 759 (C–N). ^1^H NMR (400 MHz, CDCl_3_) *δ* 7.60 (d, *J* = 7.5 Hz, 2H, Ar–H), 7.33–7.51 (m, 6H, Ar–H), 7.09–7.17 (m, 2H, Ar–H), 5.44 (s, 2H, N–CH_2_), 2.72–2.81 (m, 4H, aliphatic-H), 1.85–1.96 (m, 4H, aliphatic-H). ^13^C NMR (100 MHz, CDCl_3_) *δ* 146.1, 136.8, 135.9, 135.1, 129.5, 128.7, 127.6, 120.9, 120.4, 119.5, 119.1 (Triazole-C), 117.9, 110.3, 108.6, 38.5 (N–CH_2_ carbon), 23.1, 23.0, 22.0, mass spectrum, *m*/*z* (*Irel*, %): *calcd 328 observed*: 329 [M + H]^+^.

##### Methyl-2-(4-((3,4-dihydro-1*H*-carbazol-9(2*H*)-yl)methyl)-1*H*-1,2,3-triazol-1-yl)benzoate (4b)

Yield: 90%; mp: 100–102 °C; IR (KBr, cm^−1^): 3126 (C–H), 1720 (C=O), 1465 (C–C), 1294 (C–N), 754 (C–O). ^1^H NMR (400 MHz, CDCl_3_) *δ* 7.93 (dd, *J* = 7.7, 7.6 Hz, 1H, Ar–H), 7.51–7.60 (m, 2H, Ar–H), 7.47 (d, *J* = 7.7 Hz, 1H, Ar–H), 7.29–7.39 (m, 3H, Ar–H), 7.05–7.5 (m, 2H, Ar–H), 5.44 (s, 2H, N–CH_2_), 3.52 (s, 3H, Ar–H), 2.82 (t, *J* = 5.9, 6.2 Hz, 2H, aliphatic-H), 2.72 (t, *J* = 5.9 Hz, 2H, aliphatic-H), 1.93–1.98 (m, 2H, aliphatic-H), 1.83–1.88 (m, 2H, aliphatic-H). ^13^C NMR (100 MHz, CDCl_3_) *δ* 165.4, 145.1, 135.9, 135.8, 135.1, 132.5, 131.1, 129.8, 127.7, 127.2, 126.6, 123.4, 120.8, 119.0 (Triazole-C), 117.8, 110.2, 108.6, 52.3 (Ar–C), 38.5 (N–CH_2_ carbon), 23.1, 23.0, 22.0, 21.0, mass spectrum, *m*/*z* (*Irel*, %): *calcd*: *386* observed: 387 [M + H]^+^.

##### 1-(3-(4-((3,4-Dihydro-1*H*-carbazol-9(2*H*)-yl)methyl)-1*H*-1,2,3-triazol-1-yl)phenyl)ethanone (4c)

Yield: 91%; mp: 116–118 °C; IR (KBr, cm^−1^): 3149, 2929, 2833 (C–H), ^1^H NMR (400 MHz, CDCl_3_) *δ* 8.14 (t, *J* = 1.8 Hz, 3H, Ar–H), 7.86–7.96 (m, 2H, Ar–H), 7.55–7.58 (m, 2H, Ar–H), 7.49 (d, *J* = 7.7 Hz, 1H, Ar–H), 7.33 (d, *J* = 8.1 Hz, 1H, Ar–H), 7.08–7.17 (m, *2*H, Ar–H), 5.44 (s, 2H, N–CH_2_), 2.80 (t, *J* = 6.2, 5.9 Hz, 2H, aliphatic-H), 2.74 (t, *J* = 6.1, 5.9 Hz, 2H, aliphatic-H), 2.62 (s, 3H, Ar–H), 1.94–1.98 (m, 2H, aliphatic-H), 1.85–1.89 (m, 2H, aliphatic-H). ^13^C NMR (100 MHz, CDCl_3_) *δ* 196.6, 146.5, 138.3, 137.1, 135.9, 135.1, 130.0, 128.3, 127.6, 124.7, 121.0, 119.6, 119.5, 119.2 (Triazole-C), 118.0, 110.4, 108.5, 38.5 (N–CH_2_ carbon), 26.7, 23.1, 23.0, 22.0, 21.0, mass spectrum, *m*/*z* (*Irel*, %): *calcd*: *370 observed:* 371 [M + H]^+^.

##### 9-((1-(4-Methoxyphenyl)-1*H*-1,2,3-triazol-4-yl)methyl)-2,3,4,9-tetrahydro-1*H*-carbazole (4d)

Yield: 83%; mp: 155–157 °C; IR (KBr, cm^−1^): 3134 (C–H), 2836, 1602, 1504, 1049 (C–O). ^1^H NMR (400 MHz, CDCl_3_) *δ* 7.61–7.67 (m, 2H, Ar–H), 7.48 (d, *J* = 7.7 Hz, 1H, Ar–H), 7.34–7.40 (m, 2H, Ar–H), 6.98–7.17 (m, 4H, Ar–H), 5.43 (s, 2H, N–CH_2_), 3.77 (s, 3H, Ar–H), 2.83 (t, *J* = 6.0, 6.2 Hz, 2H, aliphatic-H), 2.73 (t, *J* = 6.0 Hz, 2H, aliphatic-H), 1.83–1.99 (m, 4H, aliphatic-H). ^13^C NMR (100 MHz, CDCl_3_) *δ* 151.0, 144.5, 135.3, 130.1, 125.4, 123.7, 121.0, 120.7, 119.3 (Triazole-C), 118.9, 117.8, 112.0, 110.1, 108.8, 55.8 (Ar–C), 38.6 (N–CH_2_ carbon), 23.2, 23.1, 22.1, 21.0, mass spectrum, *m*/*z* (*Irel*, %): *calcd*: *358 observed*: *359* [M + H]^+^.

##### 1-(4-(4-((3,4-Dihydro-1*H*-carbazol-9(2*H*)-yl)methyl)-1*H*-1,2,3-triazol-1-yl)phenyl)ethanone (4e)

Yield: 87%; mp: 182–184 °C; IR (KBr, cm^−1^): 3134 (C–H), 3043, 2929, 2852, 1674. ^1^H NMR (400 MHz, CDCl_3_) *δ* 8.04 (d, *J* = 8.6 Hz, 2H, Ar–H), 7.74 (d, *J* = 8.6 Hz, 2H, Ar–H), 7.54 (s, 1H, triazole-H), 7.50 (d, *J* = 7.7 Hz, 1H, Ar–H), 7.32 (d, *J* = 8.0, 1H, Ar–H), 7.09–7.18 (m, 2H, Ar–H), 5.45 (s, 2H, N–CH_2_), 2.79 (t, *J* = 5.7, 6.1 Hz, 2H, aliphatic-H), 2.74 (t, *J* = 6.1, 5.6 Hz, 2H, aliphatic-H), 2.62 (s, 3H, Ar–H), 1.93–1.98 (m, 2H, aliphatic-H), 1.85–1.89 (m, 2H, aliphatic-H). ^13^C NMR (100 MHz, CDCl_3_) *δ* 196.4, 140.0, 136.8, 136.7, 135.9, 135.1, 129.9, 127.7, 126.0, 121.0, 119.9, 119.2 (Triazole-C), 118.0, 110.4, 108.5, 38.5 (N–CH_2_ carbon), 26.6, 23.1, 23.0, 22.0, 21.0, mass spectrum, *m*/*z* (*Irel*, %): *calcd*: *370 observed*: *371* [M + H]^+^.

##### 9-((1-(2-Fluorophenyl)-1*H*-1,2,3-triazol-4-yl)methyl)-2,3,4,9-tetrahydro-1*H*-carbazole (4f)

Yield: 90%; mp: 114–116 °C; IR (KBr, cm^−1^): 3136 (C–H), 1609, 1508, 1468, 1230 (C–F). ^1^H NMR (400 MHz, CDCl_3_) *δ* 7.83 (td, *J* = 7.8, 1.7 Hz, 1H, Ar–H), 7.61 (d, *J* = 2.7 Hz, 1H, Ar–H), 7.48 (d, *J* = 7.7 Hz, 1H, Ar–H), 7.34–7.41 (m, 2H, Ar–H), 7.04–7.27 (m, 4H, Ar–H), 5.44 (s, 2H, N–CH_2_), 2.82 (t, *J* = 6.1 Hz, 2H, aliphatic-H), 2.73 (t, *J* = 6.0 Hz, 2H, aliphatic-H), 1.92–1.99 (m, 2H, aliphatic-H), 1.83–1.90 (m, 2H, aliphatic-H). ^13^C NMR (100 MHz, CDCl_3_) *δ* 154.5, 152.0, 145.6, 135.9, 135.1, 129.7, 127.6, 124.8, 122.8, 120.9, 119.0 (Triazole-C), 117.9, 116.9, 116.7, 110.3, 108.6, 38.4 (N–CH_2_ carbon), 23.1, 23.0, 22.0, 21.0, mass spectrum, *m*/*z* (*Irel*, %): *calcd*: *346 observed*: *347* [M + H]^+^.

##### 5-((1-Phenyl-1*H*-1,2,3-triazol-4-yl)methyl)-5,6,7,8,9,10-hexahydrocyclohepta[*b*]indole (4g)

Yield: 85%; mp: 146–148 °C; IR (KBr, cm^−1^): 3118, 3076 (C–H), 3045, 2912, 1463 (C=C). ^1^H NMR (400 MHz, CDCl_3_) *δ* 7.57–7.60 (m, 2H, Ar–H), 7.51 (d, *J* = 7.3 Hz, 1H, Ar–H), 7.41–7.46 (m, 3H, Ar–H), 7.35–7.39 (m, 1H, Ar–H), 7.32 (d, *J* = 7.9 Hz, 1H, Ar–H), 7.08–7.16 (m, 2H, Ar–H), 5.50 (s, 2H, N–CH_2_), 2.93 (t, *J* = 5.6 Hz, 2H, aliphatic-H), 2.85 (t, *J* = 5.6 Hz, 2H, aliphatic-H), 1.87–1.92 (m, 2H, aliphatic-H), 1.74–1.79 (m, 4H, aliphatic-H). ^13^C NMR (100 MHz, CDCl_3_) *δ* 146.4, 138.4, 136.7, 135.2, 129.6, 128.7, 128.2, 120.8, 120.4, 119.5, 119.1 (Triazole-C), 117.7, 114.6, 108.6, 38.8 (N–CH_2_ carbon), 31.5, 28.2, 27.1, 26.4, 24.3, mass spectrum, *m*/*z* (*Irel*, %): *calcd*: *342 observed*: *343* [M + H]^+^.

##### Methyl-2-(4-((7,8,9,10-tetrahydrocyclohepta[*b*]indol-5(6*H*)-yl)methyl)-1*H*-1,2,3-triazol-1-yl) benzoate (4h)

Yield: 85%; mp: 126–128 °C; IR (KBr, cm^−1^): 3128 (C–H), 3049, 2839, 1718 (C=O), 1467 (C–C), 1294 (C–N). ^1^H NMR (400 MHz, CDCl_3_) *δ* 7.94 (dd, *J* = 7.7, 7.5 Hz, 1H, Ar–H), 7.48–7.61 (m, 3H, Ar–H), 7.32–7.38 (m, 2H, Ar–H), 7.27 (s, 1H, triazole-H), 7.06–7.16 (m, 2H, Ar–H), 5.52 (s, 2H, N–CH_2_), 3.53 (s, 3H, Ar–H), 2.96 (t, 2H, *J* = 5.5 Hz, aliphatic-H), 2.83 (t, 2H, *J* = 5.7, 5.5 Hz, aliphatic-H), 1.86–1.92 (m, 2H, aliphatic-H), 1.74–1.81 (m, 4H, aliphatic-H). ^13^C NMR (100 MHz, CDCl_3_) *δ* 165.4, 145.4, 138.4, 135.8, 135.1, 132.5, 131.1, 129.8, 128.2, 127.2, 126.6, 123.3, 120.7, 119.1 (Triazole-C), 117.7, 114.5, 108.7, 52.3 (Ar–C), 38.7 (N–CH_2_ carbon), 31.5, 28.2, 27.0, 26.3, 24.2, mass spectrum, *m*/*z* (*Irel*, %): *calcd*: *400 observed*: *401* [M + H]^+^.

##### 5-((1-(2-Fluorophenyl)-1*H*-1,2,3-triazol-4-yl)methyl)-5,6,7,8,9,10hexahydrocyclohepta[*b*]indole (4i)

Yield: 72%; mp: 120–122 °C; IR (KBr, cm^−1^): 3136, 3091 (C–H), 1467 (C–C), 1232 (C–F). ^1^H NMR (400 MHz, CDCl_3_) *δ* 7.83 (t, *J* = 7.9, 7.6 Hz, 1H, Ar–H), 7.53 (s, 1H, triazole-H), 7.49 (d, *J* = 7.7 Hz, 1H, Ar–H), 7.32–7.39 (m, 2H, Ar–H), 7.19–7.27 (m, 2H, Ar–H), 7.06–7.15 (m, 2H, Ar–H), 5.50 (s, 2H, N–CH_2_), 2.94 (t, *J* = 5.3 Hz, 2H, aliphatic-H), 2.84 (t, *J* = 5.3 Hz, 2H, aliphatic-H), 1.87–1.92 (m, 2H, aliphatic-H), 1.75–1.80 (m, 4H, aliphatic-H). ^13^C NMR (100 MHz, CDCl_3_) *δ* 154.5, 152.0, 146.0, 138.4, 135.2, 130.1, 128.2, 124.8, 122.6, 120.7, 119.1 (Triazole-C), 117.7, 116.9, 116.7, 114.7, 108.7, 38.7 (N–CH_2_ carbon), 31.5, 28.2, 27.0, 26.4, 24.3, mass spectrum, *m*/*z* (*Irel*, %): *calcd*: *360 observed*: *361* [M + H]^+^.

##### 1-(3-(4-((7,8,9,10-Tetrahydrocyclohepta[*b*]indol-5(6*H*)-yl)methyl)-1*H*-1,2,3-triazol-1-yl)phenyl)ethanone (4j)

Yield: 76%; mp: 173–175 °C; IR (KBr, cm^−1^): 3142, 3049 (C–H), 1676 (C=O), 1589. ^1^H NMR (400 MHz, CDCl_3_) *δ* 8.14 (t, *J* = 1.8, 1H, Ar–H), 7.94–7.97 (m, 1H, Ar–H), 7.84–7.88 (m, 1H, Ar–H), 7.50–7.58 (m, 3H, Ar–H), 7.32 (d, *J* = 7.5 Hz, 1H, Ar–H), 7.09–7.17 (m, 2H, Ar–H), 5.51 (s, 2H, N–CH_2_), 2.94 (t, *J* = 5.5, 5.7 Hz, 2H, aliphatic-H), 2.85 (t, *J* = 5.6 Hz, 2H, aliphatic-H), 2.62 (s, 3H, Ar–H), 1.88–1.92 (m, 2H, aliphatic-H), 1.76–1.80 (m, 4H, aliphatic-H). ^13^C NMR (100 MHz, CDCl_3_) *δ* 196.6, 146.8, 138.4, 137.2, 135.2, 130.1, 128.4, 128.2, 124.7, 120.9, 119.7, 119.6, 119.3 (Triazole-C), 117.9, 114.8, 108.7, 38.8 (N–CH_2_ carbon), 31.5, 28.3, 27.1, 26.7, 26.4, 24.3, mass spectrum, *m*/*z* (*Irel*, %) *calcd*: *384 observed*: *385* [M + H]^+^.

##### 1-(4-(4-((7,8,9,10-Tetrahydrocyclohepta[*b*]indol-5(6*H*)-yl)methyl)-1*H*-1,2,3-triazol-1-yl)phenyl)ethanone (4k)

Yield: 92%; mp: 138–140 °C; IR (KBr, cm^−1^): 3047 (C–H), 2921, 1605 (C=O), 1467 (C–C). ^1^H NMR (400 MHz, CDCl_3_) *δ* 8.04 (d, *J* = 8.8 Hz, 2H, Ar–H), 7.73 (d, *J* = 8.8 Hz, 2H, Ar–H), 7.49–7.53 (m, 2H, Ar–H); 7.31 (d, *J* = 7.3 Hz, 1H, Ar–H), 7.09–7.17 (m, 2H, Ar–H), 5.51 (s, 2H, N–CH_2_), 2.93 (t, *J* = 5.6 Hz, 2H, aliphatic-H), 2.85 (t, *J* = 5.6 Hz, 2H, aliphatic-H), 2.61 (s, 3H, Ar–H), 1.87–1.92 (m, 2H, aliphatic-H), 1.74–1.80 (m, 4H, aliphatic-H). ^13^C NMR (100 MHz, CDCl_3_) *δ* 196.5, 146.9, 139.8, 138.4, 136.8, 135.2, 130.0, 128.3, 120.9, 119.9, 119.4, 119.3 (Triazole-C), 117.9, 114.8, 108.7, 38.7 (N–CH_2_ carbon), 31.5, 28.3, 27.1, 26.6, 26.4, 24.3, mass spectrum, *m*/*z* (*Irel*, %) *calcd*: *384 observed*: *385* [M + H]^+^.

##### 5-((1-(4-Chlorophenyl)-1*H*-1,2,3-triazol-4-yl)methyl)-5,6,7,8,9,10-hexahydrocyclohepta[*b*]indole (4l)

Yield: 90%; mp: 168–170 °C; IR (KBr, cm^−1^): 3137, 2847 (C–H), 1230 (C–N), 736 (C–Cl). ^1^H NMR (400 MHz, CDCl_3_) *δ* 7.50–7.58 (m, 3H, Ar–H), 7.39–7.44 (m, 3H, Ar–H), 7.30 (d, *J* = 7.3 Hz, 1H, Ar–H), 7.09–7.17 (m, 2H, Ar–H), 5.50 (s, 2H, N–CH_2_), 2.92 (t, *J* = 5.7, 5.6, 2H, aliphatic-H), 2.85 (t, *J* = 5.6 Hz, 2H, aliphatic-H), 1.87–1.93 (m, 2H, aliphatic-H), 1.73–1.80 (m, 4H, aliphatic-H). ^13^C NMR (100 MHz, CDCl_3_) *δ* 146.7, 138.4, 135.3, 135.2, 134.5, 129.8, 128.3, 121.5, 120.9, 119.4, 119.3 (Triazole-C), 117.9, 114.7, 108.7, 38.8 (N–CH_2_ carbon), 31.5, 28.3, 27.1, 26.4, 24.3, mass spectrum, *m*/*z* (*Irel*, %): *calcd*: *376 observed*: *377* [M + H]^+^.

##### 5-((1-(2-Chlorophenyl)-1*H*-1,2,3-triazol-4-yl)methyl)-5,6,7,8,9,10-hexahydrocyclohepta[*b*]indole (4m)

Yield: 96%; mp: 150–152 °C; IR (KBr, cm^−1^): 3422, 2911, 1464 (C–C), 730 (C–Cl). ^1^H NMR (400 MHz, CDCl_3_) *δ* 7.47–7.52 (m, 3H, Ar–H), 7.31–7.43 (m, 4H, Ar–H), 7.06–7.15 (m, 2H, Ar–H), 5.53 (s, 2H, N–CH_2_), 2.94 (t, *J* = 5.6, 5.7 Hz, 2H, aliphatic-H), 2.84 (t, *J* = 5.6 Hz, 2H, aliphatic-H), 1.86–1.92 (m, 2H, aliphatic-H), 1.74–1.81 (m, 4H, aliphatic-H). ^13^C NMR (100 MHz, CDCl_3_) *δ* 145.5, 138.4, 135.2, 134.6, 130.7, 130.6, 128.5, 128.1, 127.7, 127.6, 123.5, 120.7, 119.1 (Triazole-C), 117.7, 114.7, 108.7, 38.7 (N–CH_2_ carbon), 31.5, 28.2, 27.0, 26.4, 24.3, mass spectrum, *m*/*z* (*Irel*, %) *calcd*: *376 observed*: *377* [M + H]^+^.

##### 9-((1-(4-(Trifluoromethyl)phenyl)-1*H*-1,2,3-triazol-4-yl)methyl)-2,3,4,9-tetrahydro-1*H*-carbazole (4n)

Yield: 92%; mp: 104–106 °C; IR (KBr, cm^−1^): 3045, 2980, 1459, 1232 (C–F). ^1^H NMR (400 MHz, CDCl_3_) *δ* 7.90 (s, 1H, Ar–H), 7.81 (d, *J* = 8.0 Hz, 1H, Ar–H), 7.65 (d, *J* = 7.8 Hz, 1H, Ar–H), 7.58 (t, *J* = 7.9 Hz, 1H, Ar–H), 7.53–7.48 (m, 2H, Ar–H), 7.32 (d, *J* = 8.0 Hz, 1H, Ar–H), 7.19–7.08 (m, 2H, Ar–H), 5.44 (s, 2H, N–CH_2_), 2.76 (dt, *J* = 20.6, 5.8 Hz, 4H, aliphatic-H), 1.98–1.83 (m, 4H, aliphatic-H). ^13^C NMR (100 MHz, CDCl_3_) *δ* 146.7, 137.1, 136.0, 135.1, 132.2, 130.4, 127.8, 124.6, 123.5, 121.1, 119.5, 119.3 (Triazole-C), 118.0, 110.5, 108.5, 38.5 (N–CH_2_ carbon), 23.6, 23.1, 22.0, 21.0, mass spectrum, *m*/*z* (*Irel*, %) *calcd*: *396 observed*: *397* [M + H]^+^.

##### 9-((1-(2-(Phenylthio)phenyl)-1*H*-1,2,3-triazol-4-yl)methyl)-2,3,4,9-tetrahydro-1*H*-carbazole (4o)

Yield: 94%; mp: 131–132 °C; IR (KBr, cm^−1^): 3423, 3073, 1547, 695. ^1^H NMR (400 MHz, CDCl_3_) *δ* 7.48 (d, *J* = 7.5 Hz, 1H, Ar–H), 7.43–7.39 (m, 2H, Ar–H), 7.32 (dd, *J* = 9.4, 4.6 Hz, 3H, Ar–H), 7.29–7.26 (m, 1H, Ar–H), 7.25–7.19 (m, 3H, Ar–H), 7.16–7.12 (m, 3H, Ar–H), 7.09 (dd, *J* = 10.8, 4.0 Hz, 1H, Ar–H), 5.41 (s, 2H, N–CH_3_), 2.75 (dt, *J* = 23.1, 5.8 Hz, 4H, aliphatic-H), 1.96–1.82 (m, 4H, aliphatic-H). ^13^C NMR (100 MHz, CDCl_3_) *δ* 145.0, 136.6, 136.0, 135.2, 133.1, 132.6, 132.5, 131.7, 130.2, 129.4, 127.9, 127.8, 127.7, 127.0, 123.6, 120.9, 119.0 (Triazole-C), 117.9, 110.2, 108.7, 38.5 (N–CH_2_ carbon), 23.2, 23.1, 22.1, 21.0, mass spectrum, *m*/*z* (*Irel*, %) *calcd*: *436 observed:* 437 [M + H]^+^.

##### 9-((1-(2-Fluorophenyl)-1*H*-1,2,3-triazol-4-yl)methyl)-3-phenyl-2,3,4,9-tetrahydro-1*H*-carbazole (4p)

Yield: 90%; mp: 145–147 °C; IR (KBr, cm^−1^): 3135, 2380 (C–C), 1453, 1230. ^1^H NMR (400 MHz, CDCl_3_) *δ* 7.84 (tt, *J* = 9.3, 4.6 Hz, 1H, Ar–H), 7.72 (m, *J* = 13.4, 9.6, 2.2 Hz, 1H, Ar–H), 7.65 (d, *J* = 2.7 Hz, 1H, Ar–H), 7.47 (d, *J* = 7.7 Hz, 1H, Ar–H), 7.40–7.37 (m, 2H, Ar–H), 7.33 (d, *J* = 1.9 Hz, 1, Ar–H), 7.31–7.15 (m, 6H, Ar–H), 7.10 (dd, *J* = 11.0, 3.9 Hz, 1H, Ar–H), 5.47 (s, 2H, N–CH_2_), 3.12–3.03 (m, 2H, aliphatic-H), 3.01–2.94 (m, 2H, aliphatic-H), 2.88–2.79 (m, 1H, aliphatic-H), 2.31–2.08 (m, 2H, aliphatic-H). ^13^C NMR (100 MHz, CDCl_3_) *δ* 154.5, 146.5, 136.3, 134.8, 130.3, 130.2, 145.5, 128.4, 128.8, 127.0, 126.2, 124.9, 121.2, 119.3, 119.0 (Triazole-C), 118.0, 117.0, 116.8, 110.3, 108.8, 40.9, 38.6 (N–CH_2_ carbon), 30.3, 29.3, 22.3, mass spectrum, *m*/*z* (*Irel*, %) *calcd*: *422 observed*: *423* [M + H]^+^.

##### 4-(4-((7,8,9,10-Tetrahydrocyclohepta[*b*]indol-5(6*H*)-yl)methyl)-1*H*-1,2,3-triazol-1-yl)phenol (4q)

Yield: 85%; mp: 160–162 °C; IR (KBr, cm^−1^): 3548, 3085, 2850, 3085, 1300 (C–OH). ^1^H NMR (400 MHz, CDCl_3_) *δ* 7.52 (d, *J* = 7.2 Hz, 1H, Ar–H), 7.45 (d, *J* = 8.8 Hz, 2H, Ar–H), 7.32 (d, *J* = 6.9 Hz, 2H, Ar–H), 7.18–7.08 (m, 2H, Ar–H), 6.89 (d, *J* = 8.8 Hz, 2H), 5.49 (s, 2H, N–CH_2_), 5.30 (s, 1H, C–OH), 2.95–2.90 (m, 2H, aliphatic-H), 2.87–2.82 (m, 2H, aliphatic-H), 1.89 (d, *J* = 5.0 Hz, 2H, aliphatic-H), 1.76 (d, *J* = 4.8 Hz, 4H, aliphatic-H). ^13^C NMR (100 MHz, CDCl_3_) *δ* 156.1, 146.2, 146.5, 138.4, 129.0, 128.2, 127.8, 122.4, 120.8, 119.7, 119.2 (Triazole-C), 117.8, 116.2, 114.6, 109.3, 108.7, 38.8 (N–CH_2_ carbon), 31.5, 28.3, 27.1, 26.4, 24.3, mass spectrum, *m*/*z* (*Irel*, %) *calcd*: *358 observed:* 359 [M + H]^+^.

##### 5-((1-(4-Nitrophenyl)-1*H*-1,2,3-triazol-4-yl)methyl)-5,6,7,8,9,10-hexahydrocyclohepta[*b*]indole (4r)

Yield: 87%; mp: 147–149 °C; IR (KBr, cm^−1^): 3105, 2930 (C–H), 1583, 1650 (C–NO_2_). ^1^H NMR (400 MHz, CDCl_3_) *δ* 8.36–8.31 (m, 2H, Ar–H), 7.87–7.81 (m, 2H, Ar–H), 7.57–7.49 (m, 2H, Ar–H), 7.30 (d, *J* = 7.3 Hz, 1H, Ar–H), 7.14 (m, *J* = 7.0, 1.2 Hz, 2H, Ar–H), 5.52 (s, 2H, N–CH_2_), 2.95–2.89 (m, 2H, aliphatic-H), 2.88–2.83 (m, 2H, aliphatic-H), 1.89 (dd, *J* = 7.3, 3.8 Hz, 2H, aliphatic-H), 1.79 (dd, *J* = 9.9, 4.8 Hz, 4H, aliphatic-H). ^13^C NMR (100 MHz, CDCl_3_) *δ* 147.4, 147.2, 140.9, 138.3, 135.2, 128.3, 125.4, 121.0, 120.4, 119.5, 119.4 (Triazole-C), 117.9, 114.9, 108.6, 38.7 (N–CH_2_ carbon), 31.4, 28.2, 27.1, 26.4, 24.3, mass spectrum, *m*/*z* (*Irel*, %) *calcd*: *387 observed:* 388 [M + H] + (100).

##### 9-((1-(4-Nitrophenyl)-1*H*-1,2,3-triazol-4-yl)methyl)-1,2,4,9-tetrahydrospiro[carbazole-3,2′-[1,3]dioxolane] (4s)

Yield: 95%; mp: 153–155 °C; IR (KBr, cm^−1^): 3145 (C–C), 2860 (C–H), 1601 (C–NO_2_), 1535. ^1^H NMR (400 MHz, CDCl_3_) *δ* 8.38–8.30 (m, 2H, Ar–H), 7.87–7.82 (m, 2H, Ar–H), 7.57 (s, 1H, Ar–H), 7.47 (d, *J* = 7.6 Hz, 1H, Ar–H), 7.30 (d, *J* = 8.0 Hz, 1H, Ar–H), 7.20–7.07 (m, 2H, Ar–H), 5.46 (s, 2H, N–CH_2_), 4.09–4.01 (m, 4H, aliphatic-H), 2.99 (d, *J* = 8.4 Hz, 3H, aliphatic-H), 2.17 (s, 1H), 2.11 (t, *J* = 6.6 Hz, 2H, aliphatic-H). ^13^C NMR (100 MHz, CDCl_3_) *δ* 147.2, 146.9, 140.9, 136.7, 133.4, 127.5, 125.4, 121.5, 120.4, 119.6, 119.5 (Triazole-C), 118.0, 108.9, 108.7, 108.5, 64.7, 38.8 (N–CH_2_ carbon), 32.0, 31.6, 20.5, mass spectrum, *m*/*z* (*Irel*, %) *calcd*: *431 observed:* 432 [M + H] + (100).

##### 4-(4-((3,4-Dihydro-1*H*-carbazol-9(2*H*)-yl)methyl)-1*H*-1,2,3-triazol-1-yl)benzoic acid (4t)

Yield: 90%; mp: 121–123 °C; IR (KBr, cm^−1^): 3146, 2750, 1759, 1600. ^1^H NMR (400 MHz, CDCl_3_) *δ* 10.98 (s, 1H, –OH proton), 8.19 (d, *J* = 8.7 Hz, 2H, Ar–H), 7.76 (d, *J* = 8.7 Hz, 2H, Ar–H), 7.55–7.50 (m, 2H, Ar–H), 7.33 (d, *J* = 8.0 Hz, 1H, Ar–H), 7.18–7.09 (m, 2H, Ar–H), 5.46 (s, 2H, N–CH_2_), 2.77 (dd, *J* = 14.3, 6.1 Hz, 4H, aliphatic-H), 1.91 (dd, *J* = 30.5, 5.7 Hz, 4H, aliphatic-H). ^13^C NMR (100 MHz, CDCl_3_) *δ* 169.3, 142.0, 136.9, 135.5, 131.8, 130.7, 130.6, 127.7, 123.3, 121.6, 119.9, 119.4, 118.4, 109.9, 108.5, 54.1, 23.1, 23.1, 23.8, 21.0, mass spectrum, *m*/*z* (*Irel*, %) *calcd*: *431 observed: 432* [M + H]^+^.

##### 5-((1-(4-(Trifluoromethyl)phenyl)-1*H*-1,2,3-triazol-4-yl)methyl)-5,6,7,8,9,10 hexahydrocyclohepta[*b*]indole (4u)

Yield: 72%; mp: 152–154 °C; IR (KBr, cm^−1^): 3031 (C–C), 2988, 1498, 1230 (C–F). ^1^H NMR (400 MHz, CDCl_3_) *δ* 7.91 (s, 1H), 7.81 (d, *J* = 8.0 Hz, 1H), 7.65 (d, *J* = 7.8 Hz, 1H, Ar–H), 7.59 (t, *J* = 7.9 Hz, 1H, Ar–H), 7.54–7.51 (m, 1H), 7.48 (s, 1H), 7.31 (d, *J* = 7.5 Hz, 1H), 7.18–7.09 (m, 2H, Ar–H), 5.51 (s, 2H, N–CH_2_), 2.96–2.90 (m, 2H, aliphatic-H), 2.88–2.82 (m, 2H, aliphatic-H), 1.89 (dd, *J* = 7.3, 3.7 Hz, 2H, aliphatic-H), 1.81–1.74 (m, 4H, aliphatic-H). ^13^C NMR (100 MHz, CDCl_3_) *δ* 147.0, 138.4, 137.1, 135.2, 132.5, 130.4, 128.3, 123.5, 120.9, 119.4, 119.3, 117.9, 114.8, 108.6, 117.4, 38.8, 31.5, 28.3, 27.1, 26.4, 24.3. Mass spectrum, *m*/*z* (*Irel*, %) *calcd*: *409 observed:* 410 [M + H]^+^.

### Antimicrobial assay

All the new N-substituted 1,2,3-triazolylmethyl indole derivatives were evaluated for antimicrobial activity against various bacterial and fungal cultures at a concentration of 10, 20 (μM) employing the well diffusion method.^35^ The standard antibiotic streptomycin was employed as standard. The well diffusion method was carried out on nutrient agar medium (0.5% peptone, 0.5% NaCl and 0.3% beef extract, HiMedia, Mumbai). The nutrient agar medium was autoclaved at 121 °C/15 lbs, after the autoclaving medium was cooled (40 °C) and poured into Petri dishes (Borosil, S-Line). After solidification, the Petri dishes were observed for sterility by overnight incubation. After the sterility check, the medium was cultured with 10^8^ cfu mL^−1^ actively grown (0.1 mL) bacterial culture using the spread plate technique. After a few minutes, wells were created on the cultured medium using a sterile well borer (5 mm) and selected dilutions of the synthesized N-substituted 1,2,3-triazolylmethyl indole derivatives with selected concentrations were inoculated. After inoculation, the cultured plates were incubated at 37 °C/24 h, and the antimicrobial activity was evaluated based on the zone of inhibition (mm) around the well.

### Antioxidant assay

The radical scavenging activity of the synthesized compounds was evaluated by using DPPH (2,2-diphenyl-1-picrylhydrazyl)^36^ and HRS (hydroxyl radical scavenging) methods.^37^ The reaction mixture was set with selected concentrations of synthesized compounds in 0.2 mM DPPH, and HRS mixture (1,10-phenanthroline: 2.5 mM, PBS: pH 7.4 and FeSO_4_: 2.5 mM) and incubated for 30 min in the dark and its absorbance recorded at 517 nm. Radical scavenging activity was evaluated using the following formula.DPPH activity (%) = (ABS_C_ − ABS_S_)/*S* × 100where ABS_C_ and ABS_S_ are the absorbance of the control and test samples, respectively. *S* is the volume (mL) of sample.HRS activity (%) = (*A*_s_ – *A*_c_)/(*A*_b_ – *A*_c_) × 100where *A*_s_ is the absorbance of the sample, *A*_c_ is the absorbance of the control (deionized water) and *A*_b_ is the absorbance of the solution without sample and H_2_O_2_.

### Anticancer activity assay

#### Maintenance and propagation of cell lines

The HeLa (cervical cancer) and MCF-7 (breast cancer) cell lines were procured from the National Center of Cell Sciences (NCCS), Pune. Both cell lines were maintained in Dulbecco's modified Eagle medium (DMEM-Gibco, USA) supplemented with antibiotic solution (100×) and 10% fetal calf serum (FCS-Gibco, USA). The cultured cells were propagated and maintained at 37 °C in a CO_2_ incubator containing 5% CO_2_ and 95% air. The cells were sub-cultured every 2 days by trypsinization for further experiments.

### MTT assay

The evaluation of the anti-cancer activity of the synthesized compounds with HeLa and MCF-7 cells was performed using the MTT assay.^38–40^ The cancer cells were seeded on 96-well tissue plates (Thermo Fisher Scientific, USA), where 1 × 10^5^ cells were seeded in each well. The plates were incubated in a 5% carbon dioxide incubator at 37 °C for 24 h, and post-seeding, the previous medium was replaced with 5 μM, 10 μM, 15 μM, 20 μM, and 25 μM of test compounds with DMEM in each well to achieve a total volume of 200 μL. The treated cells were incubated for 24 h. After a certain incubation period, the test compounds were discarded and 100 μL of 3-(4,5-dimethylthiazol-2-yl)-2,5-diphenyltetrazolium bromide (MTT) solution (5 mM mL^−1^ in MEM) was filled in each well and kept for 2–4 h in a 5% CO_2_ incubator in the dark. The formazan crystals formed by MTT was dissolved by adding 100 μL of dimethyl sulfoxide (DMSO) and incubated for another 20 min. The adsorption of the dissolved formazan crystal was measured using an ELISA reader (BioTek instrument, USA) at 570 nm. The experiments were carried out in triplicate. The anticancer activity of each tested compound was calculated as reported.^40^

### Molecular docking studies

In the present study, the AutoDock Vina PyRx virtual screening tool was used,^49^ which contributes a higher docking efficiency and accuracy. Autodock Vina uses a scoring function with efficient optimization and multithreading.^50^ The PyRx virtual screening tool is an open source (https://pyrx.sourceforge.io/) software downloaded and installed on a computer configured with Intel(R) Core (TM) i5-8250U CPU @1.60 GHz 1.80 GHz processor and RAM capacity of 8.00 GB. The ligand molecules were drawn using the ChemSketch (https://www.acdlabs.com) software tool and saved in MDL file (.mol) format, and converted to a PDB file using the Open Babel GUI tool.

To study the interactions between the newly synthesized ligand molecules and the target receptor, the crystal structure of the HeLa cell line protein caspase-3 (PDB id: 5IAE) and the crystal structure Human 17-beta-hydroxysteroid dehydrogenase type 1(PDB ID: 1FDW) (HBS) of the MCF-7 cell line were downloaded from the Protein Data Bank (https://www.rcsb.org). The proteins were prepared using the Biovia Discovery Studio software tool (https://discover.3ds.com/discovery-studio-visualizer-download). Initially, the water molecules were removed, and polar hydrogens added. The target protein was loaded in the PyRx tool and saved as a PBDQT file using the Autodock command. The ligands were loaded in PyRx using the Open Babel GUI input wizard. The energies of the ligands were minimized and converted to PDBQT file format. The Autodock Vina wizard was used to perform docking simulations after setting up a grid box in the active site pocket of the target molecule.

According to the docked procedure, the conformations were ranked according to their binding energy and the confirmation with lowest binding energy was considered the best docking score. The docking results were visualized using the Pymol, Biovia Discovery Studio Visualizer and Ligplot protocol.

## Conclusions

In conclusion, herein, we reported a simple and efficient method for the synthesis of novel *N*-substituted 1,2,3-triazolylmethyl indole derivatives (4a–u) employing the conventional method and microwave irradiation method. All the newly synthesized compounds were evaluated for antibacterial, antifungal, antioxidant and anticancer activities. The results of the antibacterial, antifungal and antioxidant and anticancer screening of all the synthesized compounds demonstrated that compounds 4n, 4p and 4s exhibited potent antibacterial and antifungal activity against selected microorganisms compared with the standard drug and moderate antioxidant activity. Compounds 4c, 4h, 4n, 4q and 4r significantly inhibited the proliferation more than 50% HeLa and MCF-7 cells in a dose-dependent manner for 24 h. Thus, the above-mentioned compounds can be considered as lead compounds for the further development of potent antimicrobial, antioxidant and anticancer drug agents. According to the docking studies, compounds 4c, 4h, and 4q have a higher affinity for the target cancer cells than the medication doxorubicin. Thus, the results of the docking investigations of the compounds and their biological activity are in good agreement.

## Author contributions

All the experiments were design and carried out by GT with the help of GS, DR, MS and BS. BKK carried out all biological activities and TV carried out docking studies. DA, Corresponding author designed the concept, monitored all experiments and prepared the manuscript. All authors read and approved the final manuscript.

## Conflicts of interest

The authors confirm that this article content has no conflict of interest.

## Supplementary Material

RA-013-D2RA05960F-s001
