# Supplementary material for: Microwave-assisted synthesis, molecular docking studies of 1,2,3-triazole-based carbazole derivatives as antimicrobial, antioxidant and anticancer agents
Source: RSC Adv. 2022 Dec 19;13(1):25–40. doi: 10.1039/d2ra05960f (PMC9761696; doi:10.1039/d2ra05960f)
Supplement: RA-013-D2RA05960F-s001 [file RA-013-D2RA05960F-s001.pdf]

Electronic Supplementary Material (ESI) for RSC Advances.
This journal is © The Royal Society of Chemistry 2022

SUPPLEMENTERY MATERIAL

Microwave-assisted synthesis, molecular docking studies of 1,2,3- Triazole based carbazole derivatives as antimicrobial, antioxidant, anticancer agents

Dongamanti Ashok[1]*, Gugulothu Thara[2], Bhukya Kiran Kumar[3], Gundu Srinivas[1], Dharavath Ravinder[1], Thumma Vishnu[4], Madderla Sarasija[5], Bujji Sushmitha[2]

[1]*Green and Medicinal Chemistry Laboratory, Department of Chemistry, Osmania University, Hyderabad-500007, Telangana, India.*

[2]*Department of Pharmacy, University College of Technology, Osmania University, Hyderabad -500007,Telangana, India.*

[3]*Department of Microbiology, University College of Science, Osmania University, Hyderabad -500007, Telangana, India.*

[4]*Matrusri Engineering College, Saidabad, Hyderabad, Telangana*

[5]*Department of Chemistry, Satavahana University, Karimnagar-505001, Telangana, India.*

Corresponding author mail id*:* ashokdou@gmail.com

1. Representation of synthesis of compound **1(a-d) …………** p2-p2
2. Spectral copies of the intermediates and target compounds **4(a-u**z….. p3-p8

Representation of synthesis of compound **1 &2(n)**

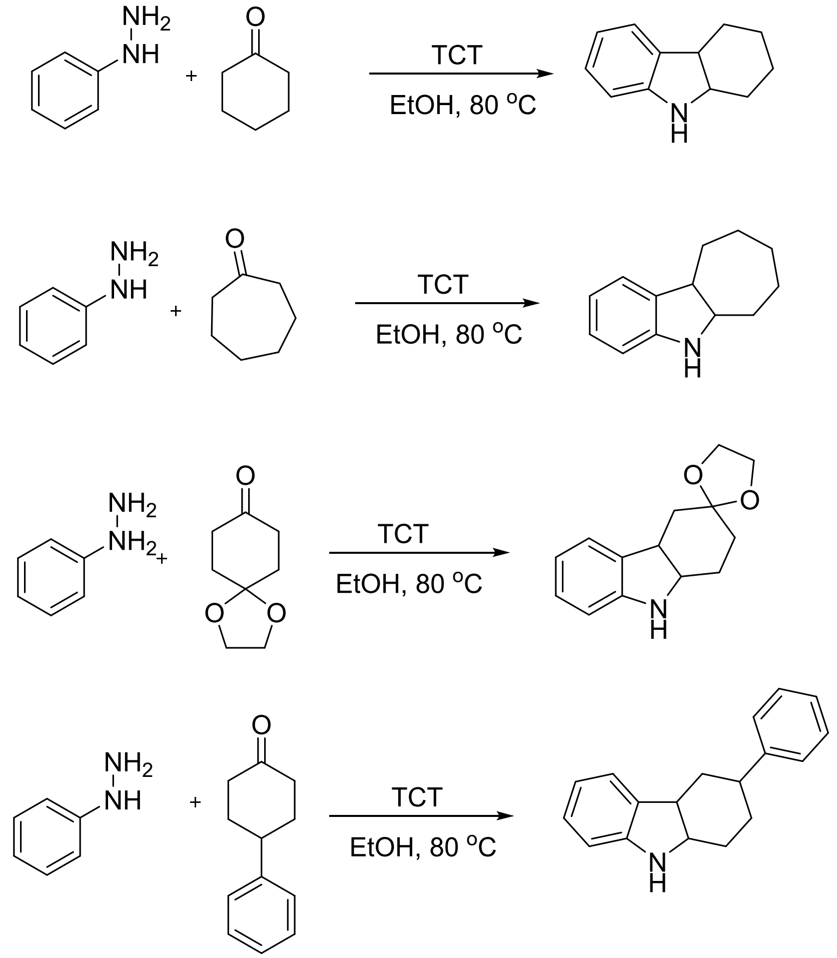

Copies of ^{1}H NMR, ^{13}C NMR, MASS and IR of synthesized compounds:

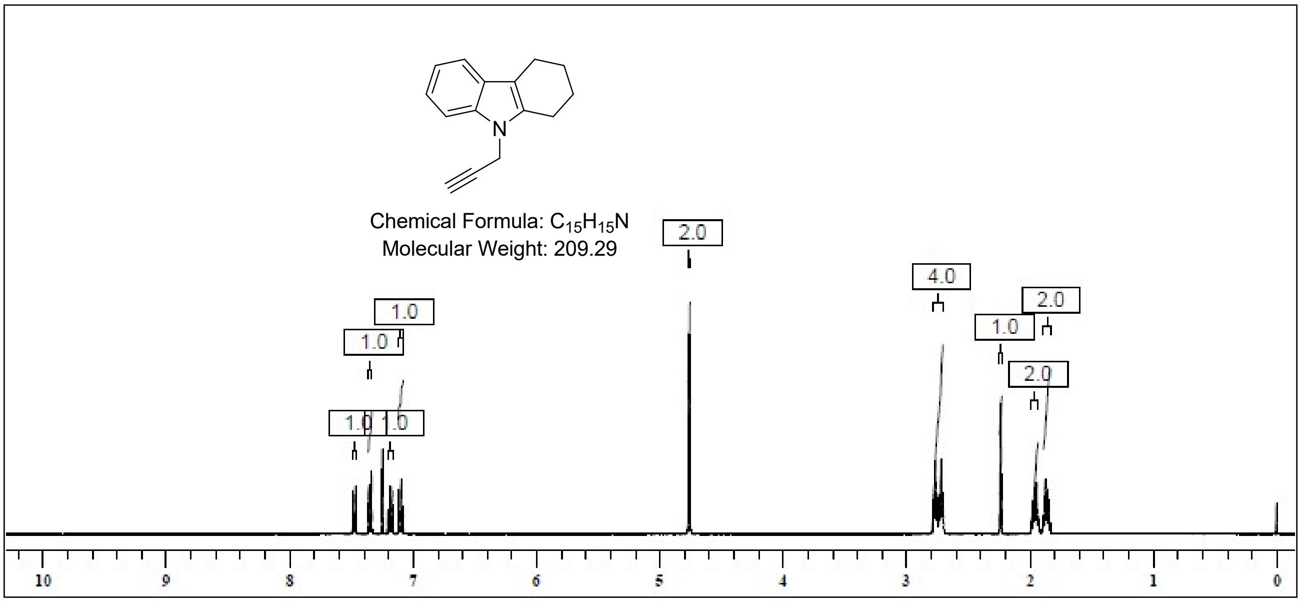

Figure S1: ^{1}H NMR (400 MHz, $CDCl_3$) spectrum of 9-(prop-2-yn-1-yl)-2,3,4,9-tetrahydro-1*H*-fluorene **2**

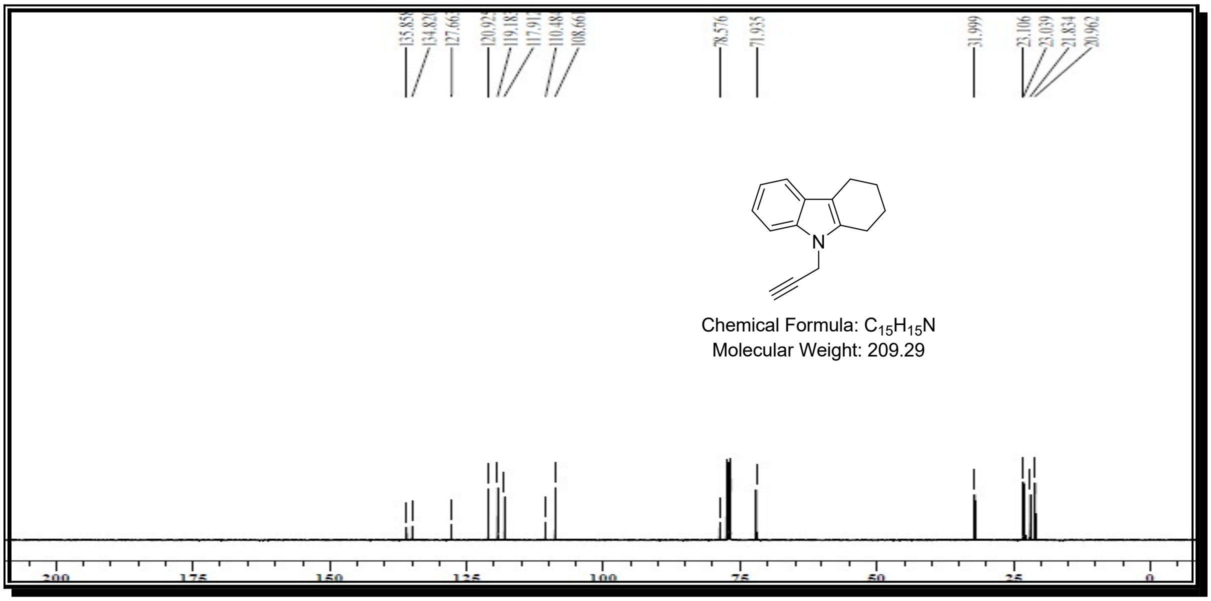

Figure S2: ^{13}C NMR (100 MHz, $CDCl_3$) spectrum of 9-(prop-2-yn-1-yl)-2,3,4,9-tetrahydro-1*H*-fluorene **2**

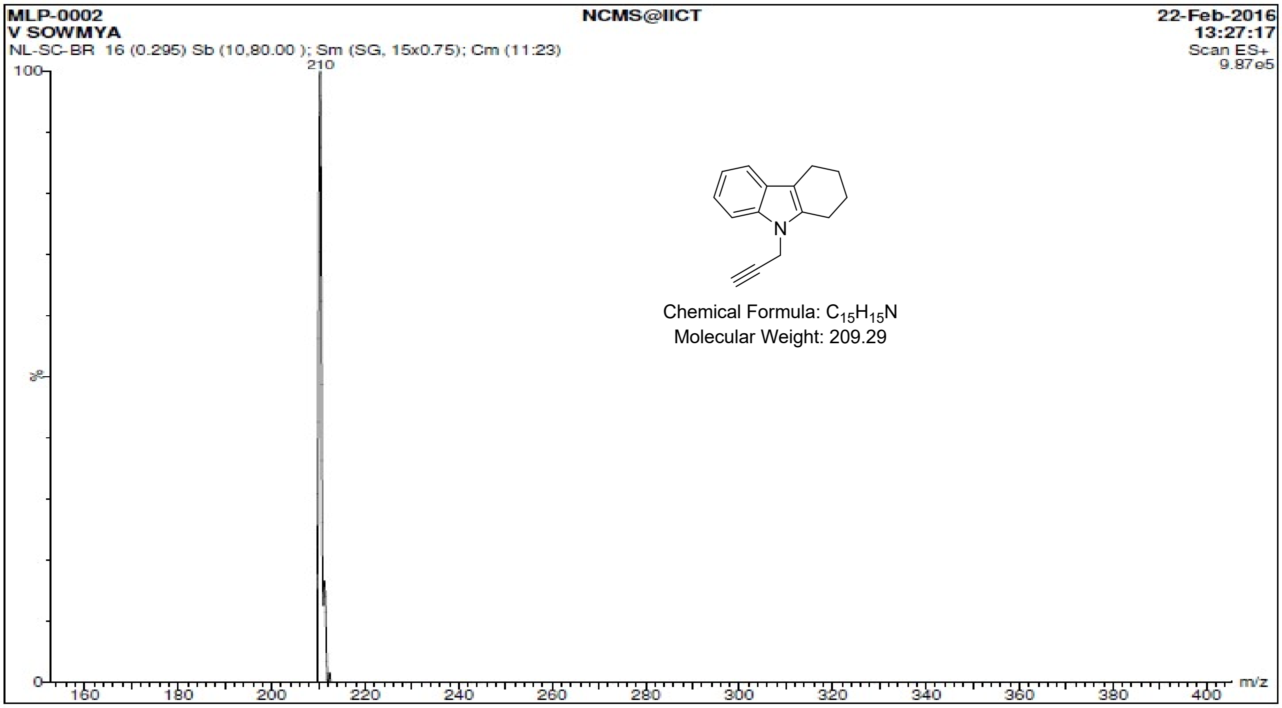

Figure S3: Mass (ES^+) spectrum of compound 9-(prop-2-yn-1-yl)-2,3,4,9-tetrahydro-1*H*-fluorene **2**

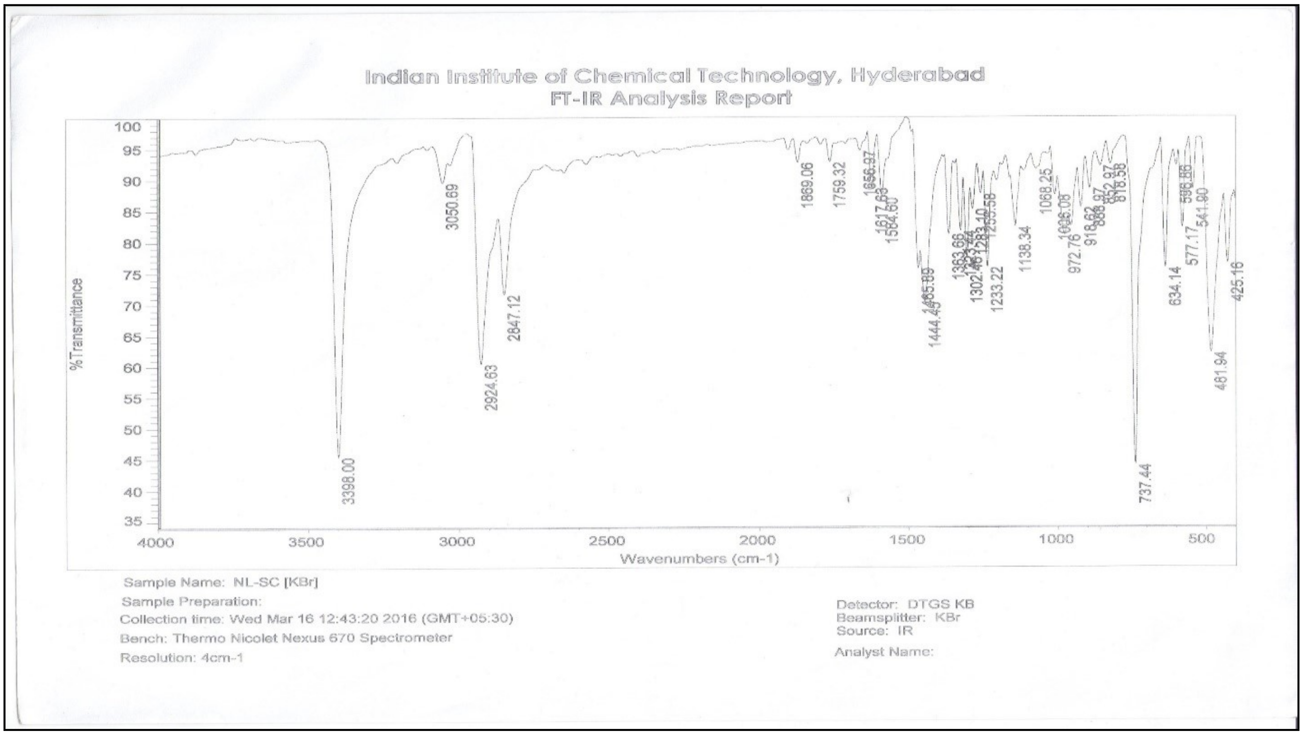

Figure S4: IR (KBr, cm^{-}) spectrum of compound 9-(prop-2-yn-1-yl)-2,3,4,9-tetrahydro-1*H*-fluorene **2**

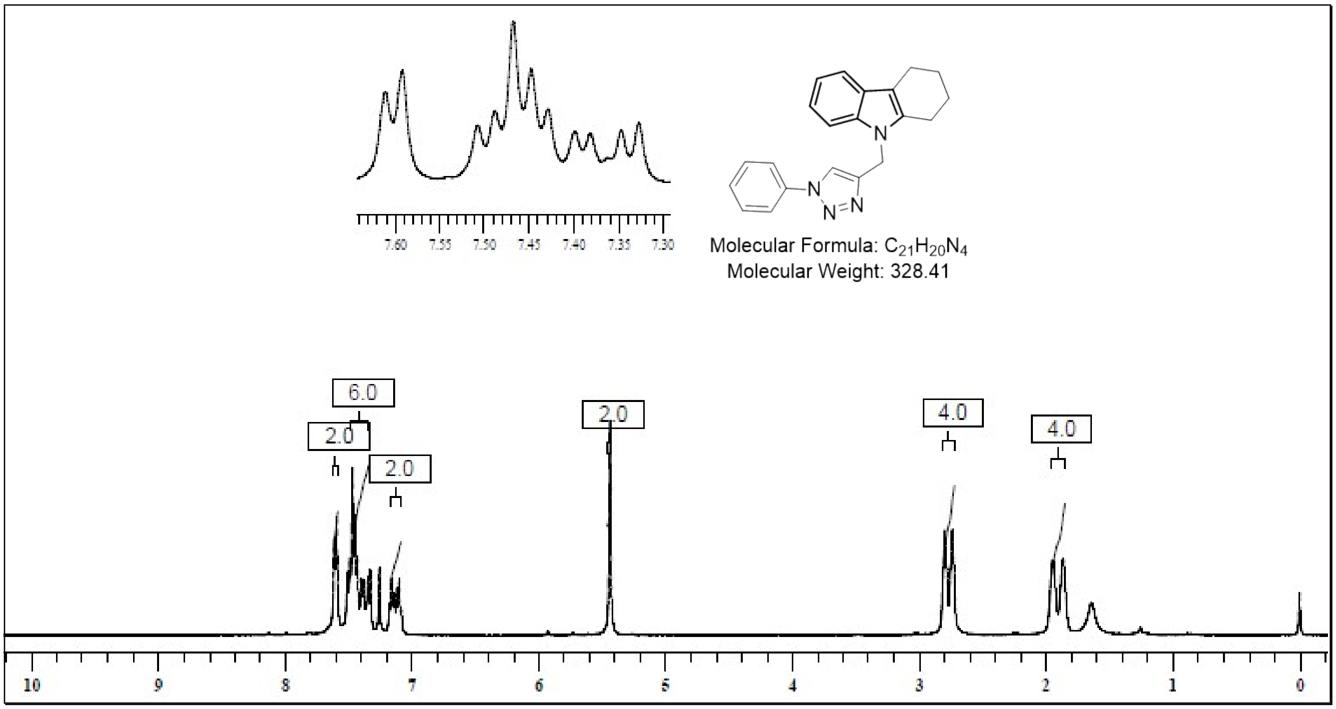

Figure S5: ^{1}H NMR (400 MHz, $CDCl_3$) spectrum of 9-((1-phenyl-1*H*-1,2,3-triazol-4-yl)methyl)-2,3,4,9-tetrahydro-1*H*-carbazole **(4a)**

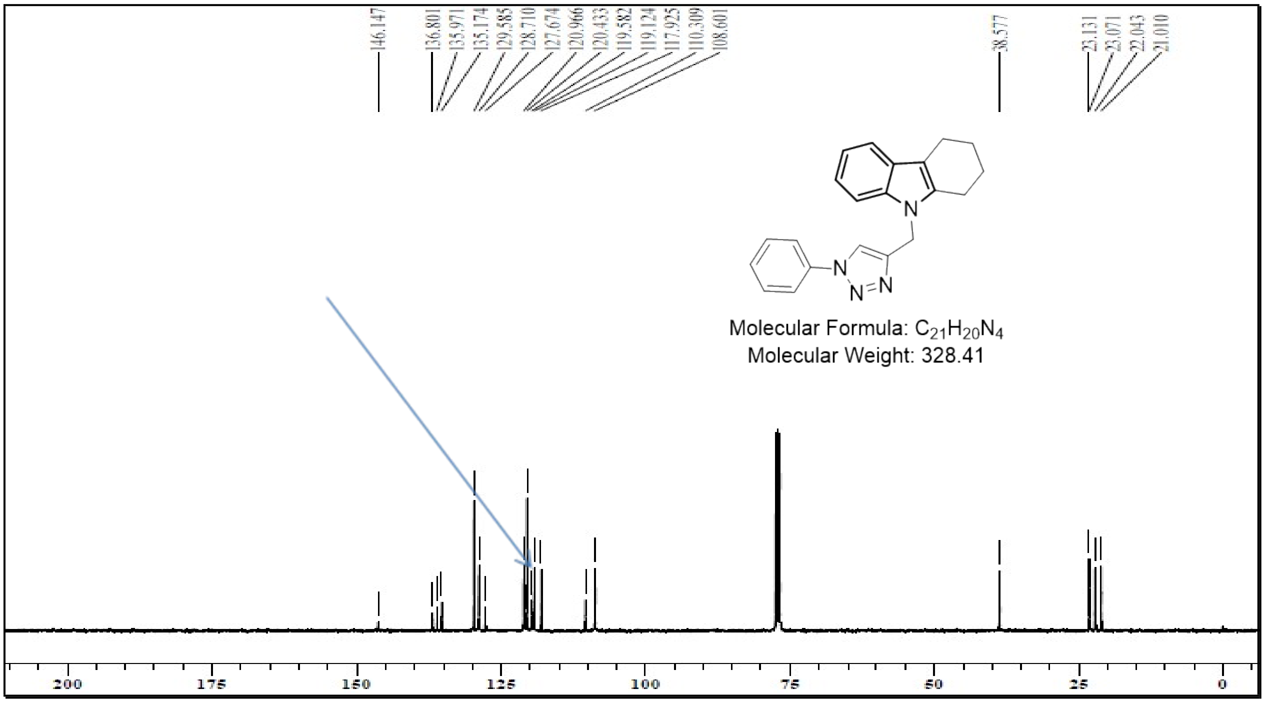

Figure S6: ^{13}C NMR (100 MHz, $CDCl_3$) spectrum of 9-((1-phenyl-1*H*-1,2,3-triazol-4-yl)methyl)-2,3,4,9-tetrahydro-1*H*-carbazole **(4a)**

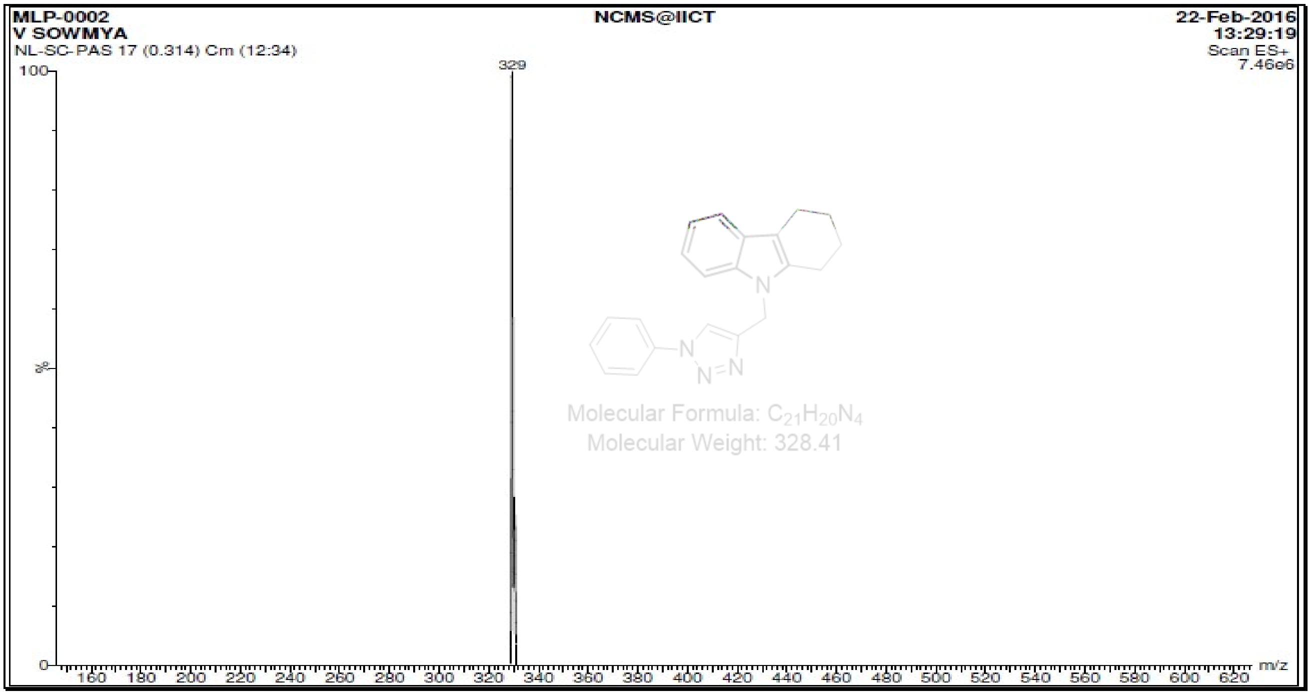

Figure S7: Mass (ES^+) spectrum of compound 9-((1-phenyl-1*H*-1,2,3-triazol-4-yl)methyl)-2,3,4,9-tetrahydro-1*H*-carbazole **(4a)**

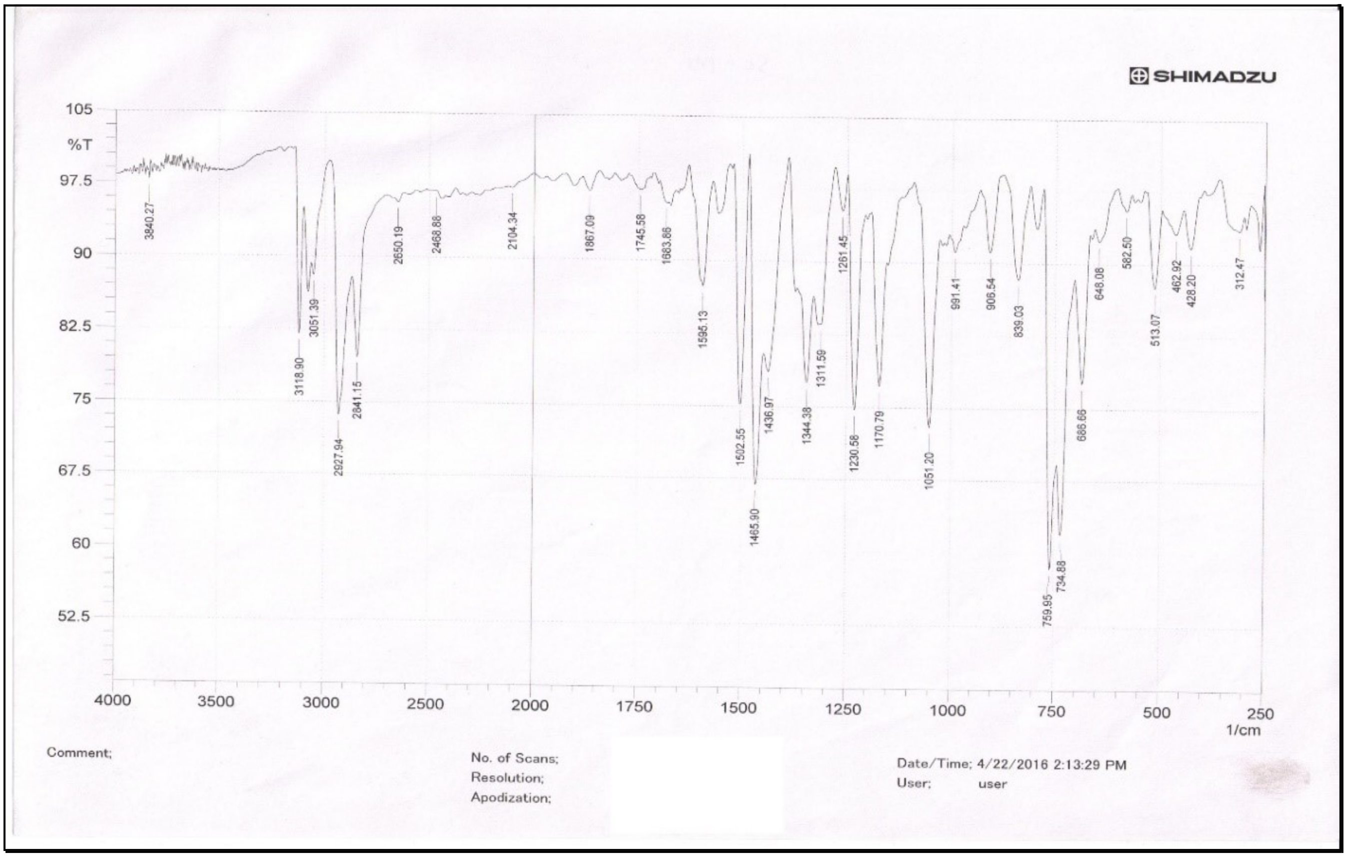

Figure S8: IR (KBr, cm^{-}) spectrum of compound 9-((1-phenyl-1*H*-1,2,3-triazol-4-yl)methyl)-2,3,4,9-tetrahydro-1*H*-carbazole **(4a)**

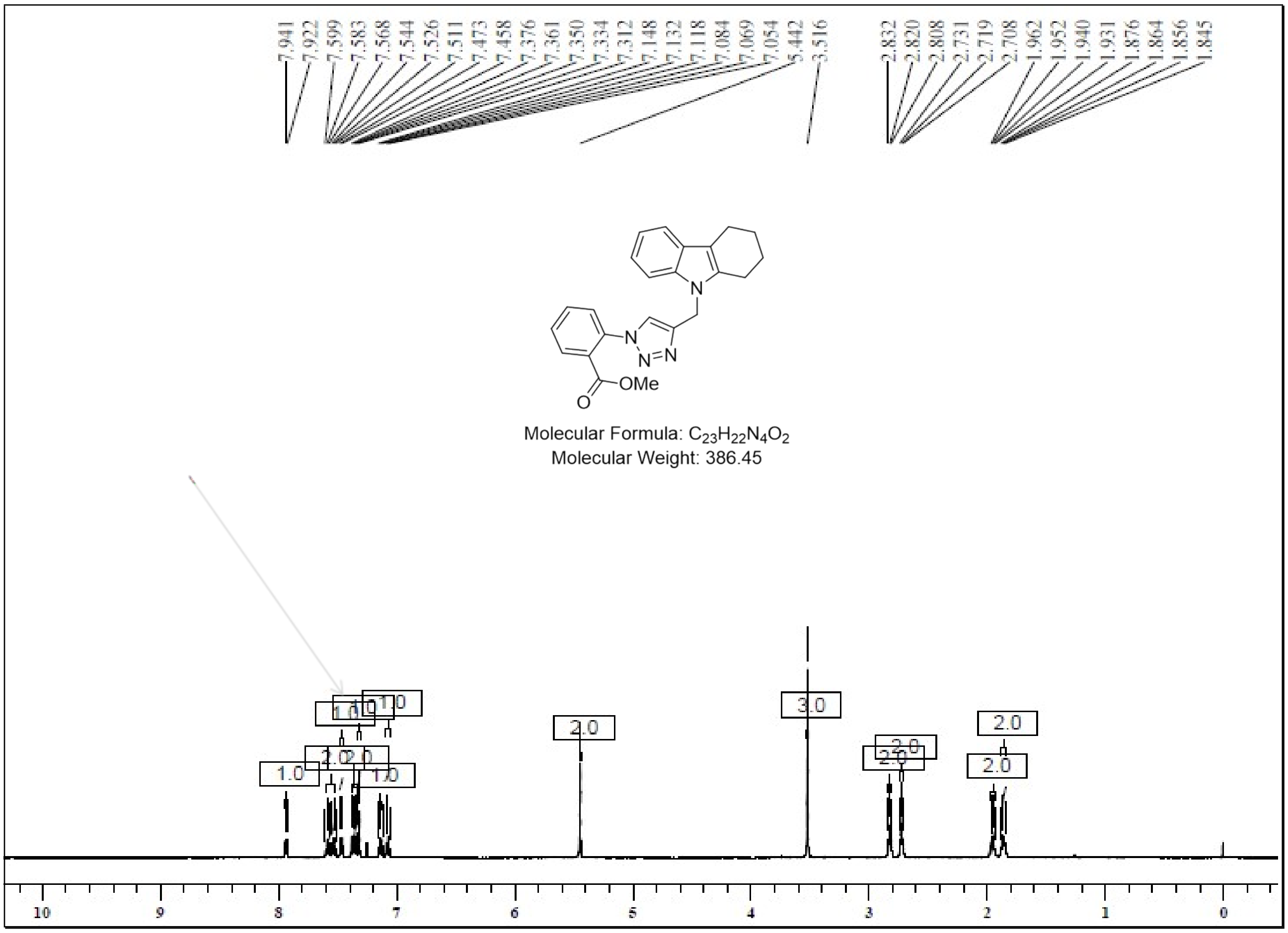

Figure S9: ^{1}H NMR (400 MHz, $CDCl_3$) of methyl 2-(4-((3,4-dihydro-1*H*-carbazol-9(2*H*)-yl)methyl)-1*H*-1,2,3-triazol-1-yl)benzoate **(4b)**

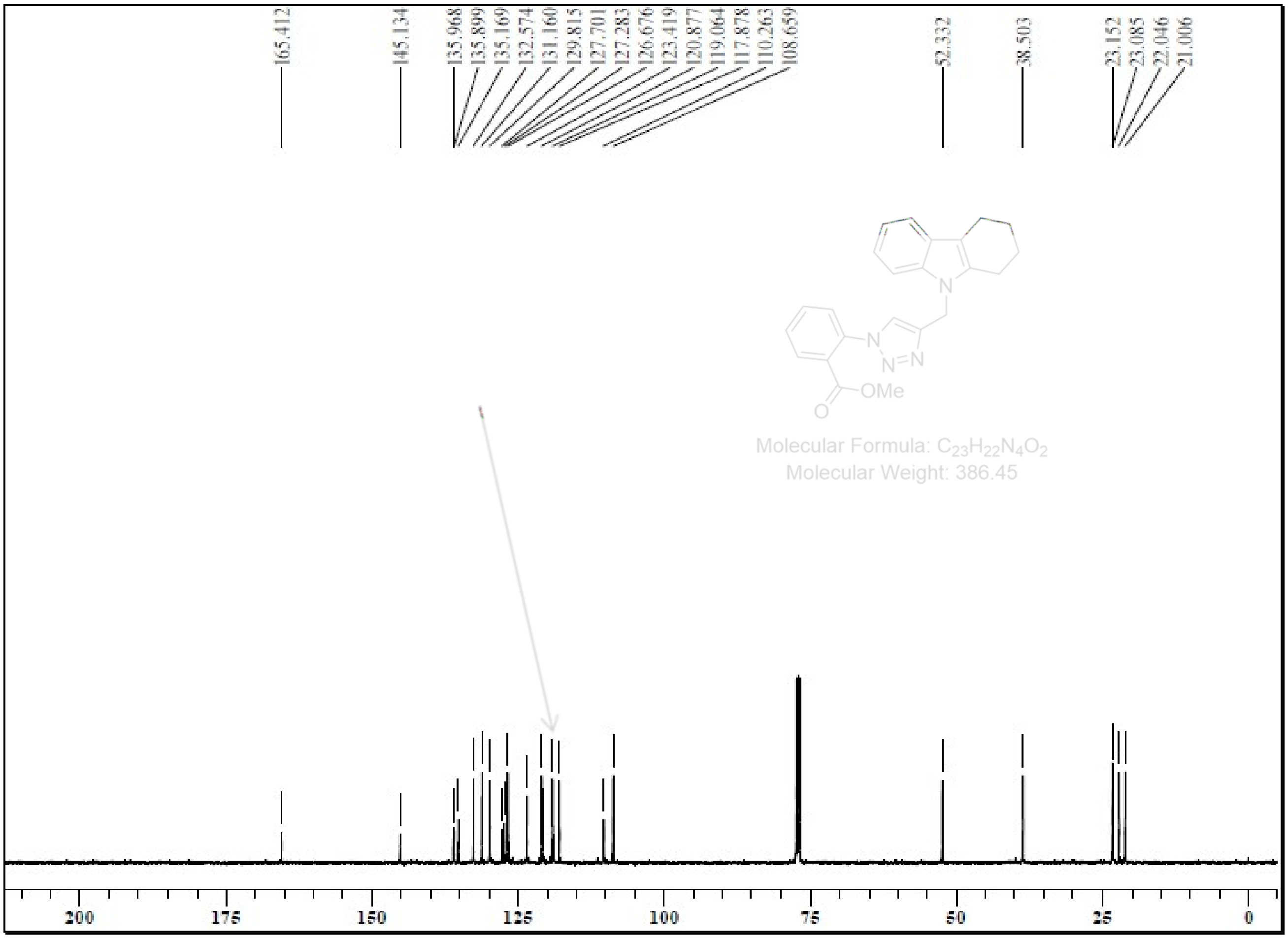

Figure S10: ^{13}C NMR (100 MHz, $CDCl_3$) spectrum of compound methyl 2-(4-((3,4-dihydro-1*H*-carbazol-9(2*H*)-yl)methyl)-1*H*-1,2,3-triazol-1-yl)benzoate **(4b)**

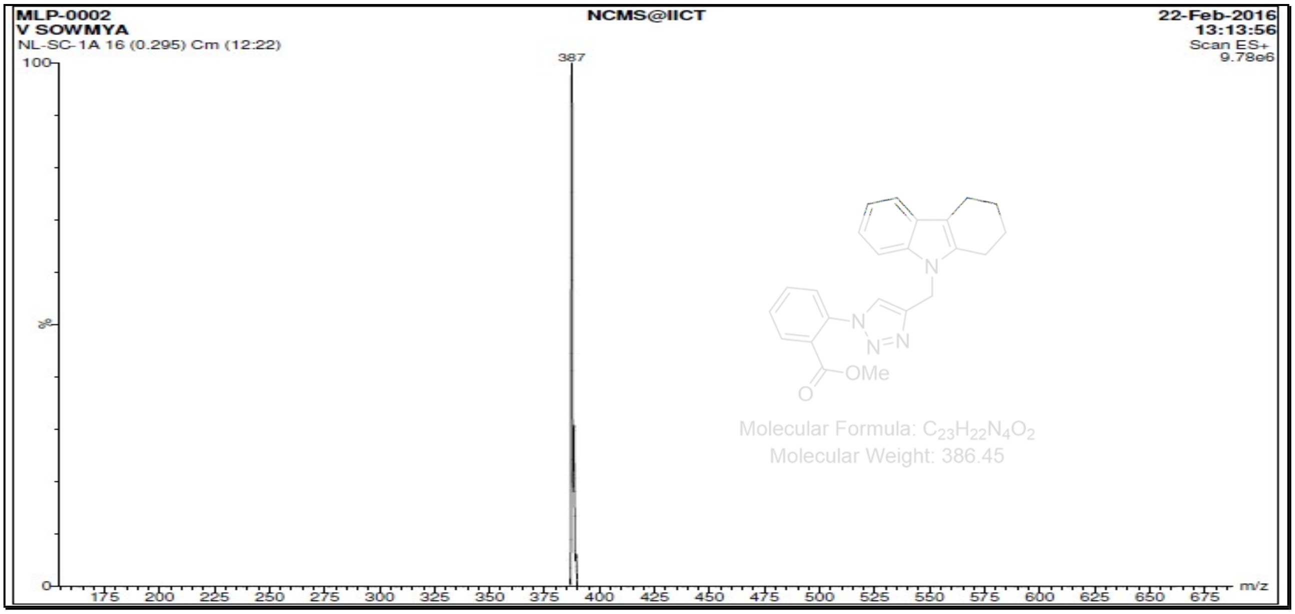

Figure S11: Mass (ES$^+$) spectrum of methyl 2-(4-((3,4-dihydro-1*H*-carbazol-9(2*H*)-yl)methyl)-1*H*-1,2,3-triazol-1-yl)benzoate **(4b)**

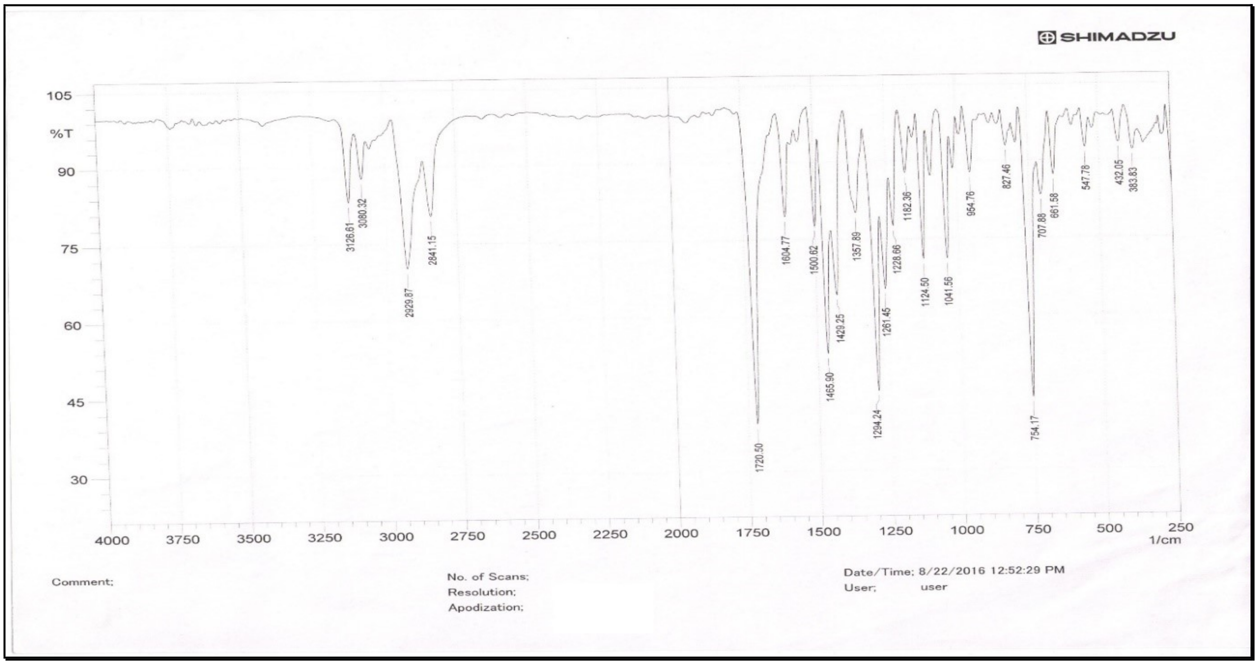

Figure S12: IR (KBr, cm$^-$) spectrum of methyl 2-(4-((3,4-dihydro-1*H*-carbazol-9(2*H*)-yl)methyl)-1*H*-1,2,3-triazol-1-yl)benzoate **(4b)**

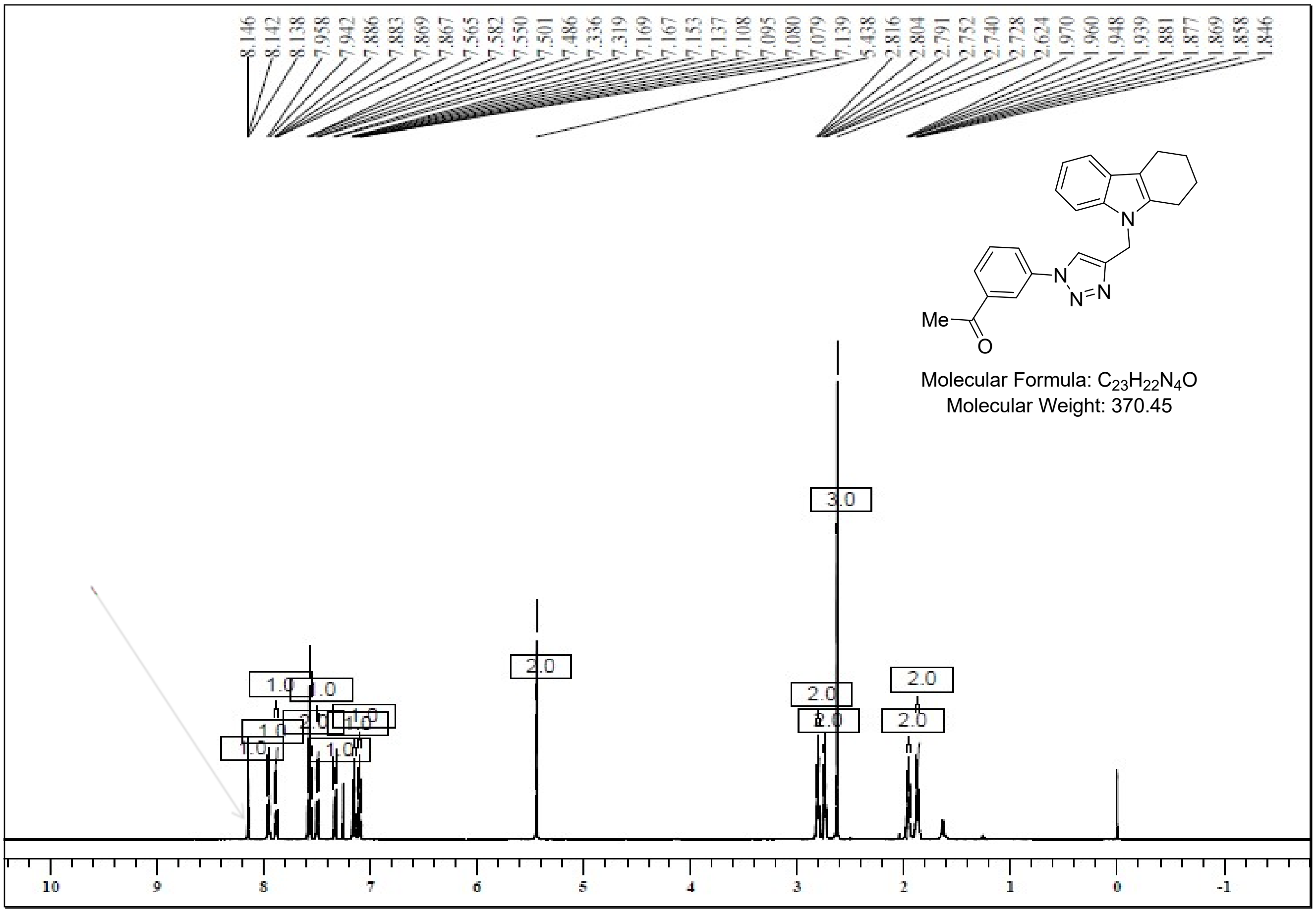

Figure S13: ^{1}H NMR (400 MHz, $CDCl_3$) of 1-(3-(4-((3,4-dihydro-1*H*-carbazol-9(2*H*)-yl)methyl)-1*H*-1,2,3-triazol-1-yl)phenyl)ethanone **(4c)**

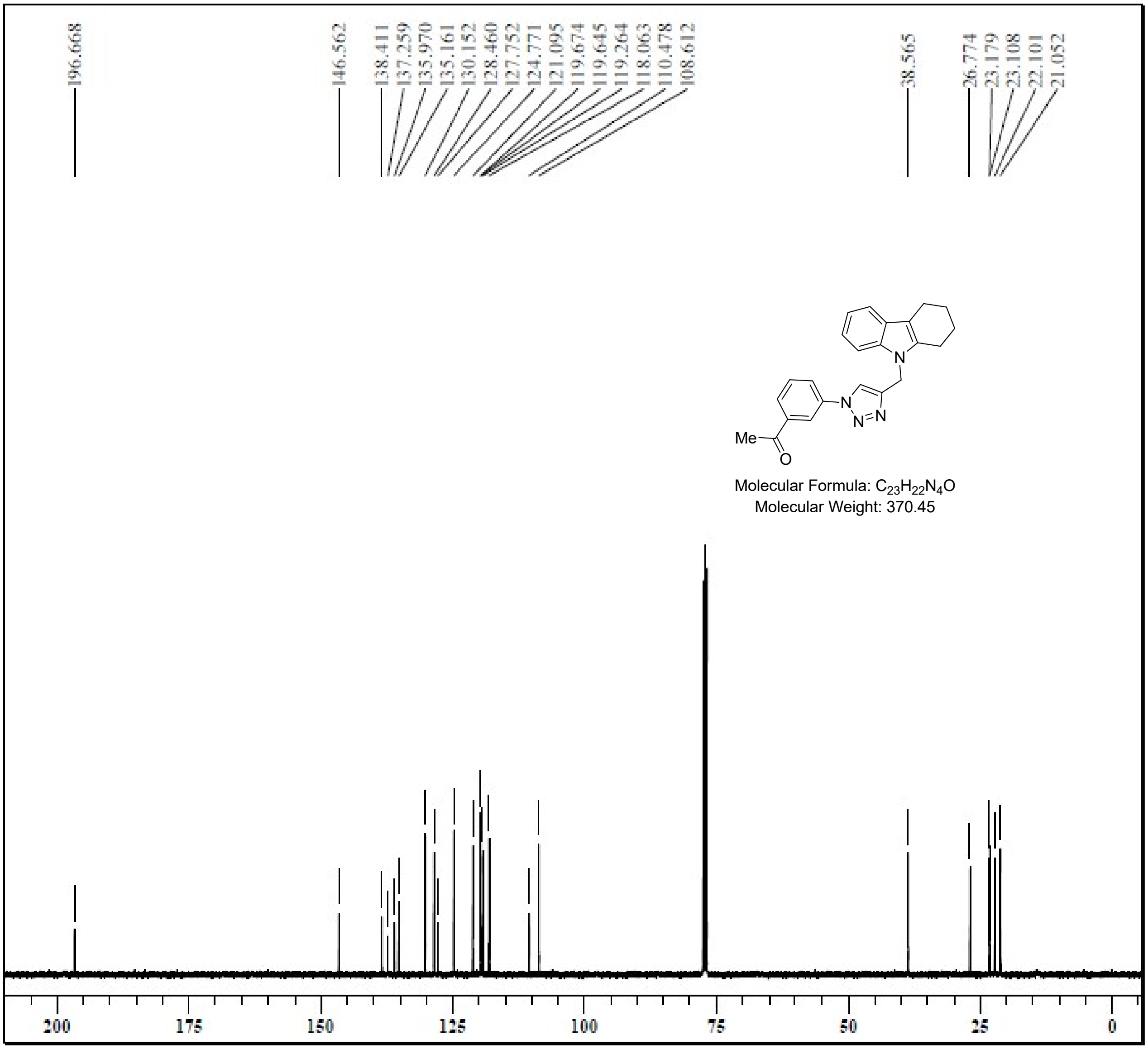

Figure S14: ^{13}C NMR (100 MHz, $CDCl_3$) spectrum of 1-(3-(4-((3,4-dihydro-1*H*-carbazol-9(2*H*)-yl)methyl)-1*H*-1,2,3-triazol-1-yl)phenyl)ethanone **(4c)**

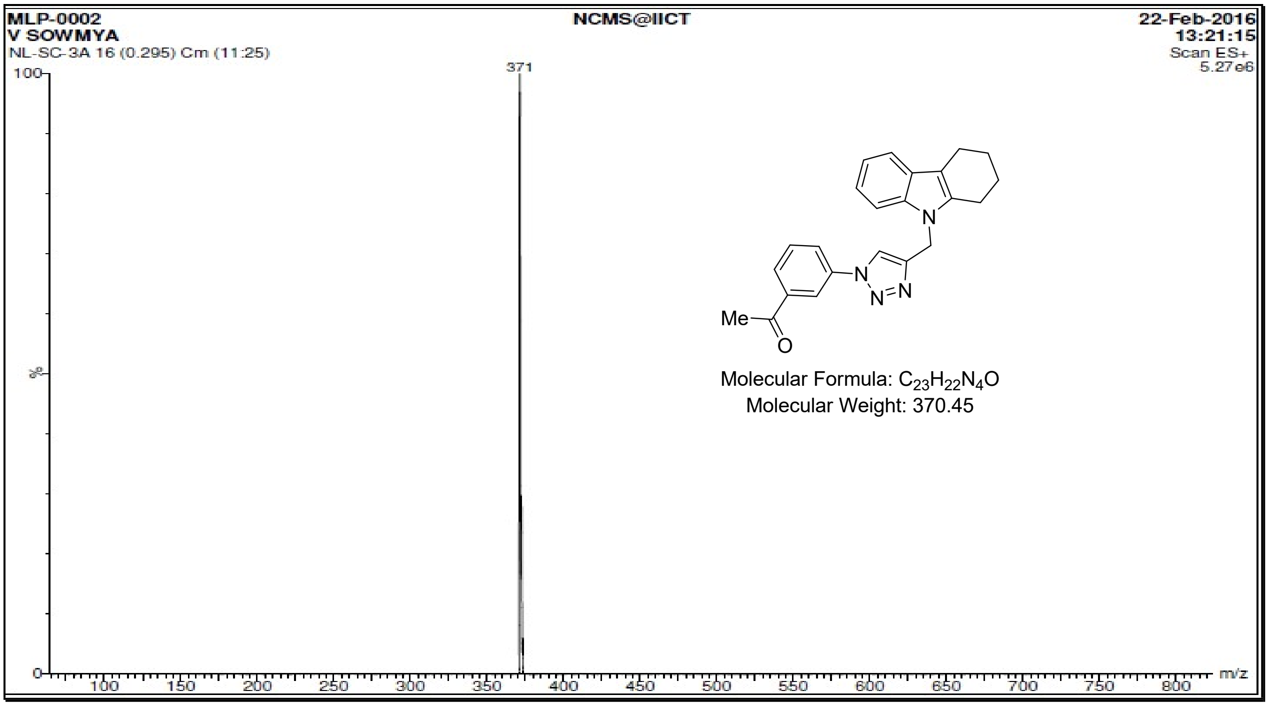

Figure S15: Mass (ES$^+$) spectrum of 1-(3-(4-((3,4-dihydro-1*H*-carbazol-9(2*H*)-yl)methyl)-1*H*-1,2,3-triazol-1-yl)phenyl)ethanone **(4c)**

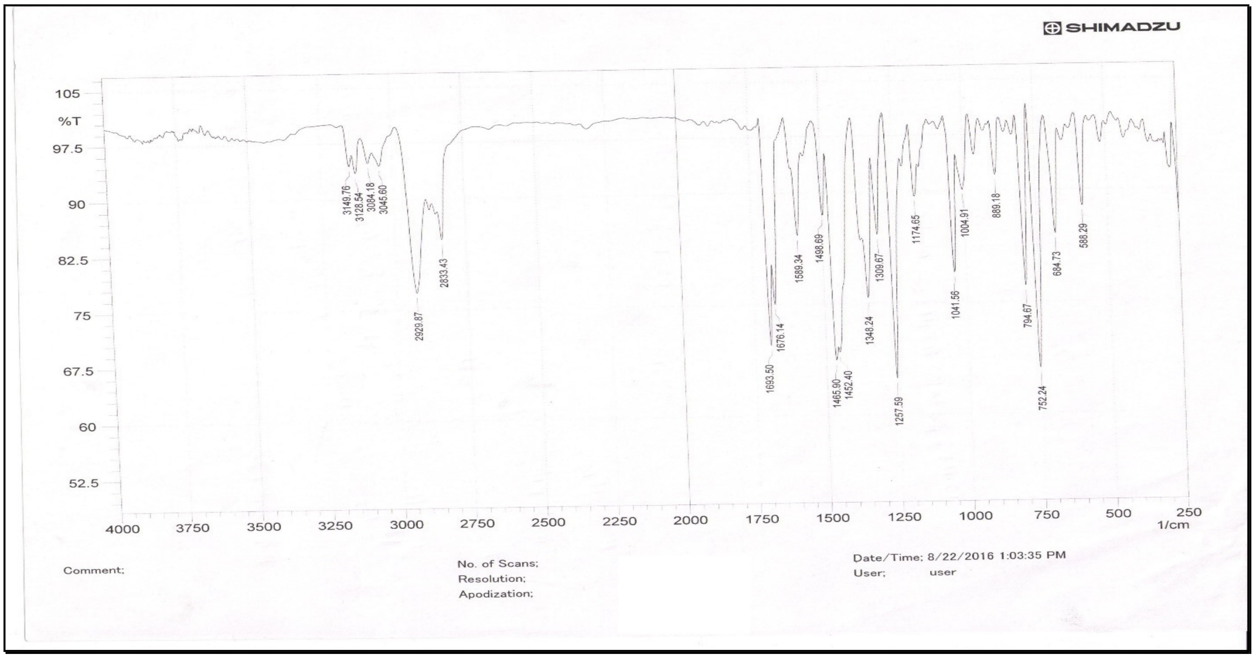

Figure S16: IR (KBr, cm$^-$) spectrum of 1-(3-(4-((3,4-dihydro-1*H*-carbazol-9(2*H*)-yl)methyl)-1*H*-1,2,3-triazol-1-yl)phenyl)ethanone **(4c)**

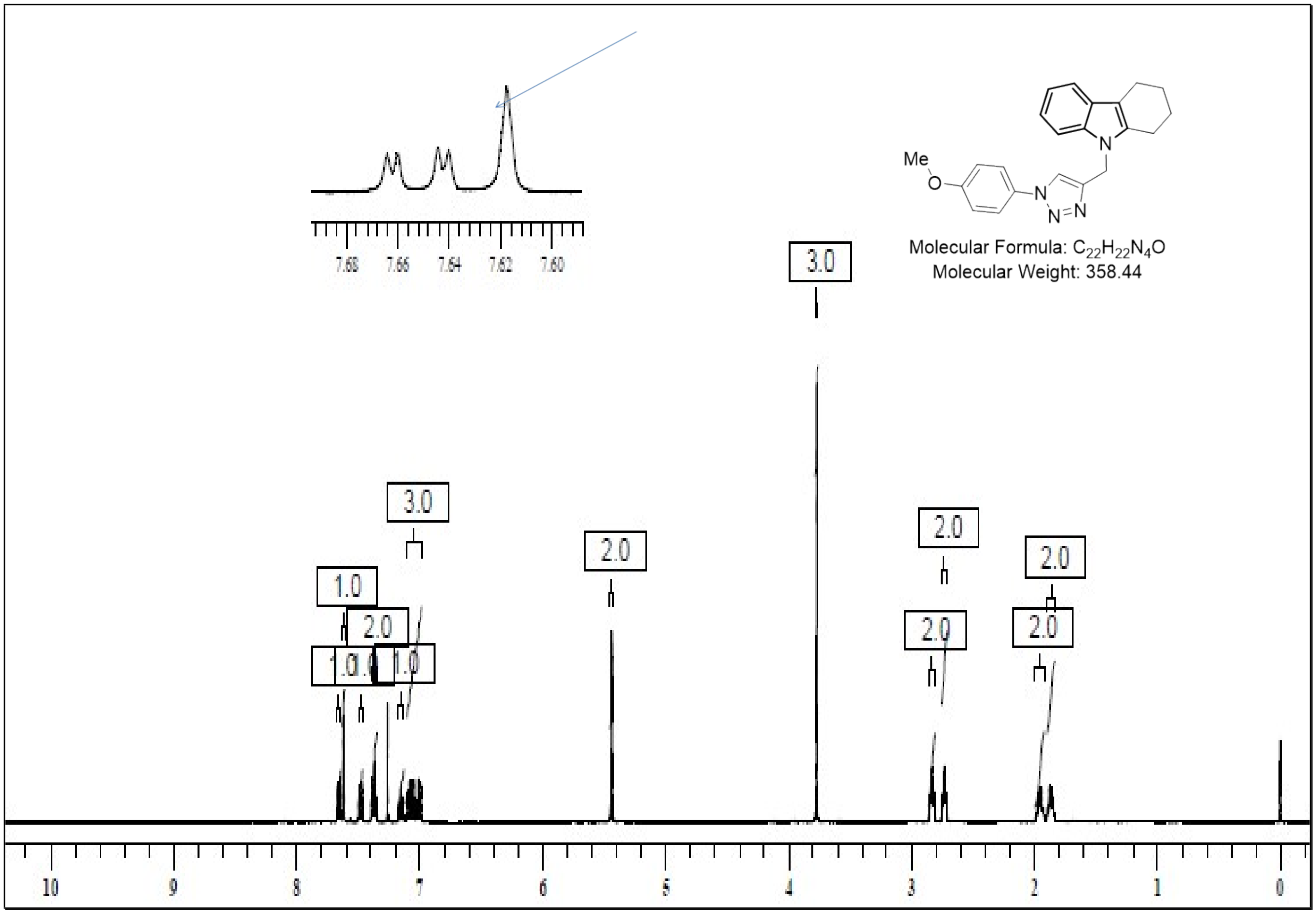

Figure S17: ^{1}H NMR (400 MHz, $CDCl_3$) of 9-((1-(4-methoxyphenyl)-1*H*-1,2,3-triazol-4-yl)methyl)-2,3,4,9-tetrahydro-1*H*-carbazole **(4d)**

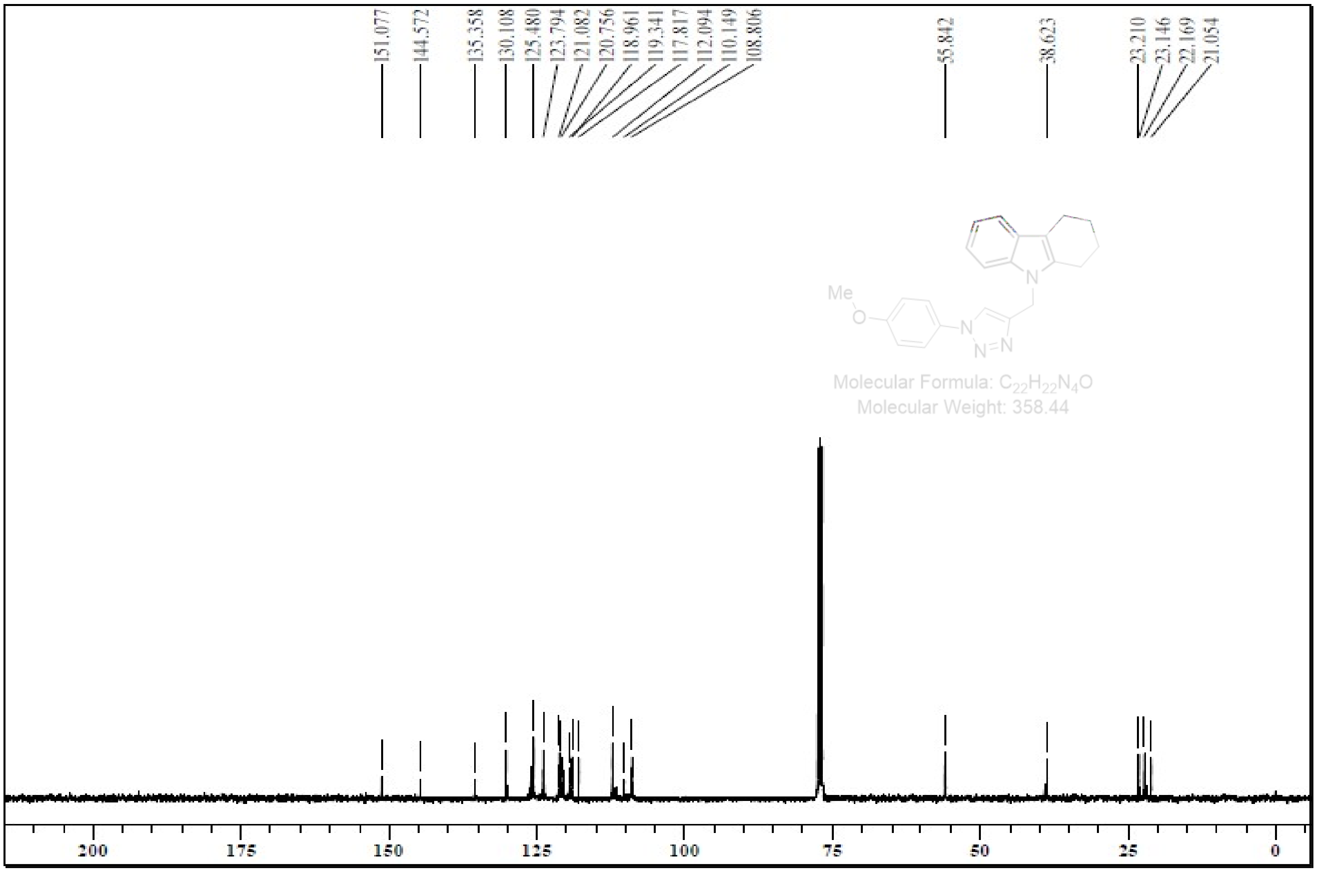

Figure S18: ^{13}C NMR (100 MHz, $CDCl_3$) spectrum of 9-((1-(4-methoxyphenyl)-1*H*-1,2,3-triazol-4-yl)methyl)-2,3,4,9-tetrahydro-1*H*-carbazole **(4d)**

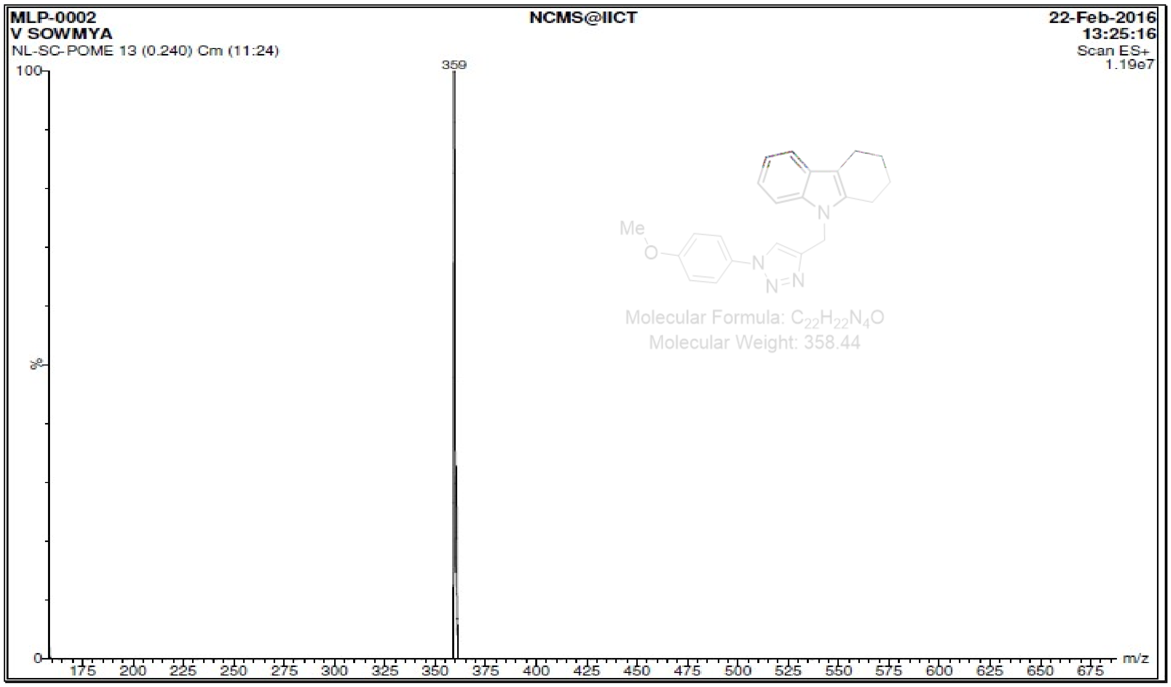

Figure S19: Mass (ES^+) spectrum of 9-((1-(4-methoxyphenyl)-1*H*-1,2,3-triazol-4-yl)methyl)-2,3,4,9-tetrahydro-1*H*-carbazole **(4d)**

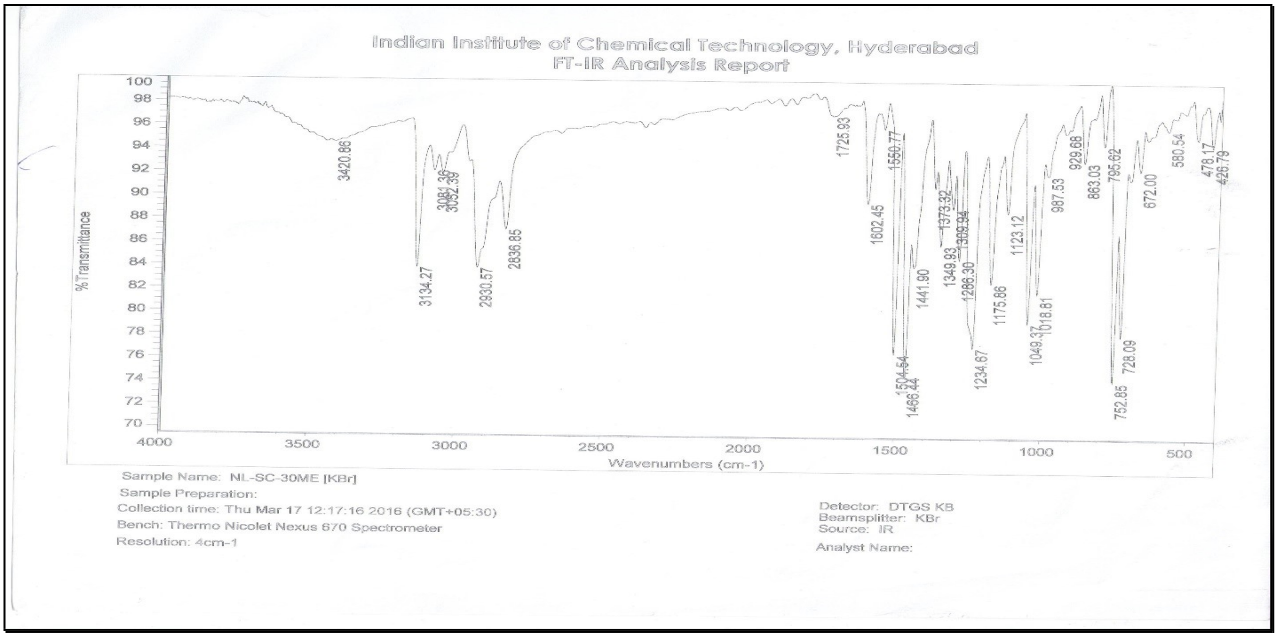

Figure S20: IR (KBr, cm^-) spectrum of 9-((1-(4-methoxyphenyl)-1*H*-1,2,3-triazol-4-yl)methyl)-2,3,4,9-tetrahydro-1*H*-carbazole **(4d)**

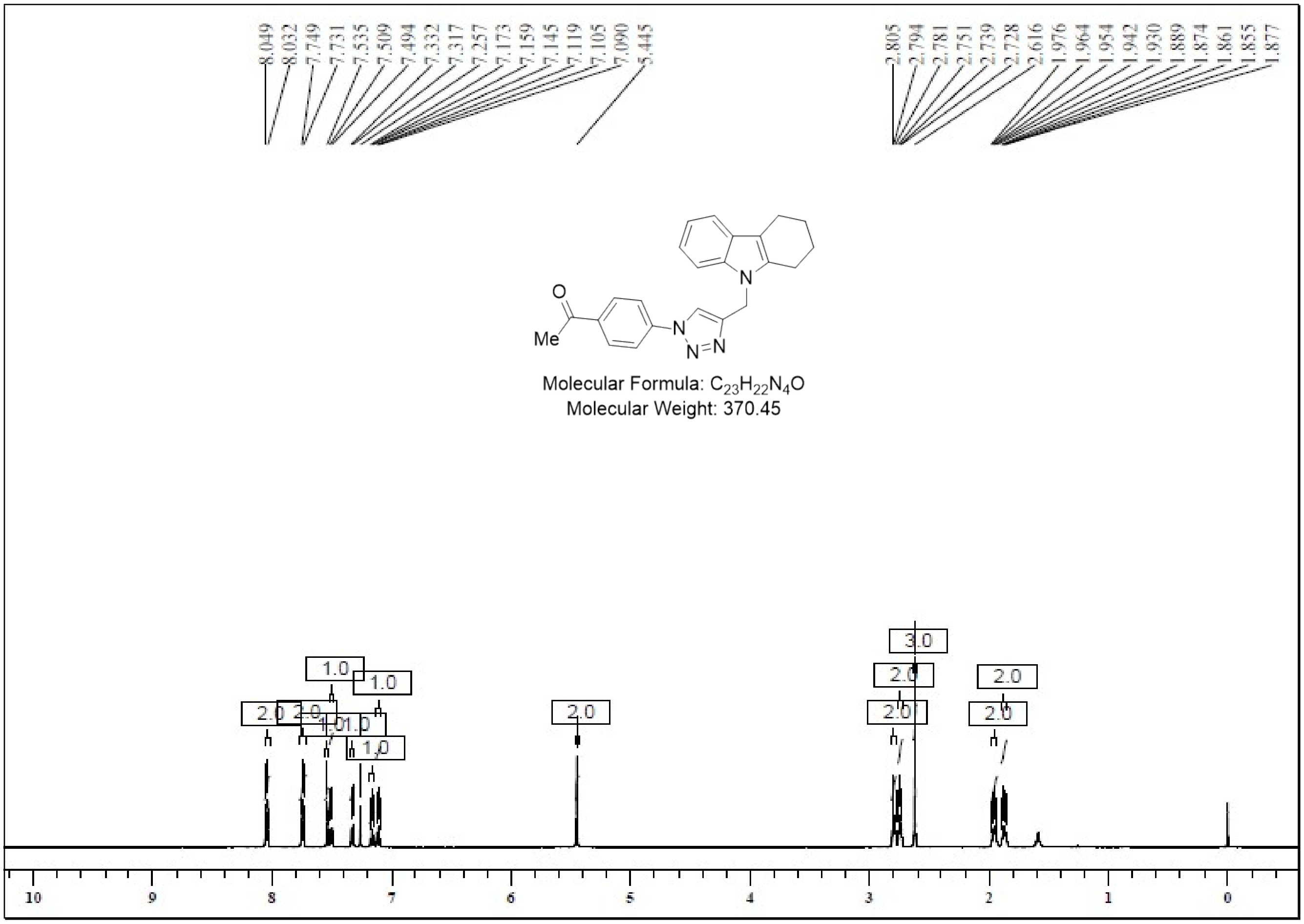

Figure S21: ^{1}H NMR (400 MHz, $CDCl_3$) of 1-(4-(4-((3,4-dihydro-1*H*-carbazol-9(2*H*)-yl)methyl)-1*H*-1,2,3-triazol-1-yl)phenyl)ethanone **(4e)**

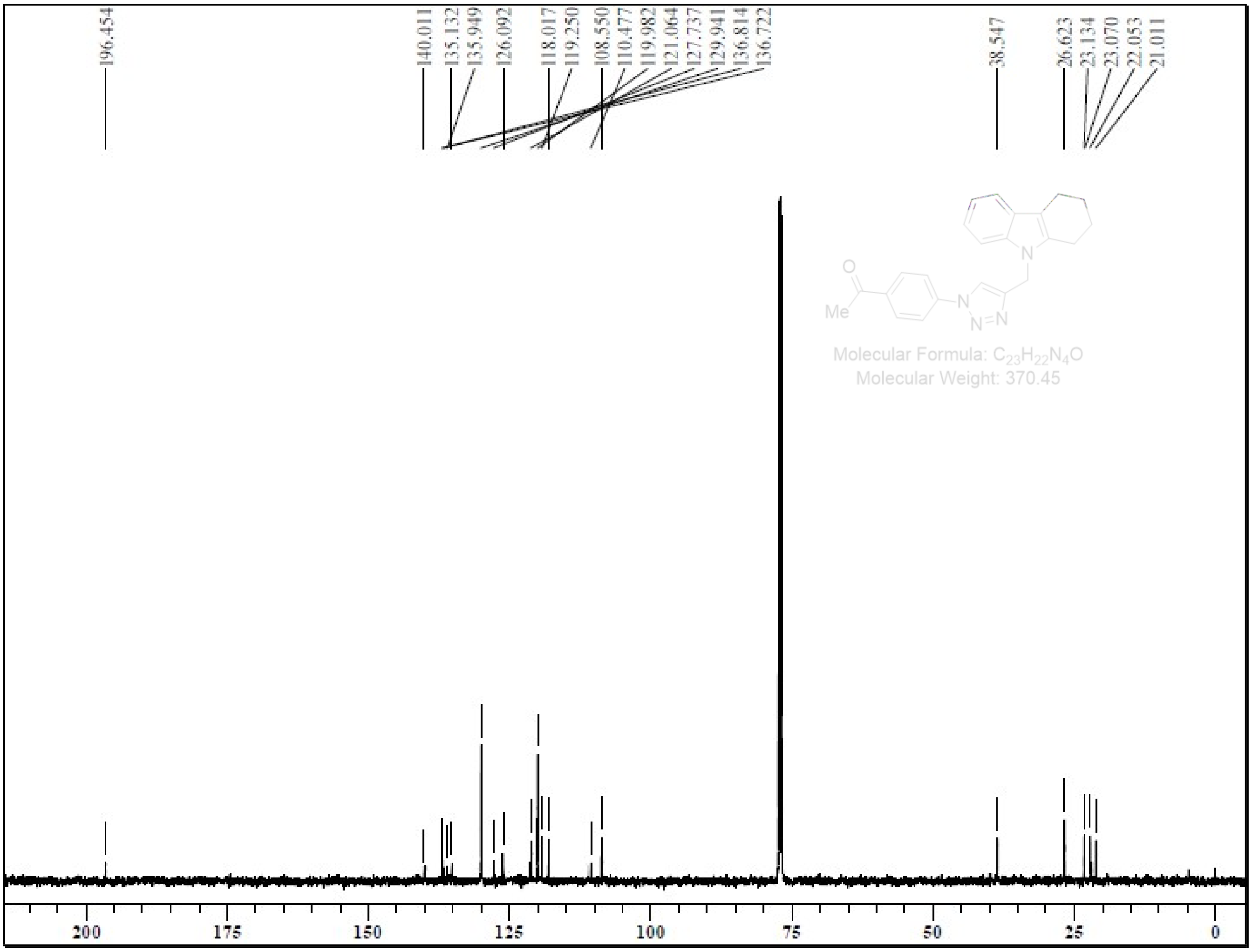

Figure S22: ^{13}C NMR (100 MHz, $CDCl_3$) spectrum of 1-(4-(4-((3,4-dihydro-1*H*-carbazol-9(2*H*)-yl)methyl)-1*H*-1,2,3-triazol-1-yl)phenyl)ethanone **(4e)**

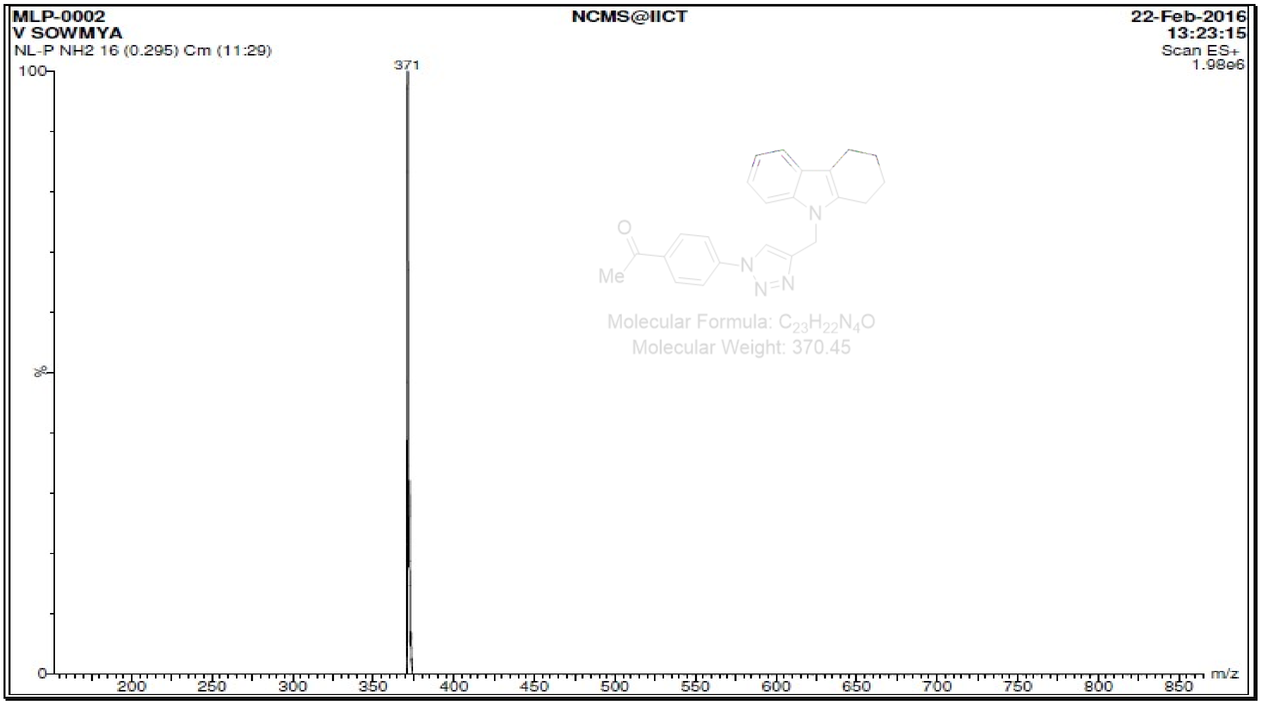

Figure S23: Mass (ES^+) spectrum of 1-(4-(4-((3,4-dihydro-1*H*-carbazol-9(2*H*)-yl)methyl)-1*H*-1,2,3-triazol-1-yl)phenyl)ethanone **(4e)**

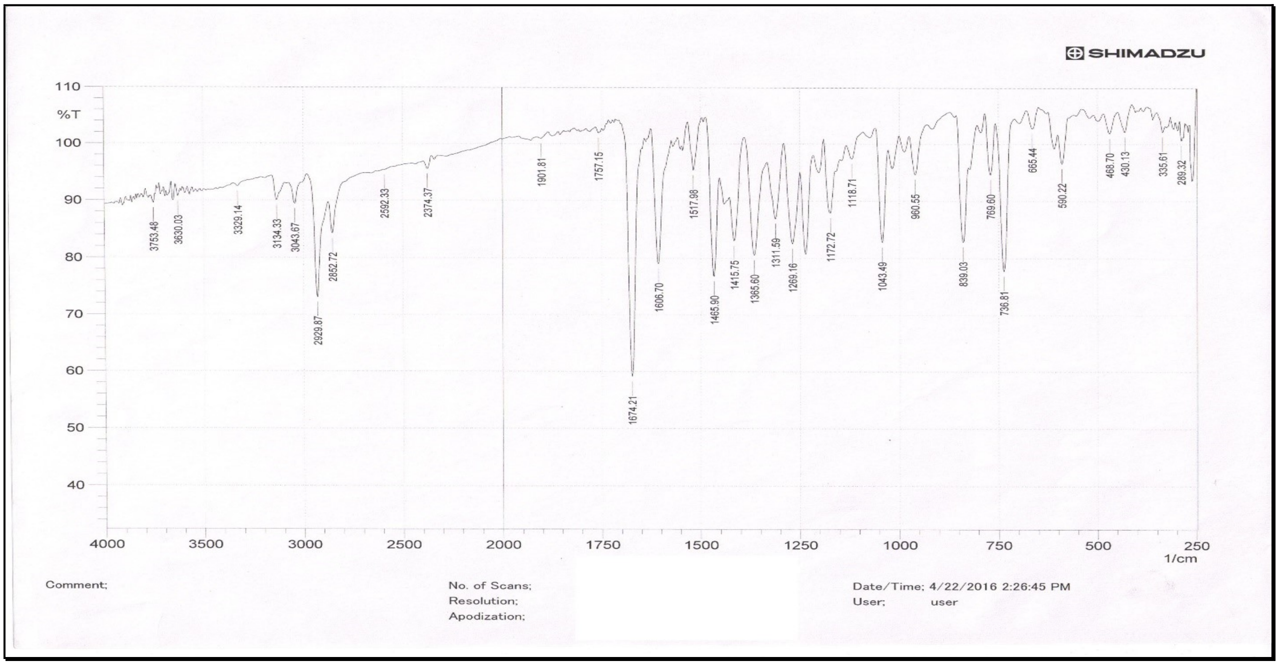

Figure S24: IR (KBr, cm^-) spectrum of 1-(4-(4-((3,4-dihydro-1*H*-carbazol-9(2H)-yl)methyl)-1*H*-1,2,3-triazol-1-yl)phenyl)ethanone **(4e)**

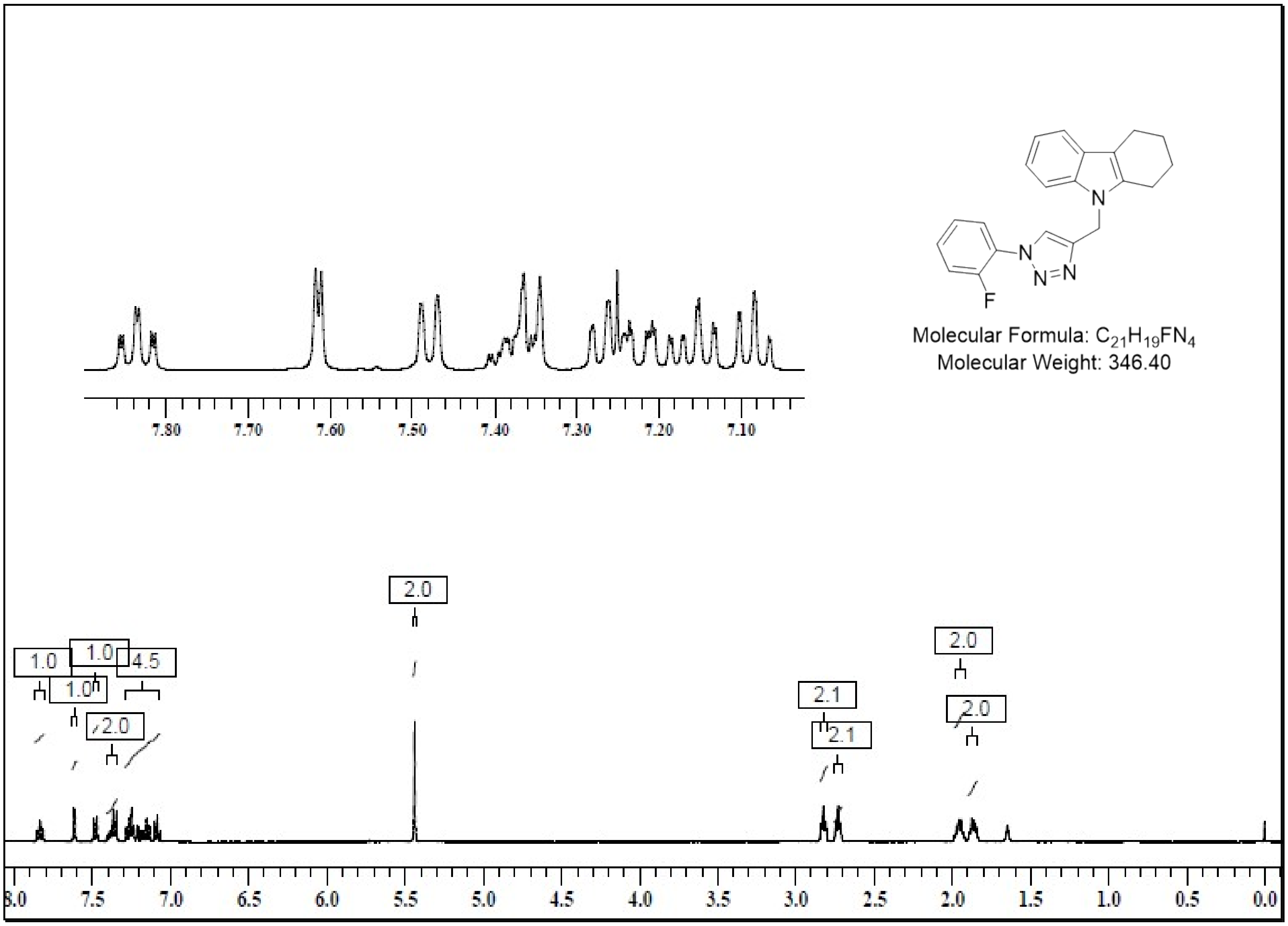

Figure S25: ^1H NMR (400 MHz, $CDCl_3$) of 9-((1-(2-fluorophenyl)-1H-1,2,3-triazol-4-yl)methyl)-2,3,4,9-tetrahydro-1H-carbazole **(4f)**

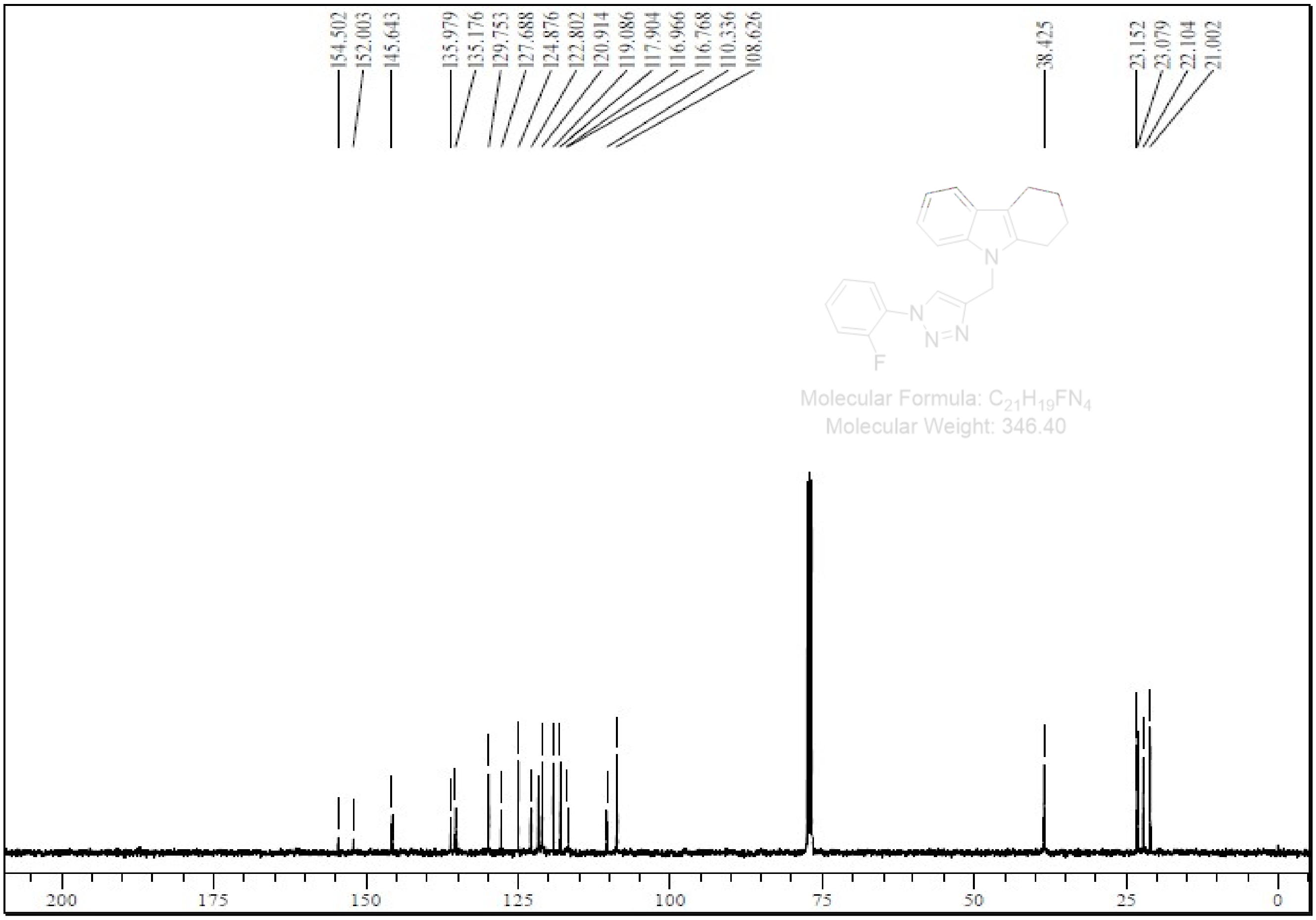

Figure S26: ^{13}C NMR (100 MHz, $CDCl_3$) spectrum of 9-((1-(2-fluorophenyl)-1H-1,2,3-triazol-4-yl)methyl)-2,3,4,9-tetrahydro-1H-carbazole **(4f)**

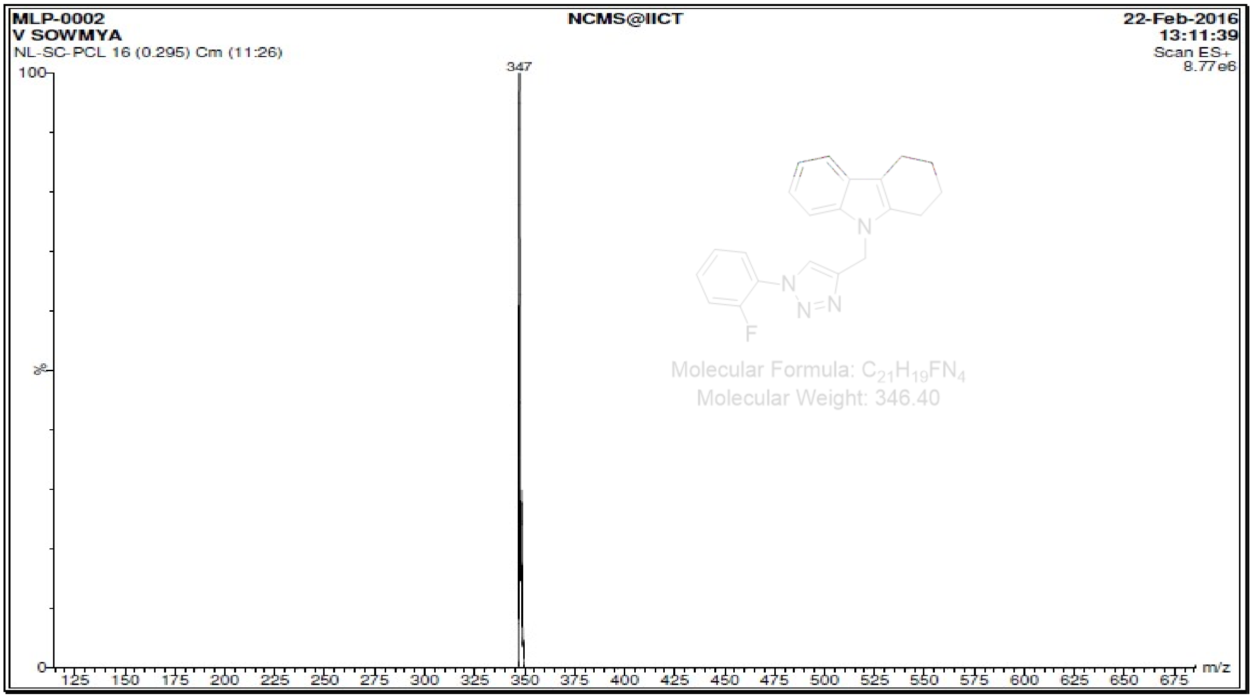

Figure S27: Mass (ES^+) spectrum of 9-((1-(2-fluorophenyl)-1H-1,2,3-triazol-4-yl)methyl)-2,3,4,9-tetrahydro-1H-carbazole **(4f)**

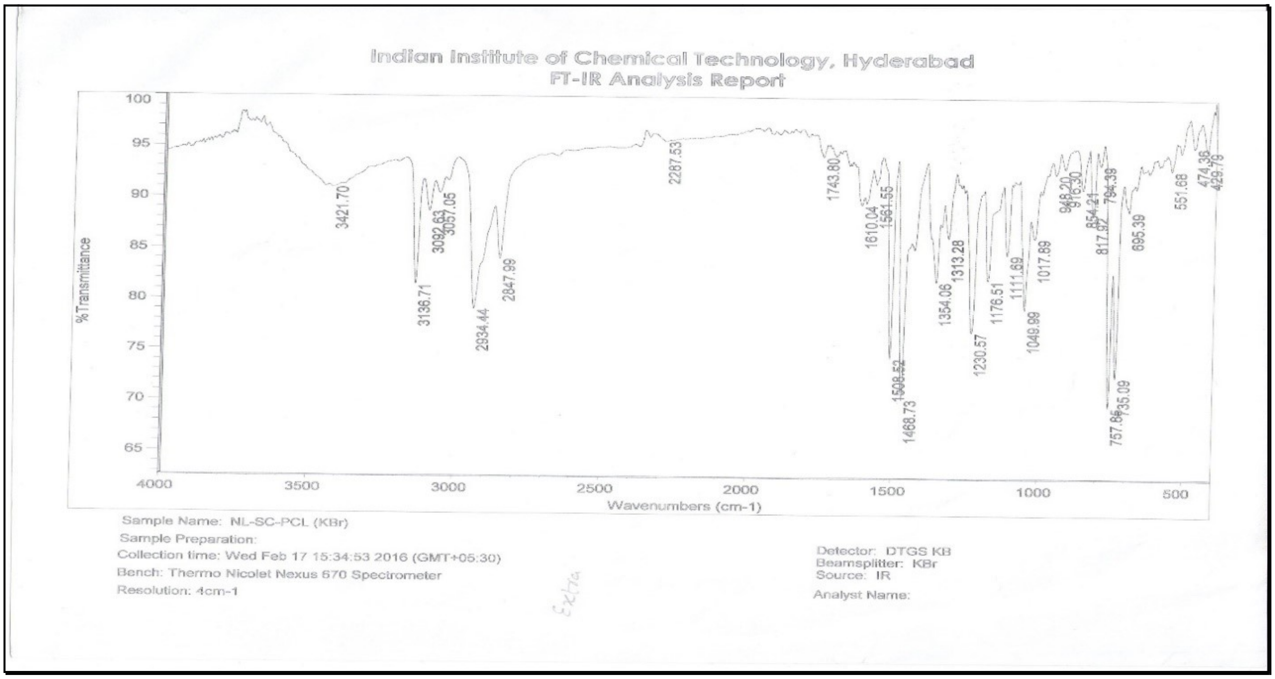

Figure S28: IR (KBr, cm^-) spectrum of compound 9-((1-(2-fluorophenyl)-1H-1,2,3-triazol-4-yl)methyl)-2,3,4,9-tetrahydro-1H-carbazole **(4f)**

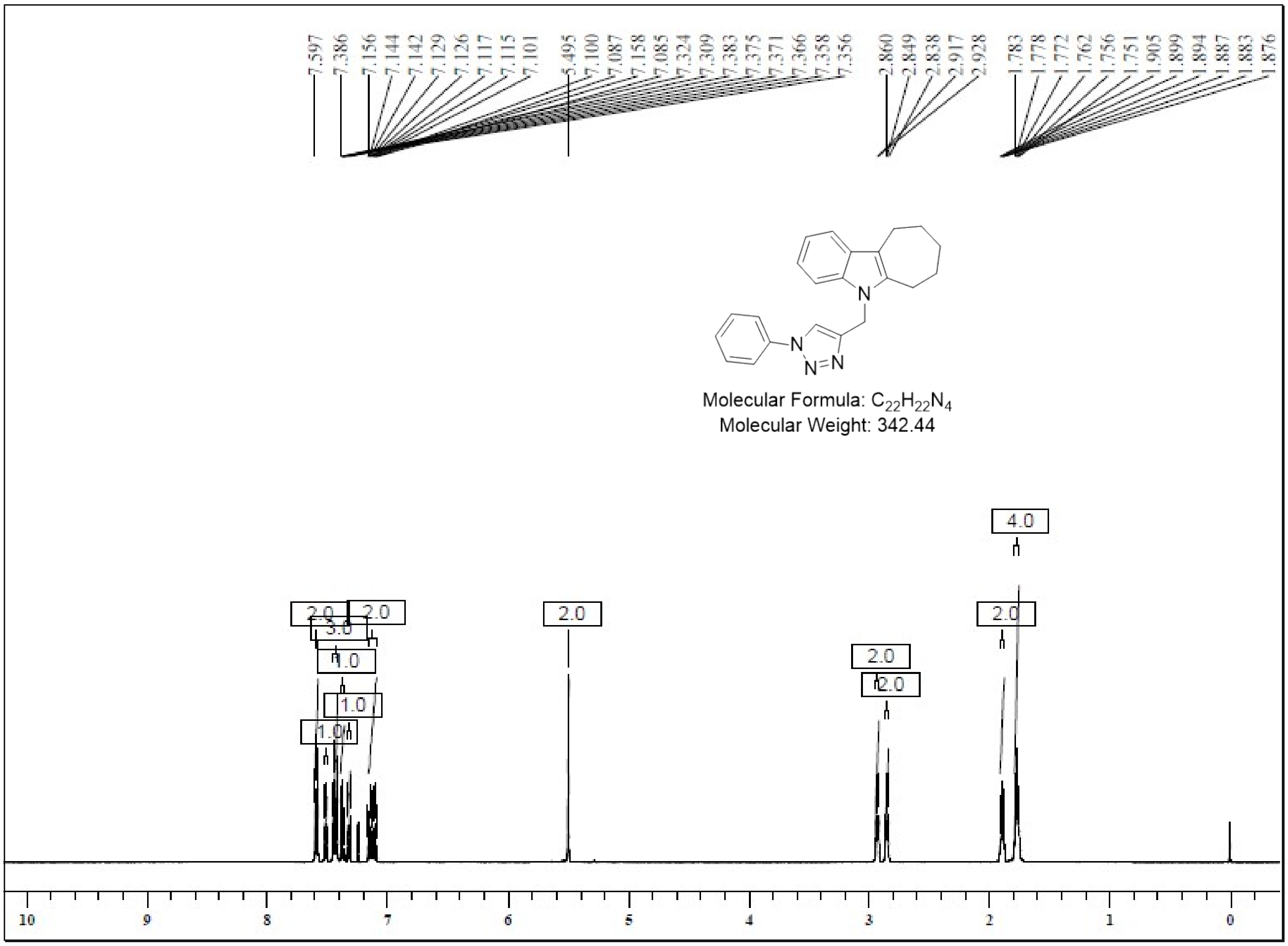

Figure S29: ^{1}H NMR (400 MHz, $CDCl_3$) of 5-((1-phenyl-1*H*-1,2,3-triazol-4-yl)methyl)-5,6,7,8,9,10-hexahydrocyclohepta[b]indole **(4g)**

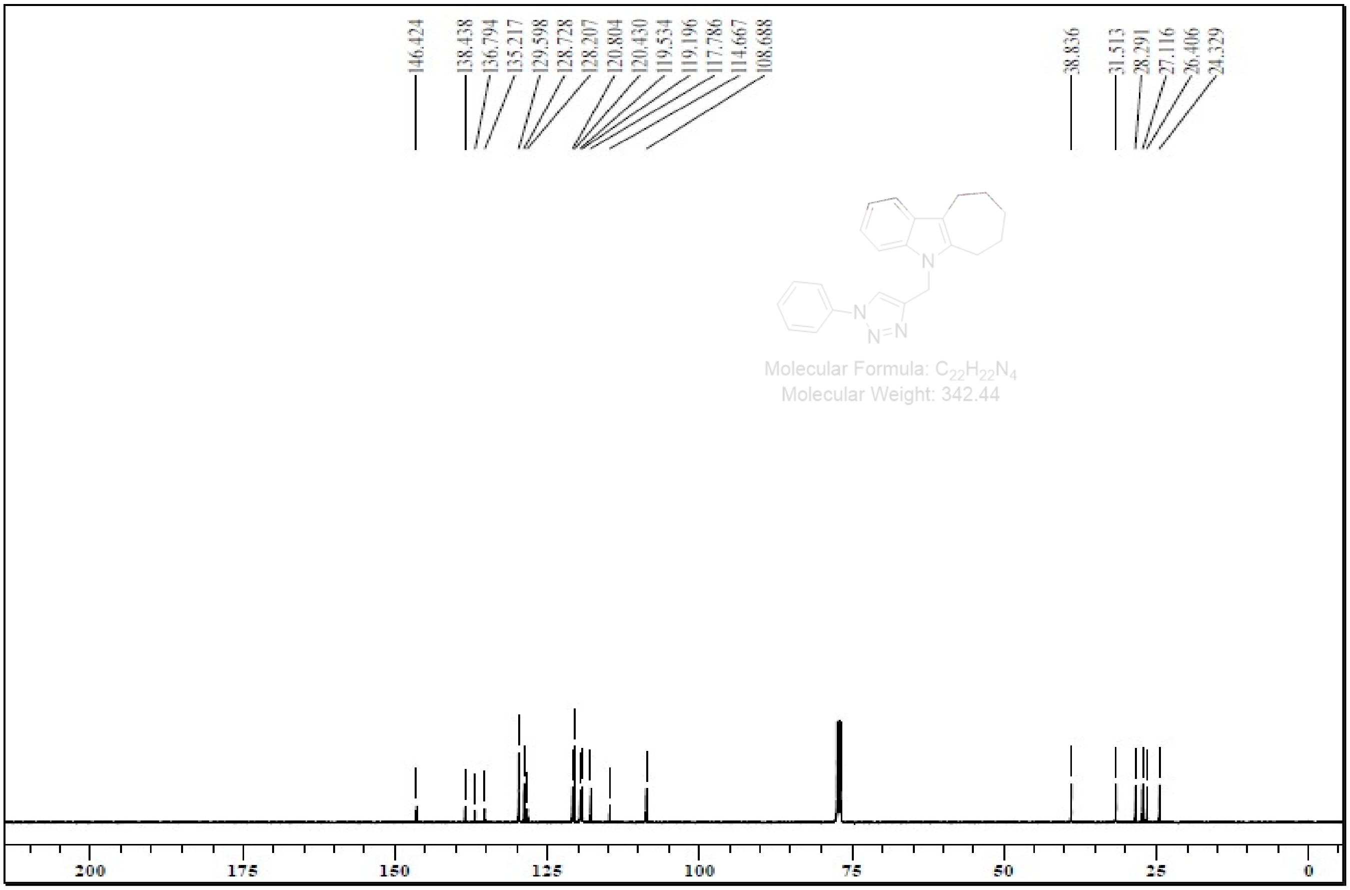

Figure S30: ^{13}C NMR (100 MHz, $CDCl_3$) spectrum of 5-((1-phenyl-1*H*-1,2,3-triazol-4-yl)methyl) 5,6,7,8,9,10-hexahydrocyclohepta[b]indole **(4g)**

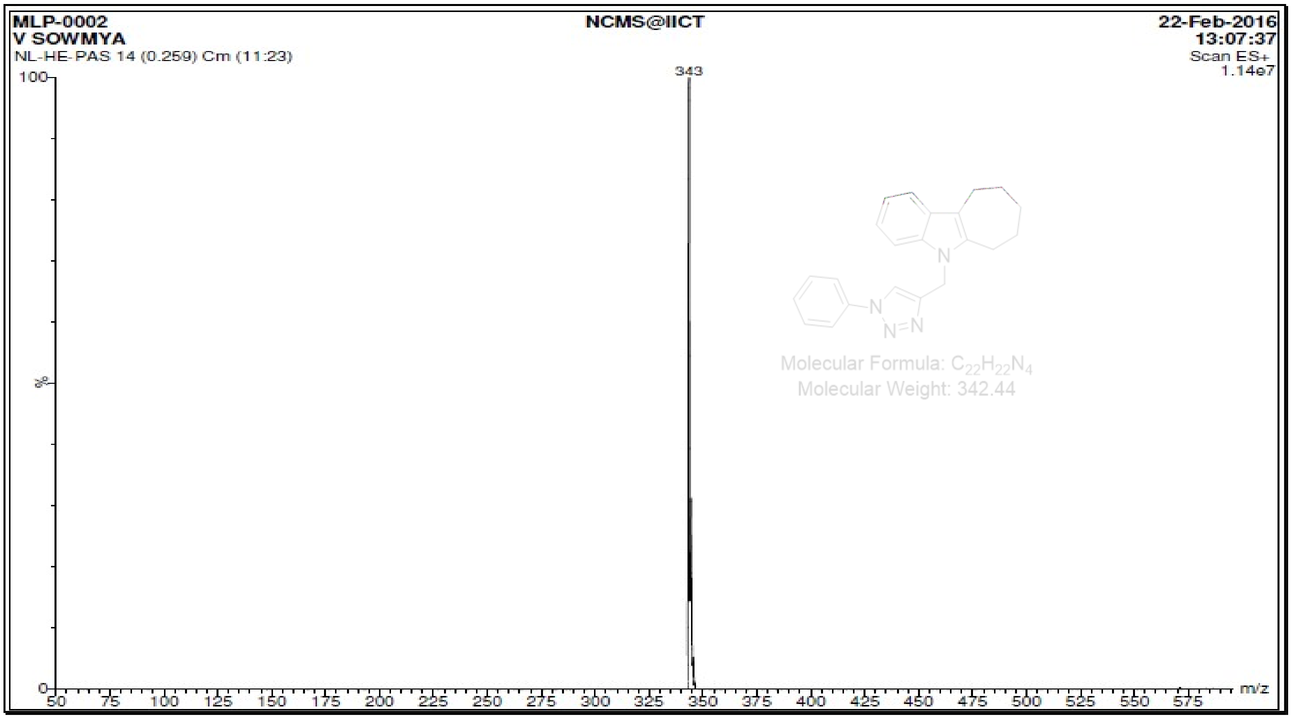

Figure S31: Mass (ES^+) spectrum of 5-((1-phenyl-1*H*-1,2,3-triazol-4-yl)methyl)-5,6,7,8,9,10-hexahydrocyclohepta[b]indole **(4g)**

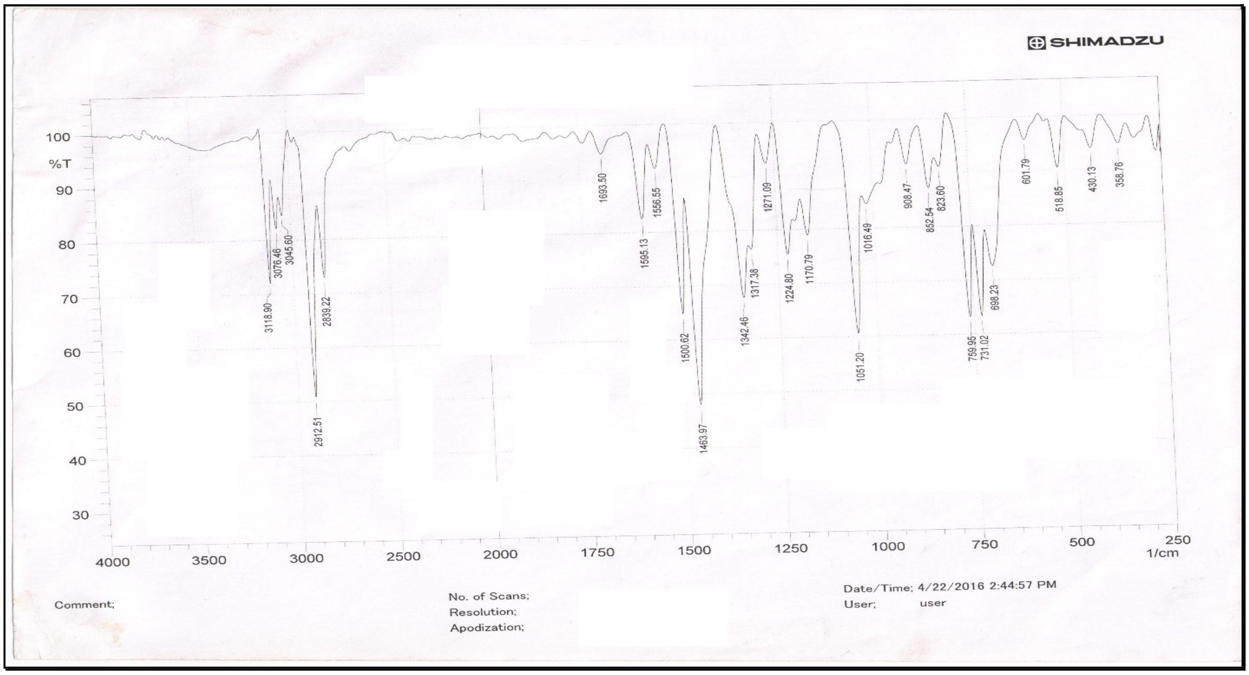

Figure S32: IR (KBr, cm^{-}) spectrum of compound 5-((1-phenyl-1*H*-1,2,3-triazol-4-yl)methyl)-5,6,7,8,9,10-hexahydrocyclohepta[b]indole **(4g)**

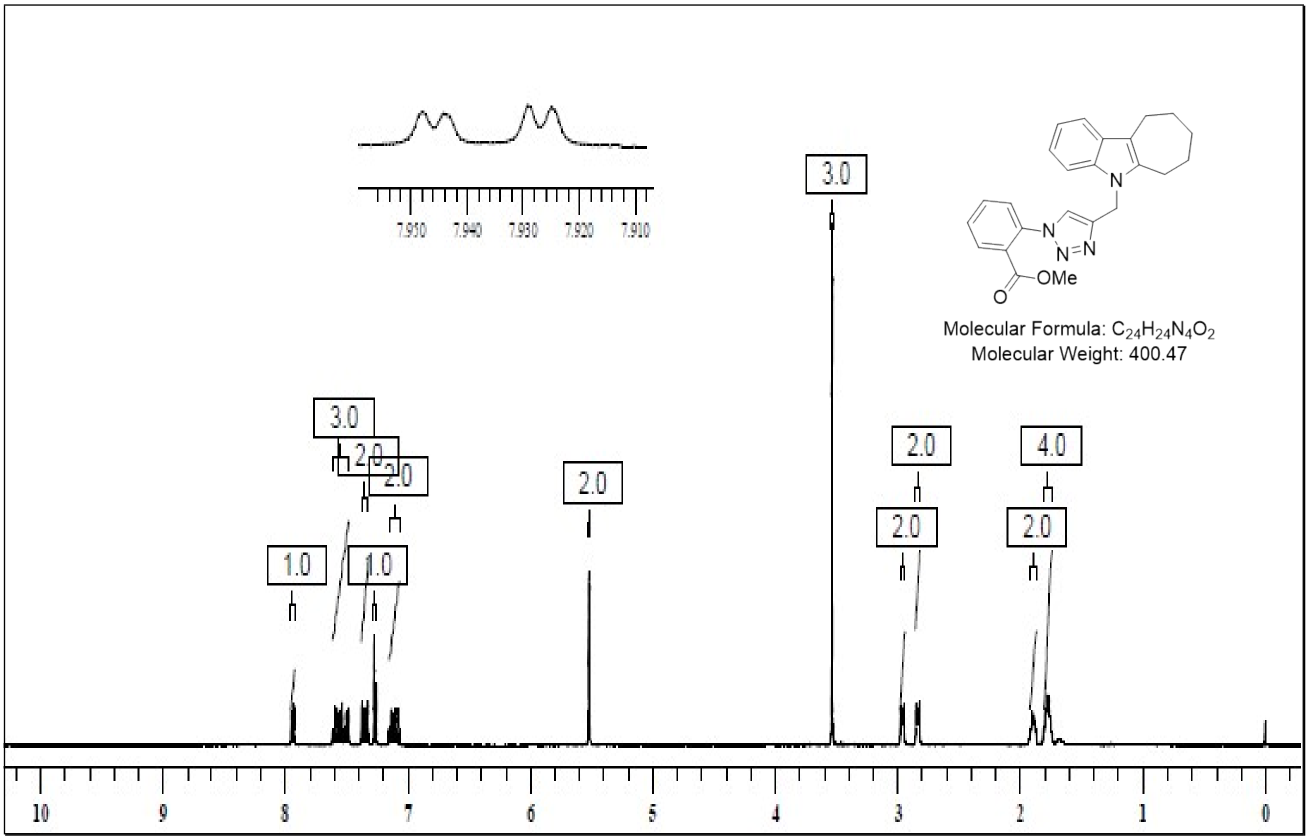

Figure S33: ¹H NMR (400 MHz, $CDCl_3$) of methyl 2-(4-((7,8,9,10-tetrahydrocyclohepta[b]indol-5(6*H*)-yl)methyl)-1*H*-1,2,3-triazol-1-yl)benzoate **(4h)**

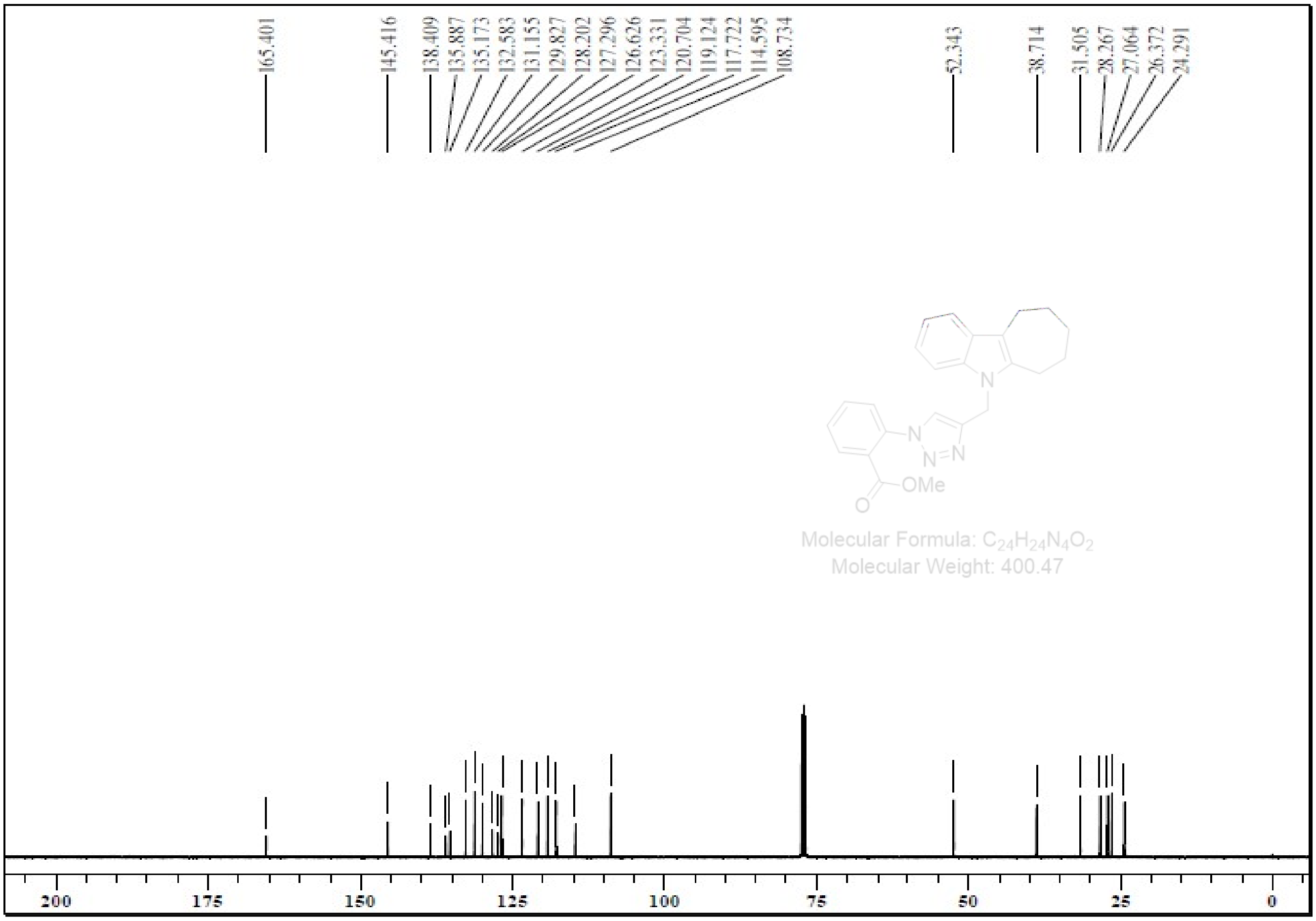

Figure S34: ^{13}C NMR (100 MHz, $CDCl_3$) spectrum of methyl 2-(4-((7,8,9,10-tetrahydrocyclohepta[b]indol-5(6*H*)-yl)methyl)-1*H*-1,2,3-triazol-1-yl)benzoate **(4h)**

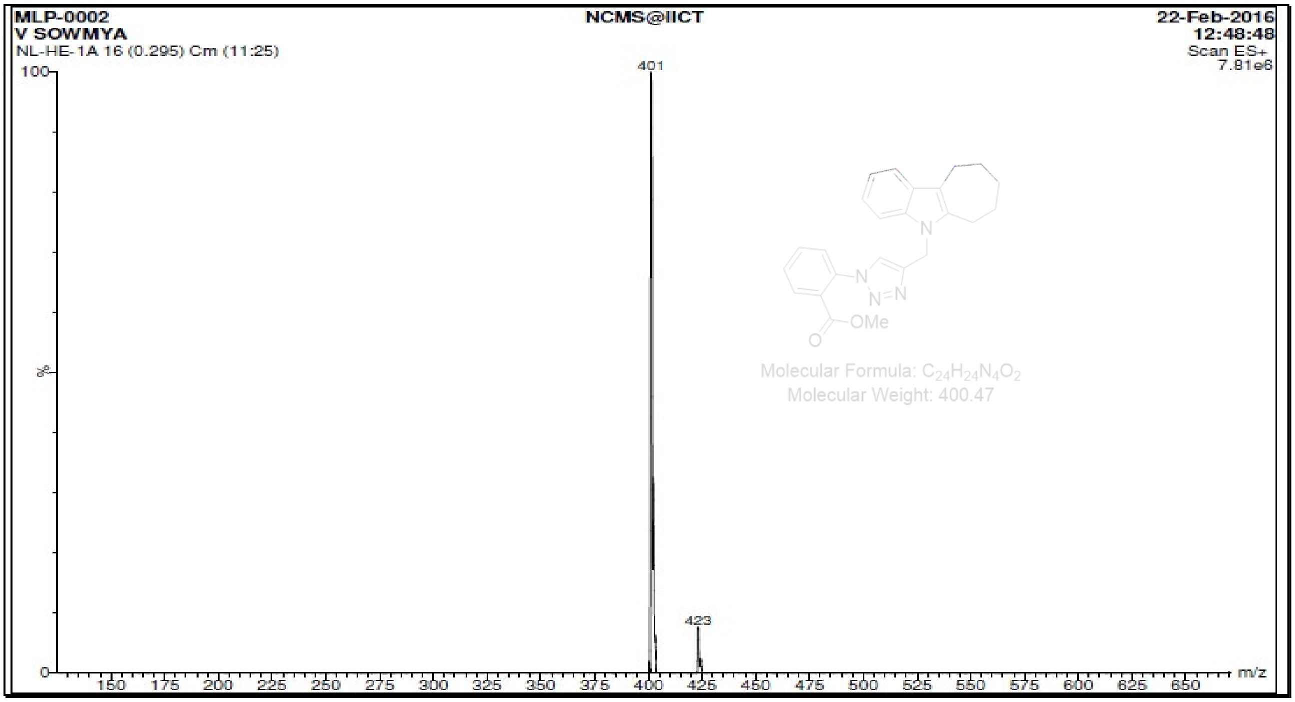

Figure S35: Mass (ES$^+$) spectrum of compound methyl 2-(4-((7,8,9,10-tetrahydrocyclohepta[b]indol-5(6*H*)-yl)methyl)-1*H*-1,2,3-triazol-1-yl)benzoate **(4h)**

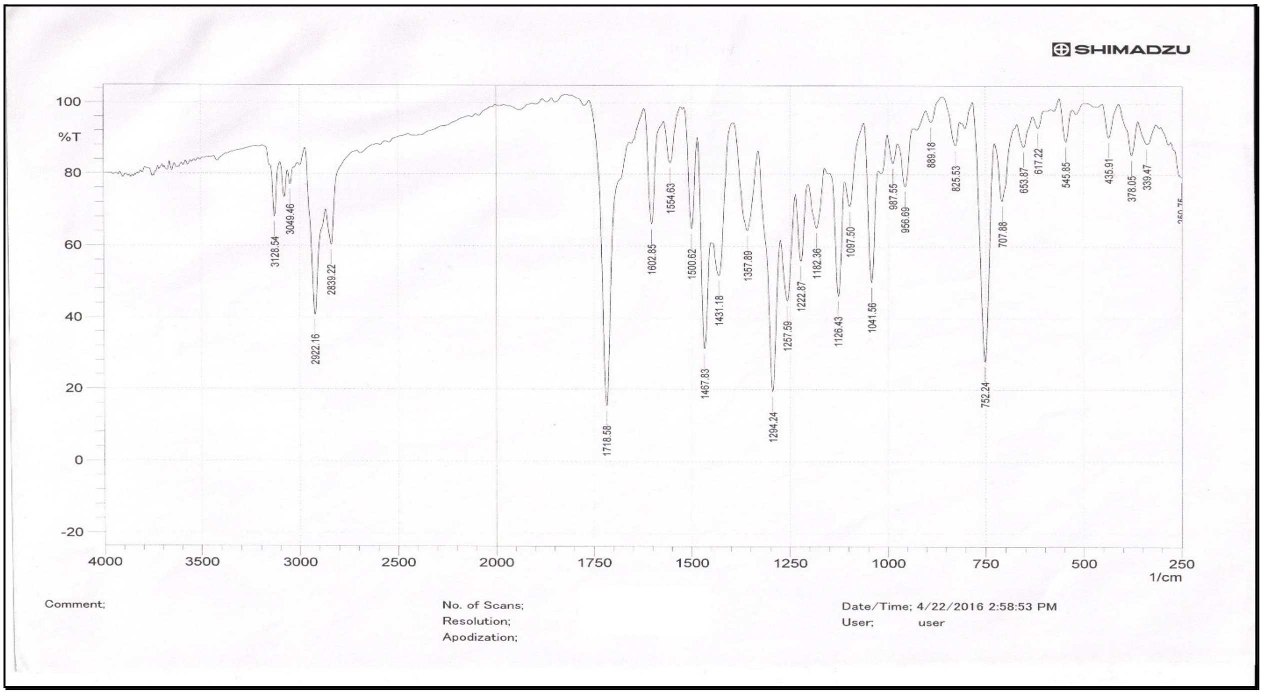

Figure S36: IR (KBr, cm$^-$) spectrum of compound methyl 2-(4-((7,8,9,10-tetrahydrocyclohepta[b]indol-5(6*H*)-yl)methyl)-1*H*-1,2,3-triazol-1-yl)benzoate **(4h)**

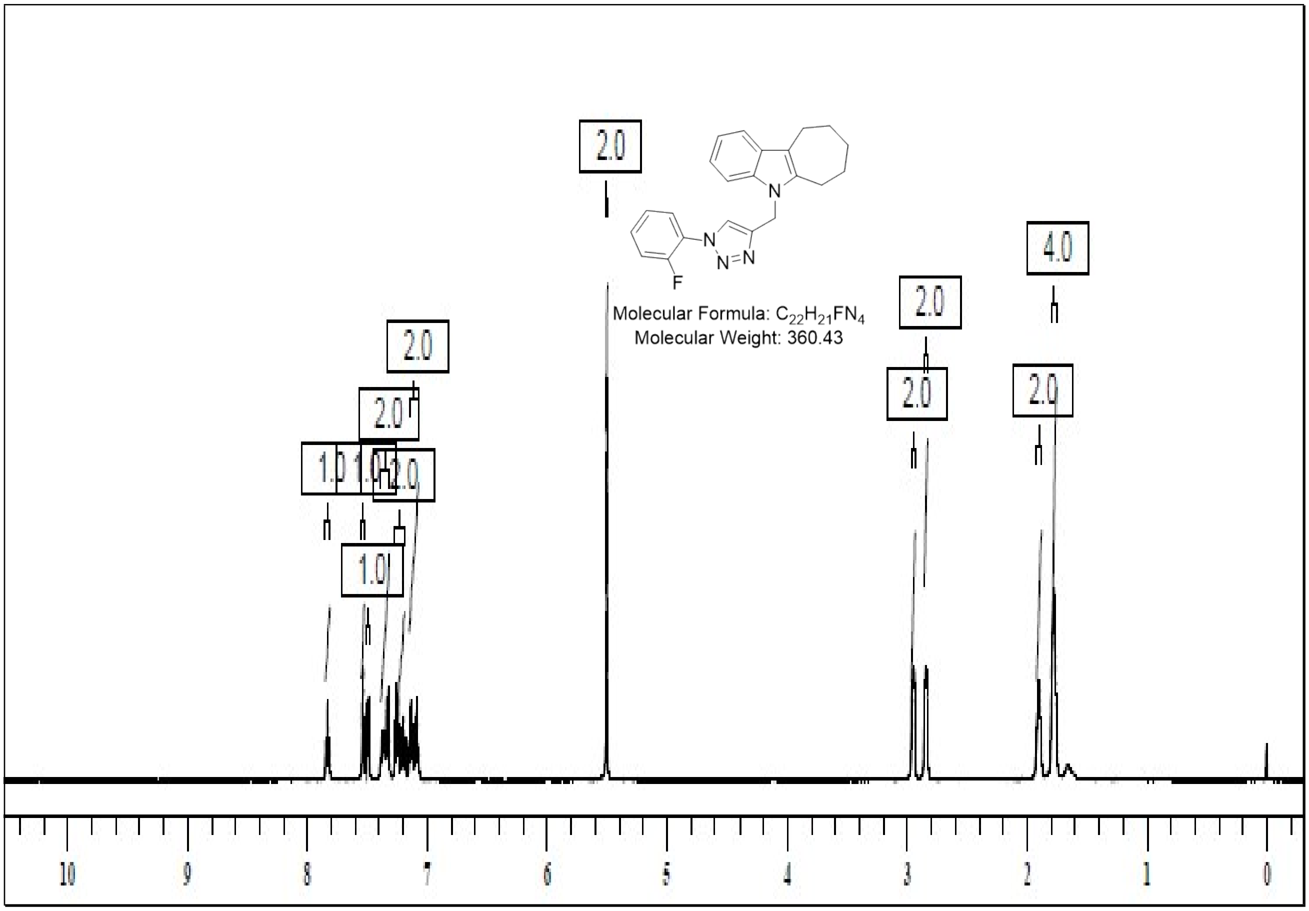

Figure S37: ^{1}H NMR (400 MHz, $CDCl_3$) of 5-((1-(2-fluorophenyl)-1*H*-1,2,3-triazol-4-yl)methyl)-5,6,7,8,9,10-hexahydrocyclohepta[b]indole **(4i)**

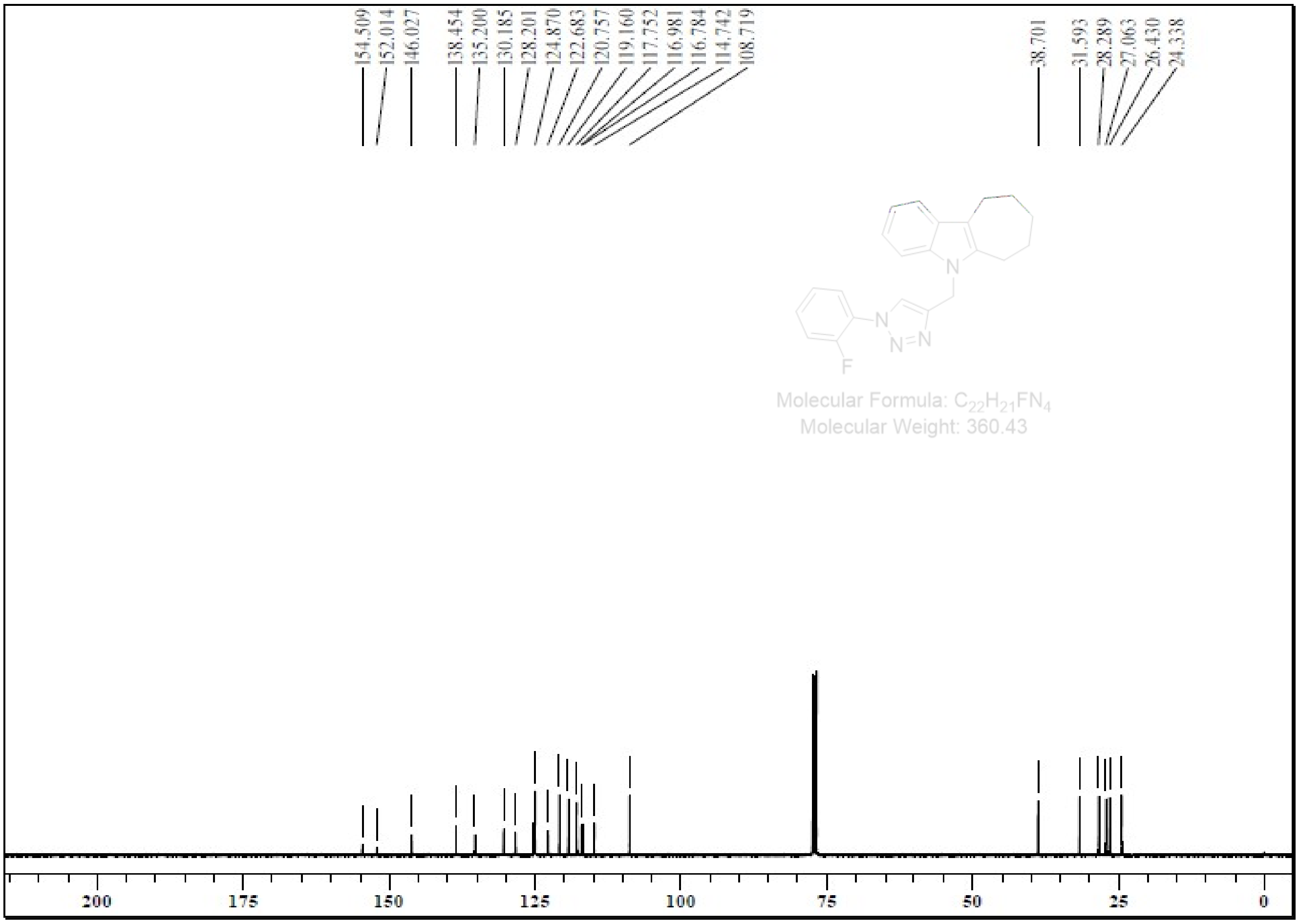

Figure S38: ^{13}C NMR (100 MHz, $CDCl_3$) spectrum of 5-((1-(2-fluorophenyl)-1*H*-1,2,3-triazol-4-yl)methyl)-5,6,7,8,9,10-hexahydrocyclohepta[b]indole **(4i)**

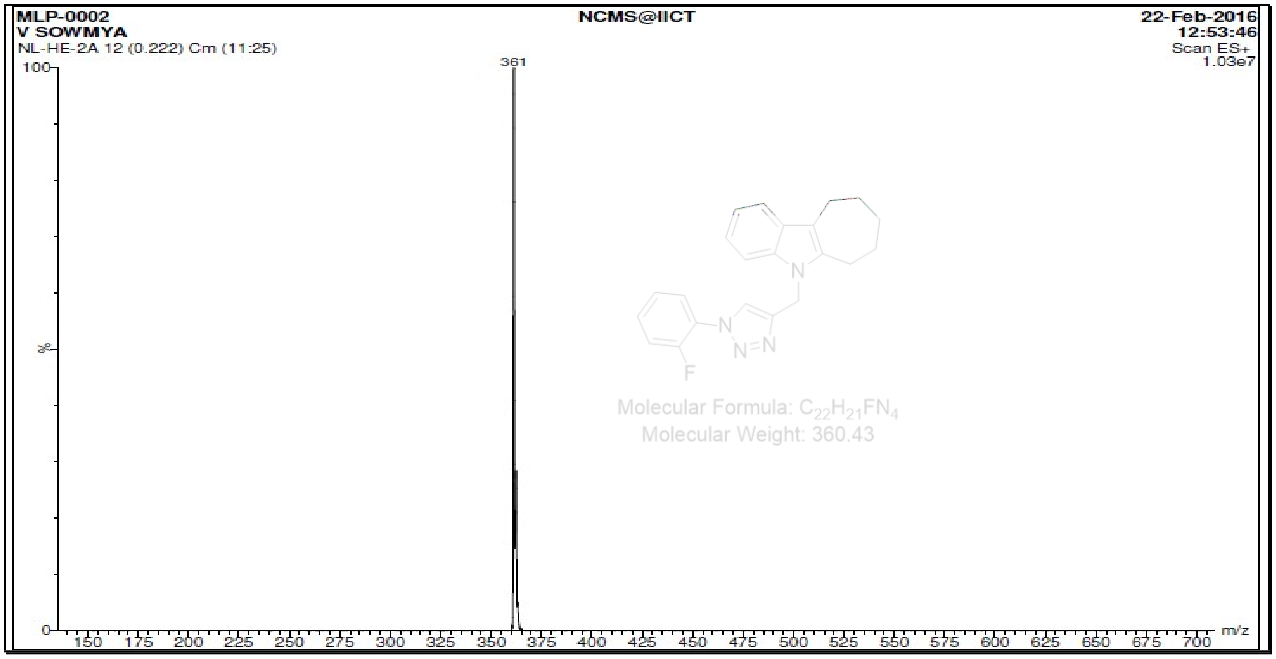

Figure S39: Mass (ES^+) spectrum of 5-((1-(2-fluorophenyl)-1*H*-1,2,3-triazol-4-yl)methyl)-5,6,7,8,9,10-hexahydrocyclohepta[b]indole **(4i)**

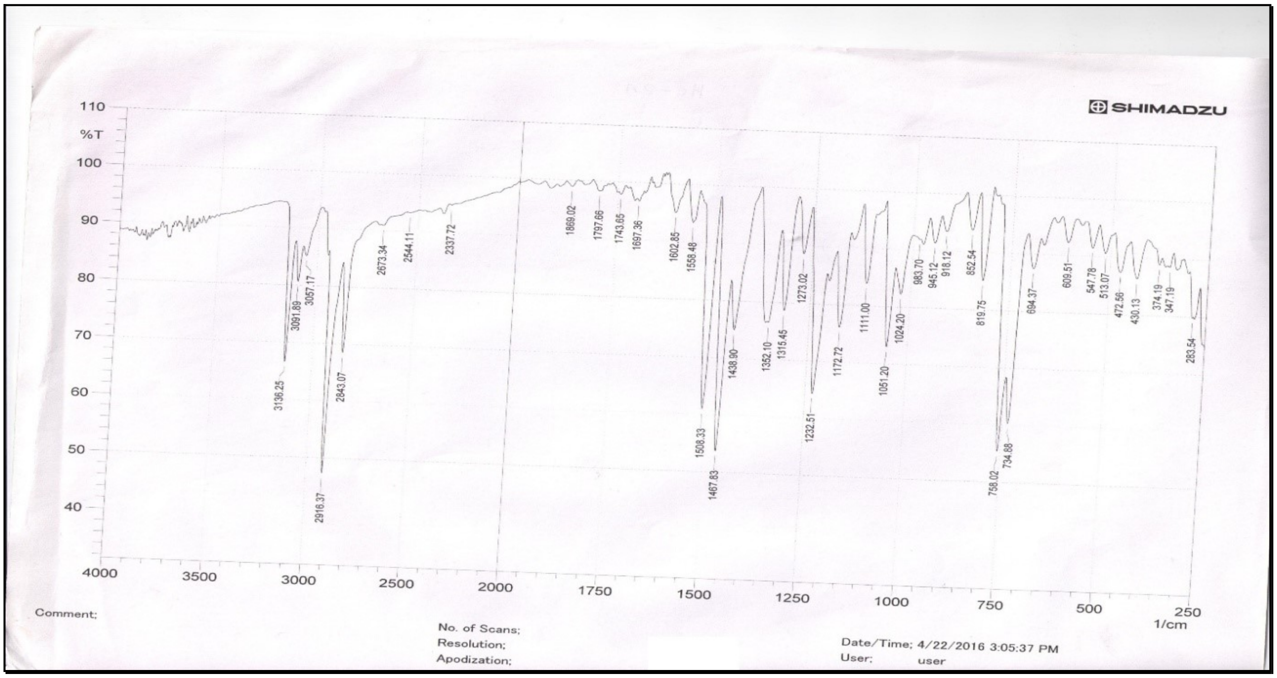

Figure S40: IR (KBr, cm^{-}) spectrum of 5-((1-(2-fluorophenyl)-1*H*-1,2,3-triazol-4-yl)methyl)-5,6,7,8,9,10-hexahydrocyclohepta[b]indole **(4i)**

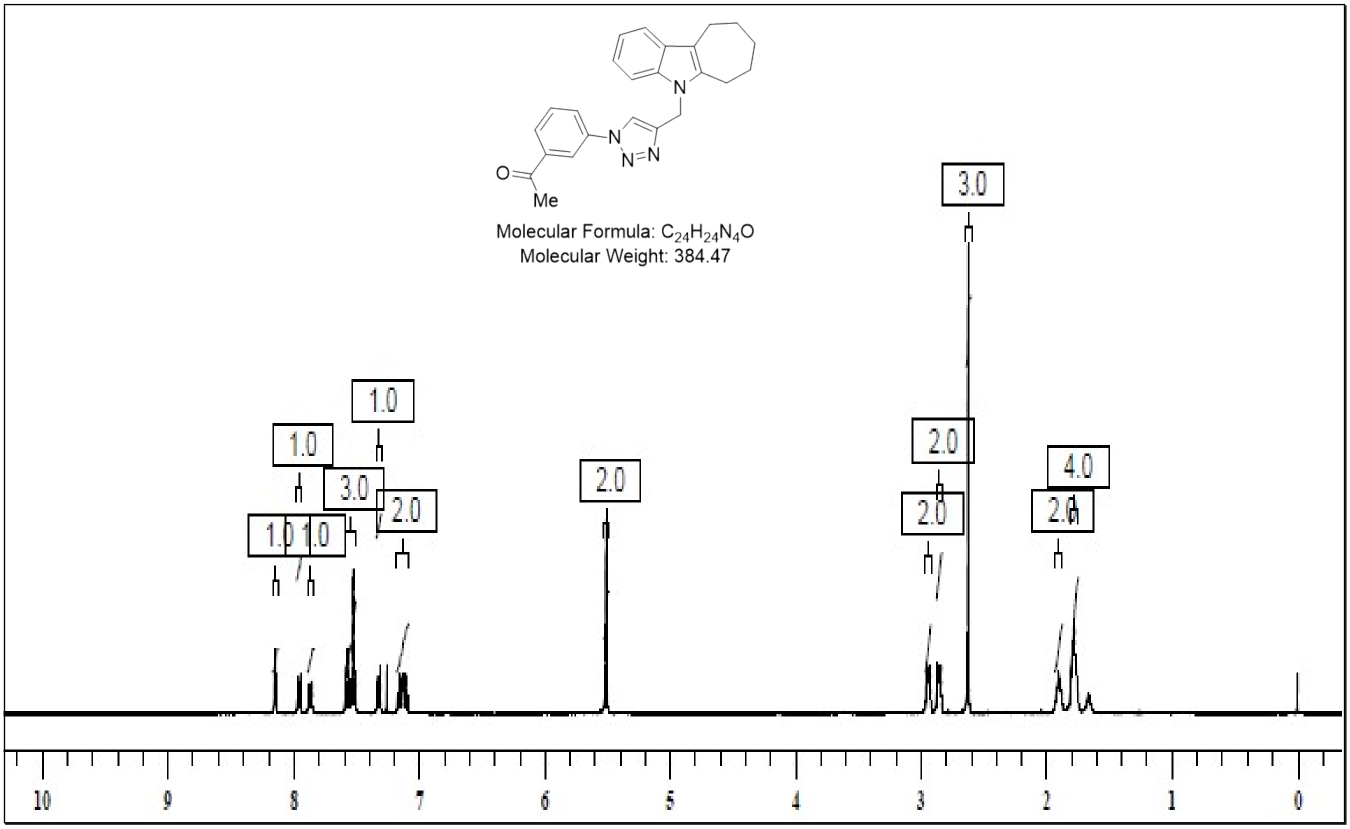

Figure S41: ^{1}H NMR (400 MHz, $CDCl_3$) of 1-(3-(4-((7,8,9,10-tetrahydrocyclohepta[b]indol-5(6*H*)-yl)methyl)-1*H*-1,2,3-triazol-1-yl)phenyl)ethanone **(4j)**

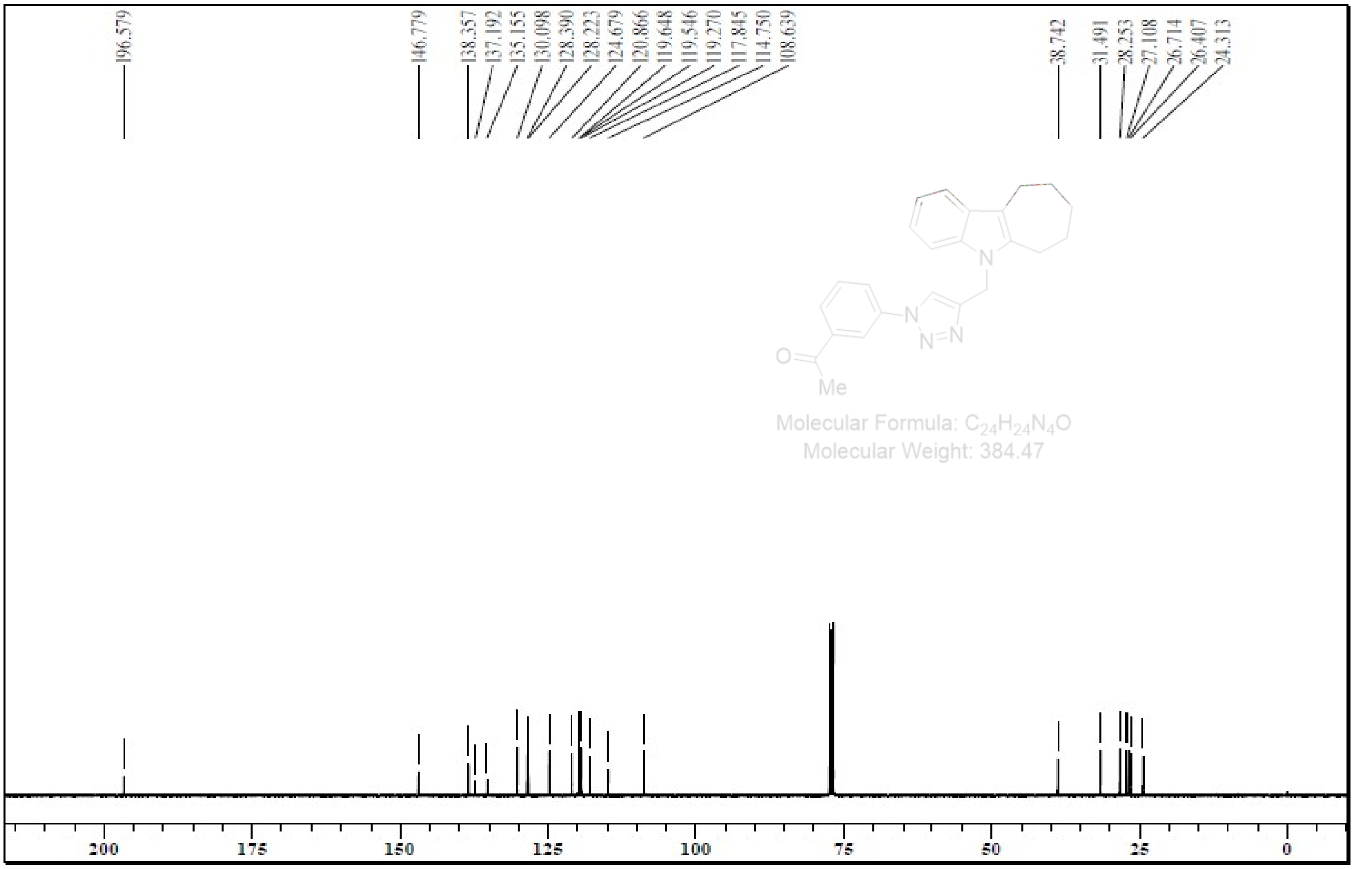

Figure S42: ^{13}C NMR (100 MHz, $CDCl_3$) spectrum of 1-(3-(4-((7,8,9,10-tetrahydrocyclohepta[b]indol-5(6*H*)-yl)methyl)-1*H*-1,2,3-triazol-1-yl)phenyl)ethanone **(4j)**

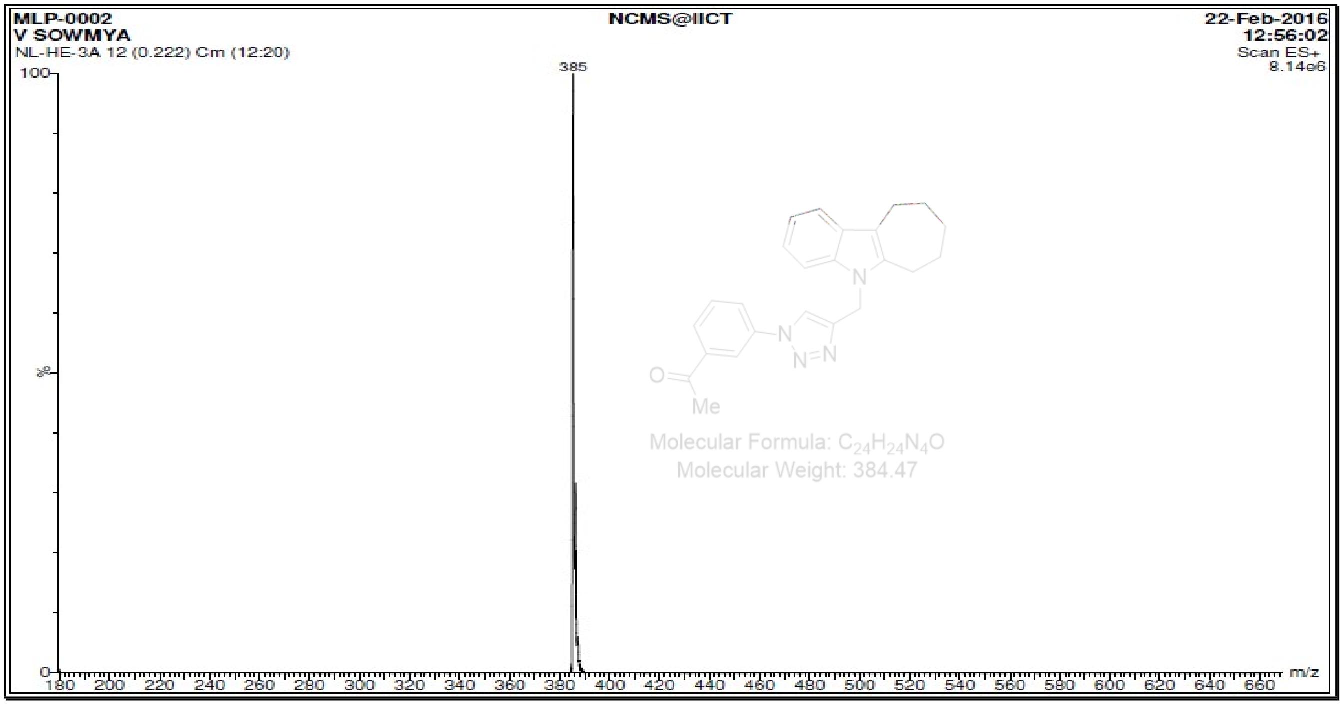

Figure S43: Mass (ES^+) spectrum of 1-(3-(4-((7,8,9,10-tetrahydrocyclohepta[b]indol-5(6*H*)-yl)methyl)-1*H*-1,2,3-triazol-1-yl)phenyl)ethanone **(4j)**

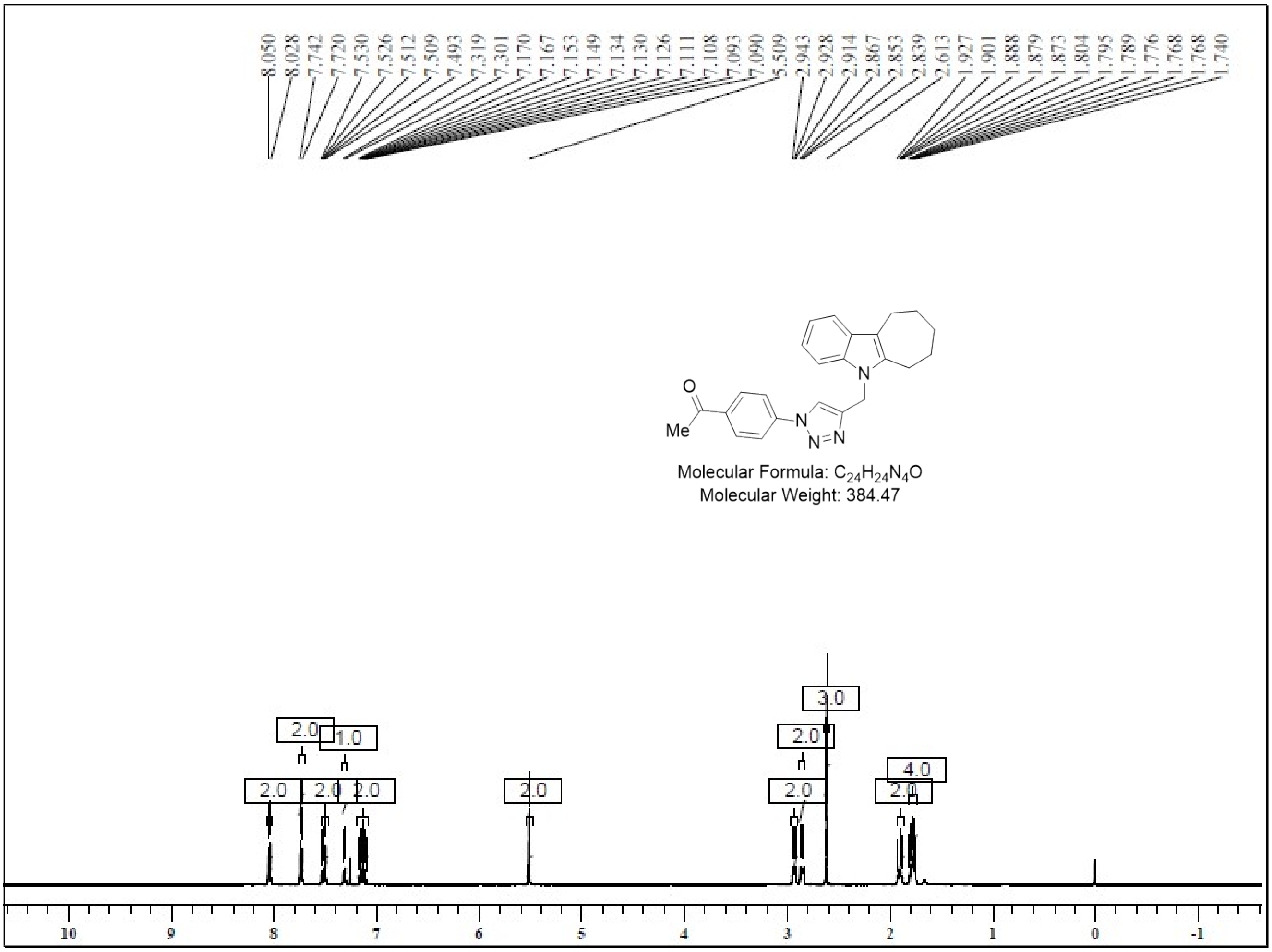

Figure S44: ^{1}H NMR (400 MHz, $CDCl_3$) of 1-(4-(4-((7,8,9,10-tetrahydrocyclohepta[b]indol-5(6*H*)-yl)methyl)-1*H*-1,2,3-triazol-1-yl)phenyl)ethanone **(4k)**

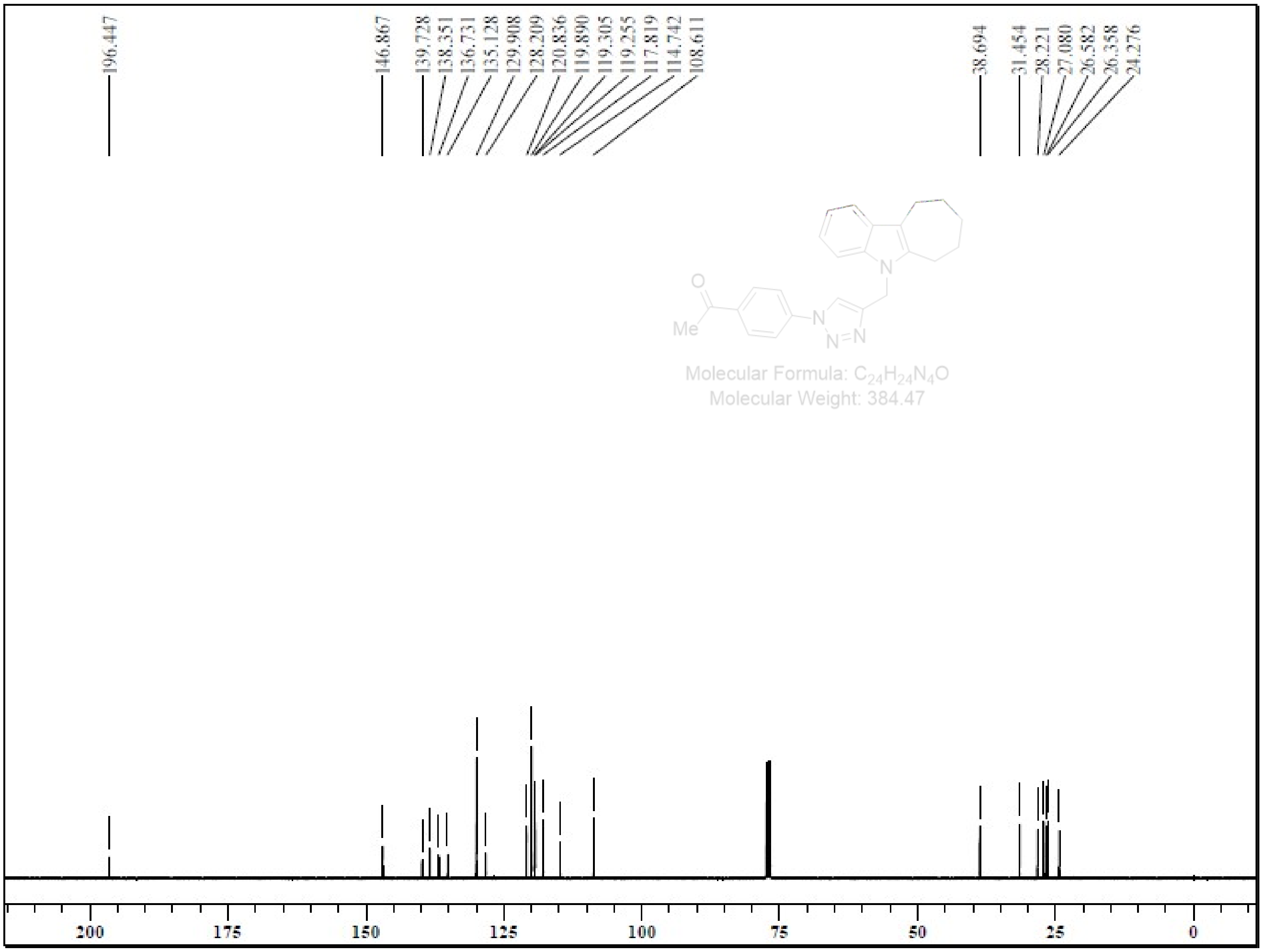

Figure S45: ^{13}C NMR (100 MHz, $CDCl_3$) spectrum of 1-(4-(4-((7,8,9,10-tetrahydrocyclohepta[b]indol-5(6*H*)-yl)methyl)-1*H*-1,2,3-triazol-1-yl)phenyl)ethanone**(4k)**

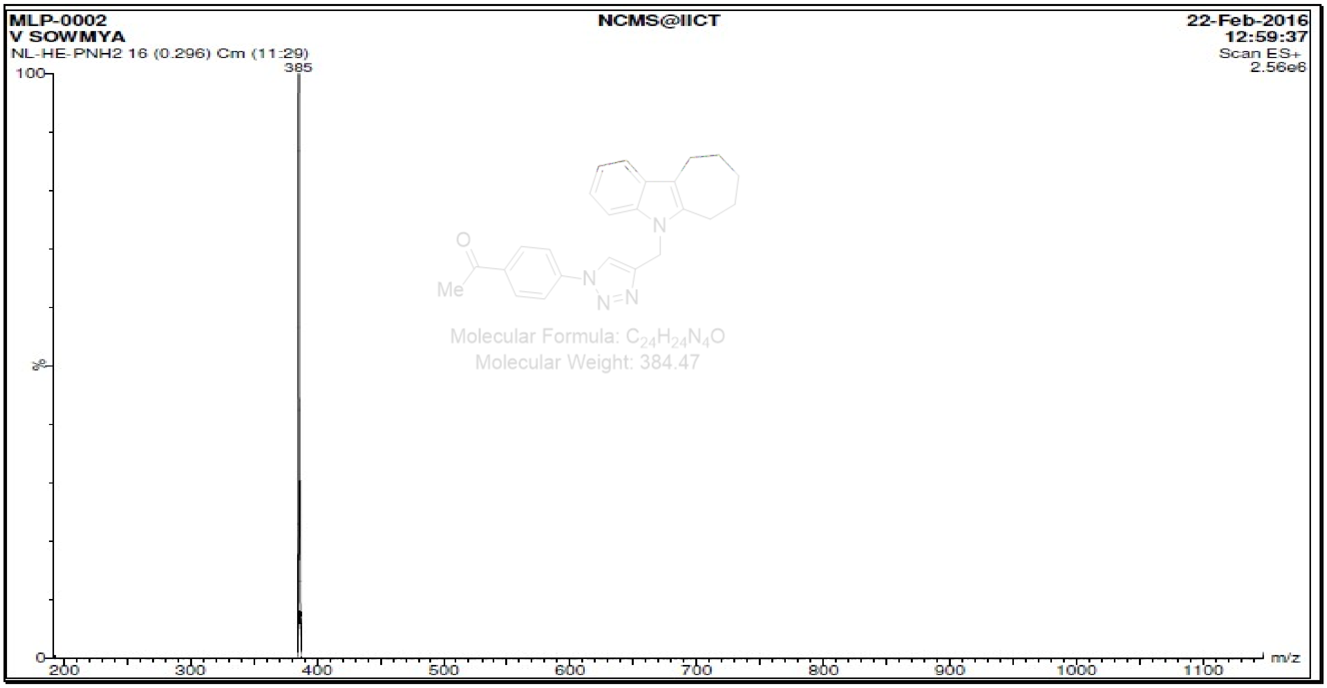

Figure S46: Mass (ES$^+$) spectrum of 1-(4-(4-((7,8,9,10-tetrahydrocyclohepta[b]indol-5(6*H*)-yl)methyl)-1*H*-1,2,3-triazol-1-yl)phenyl)ethanone9 **(4k)**

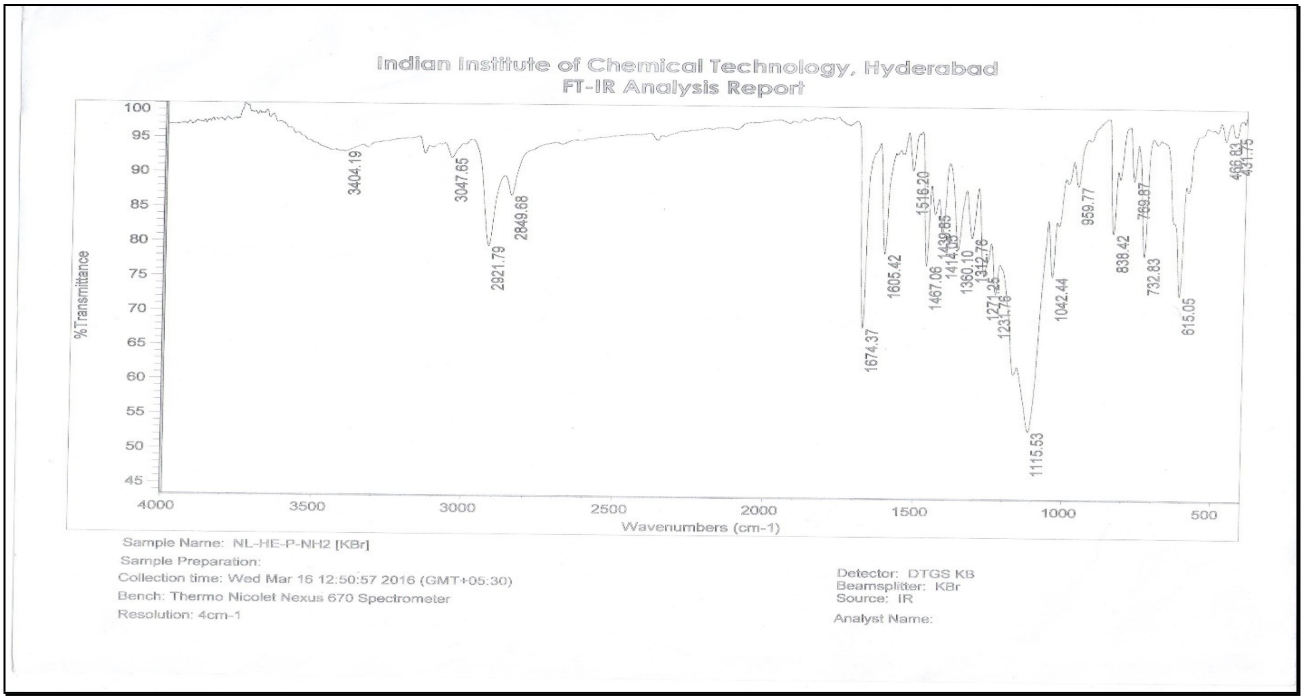

Figure S47: IR (KBr, cm$^-$) spectrum of 1-(4-(4-((7,8,9,10-tetrahydrocyclohepta[b]indol-5(6*H*)-yl)methyl)-1*H*-1,2,3-triazol-1-yl)phenyl)ethanone **(4k)**

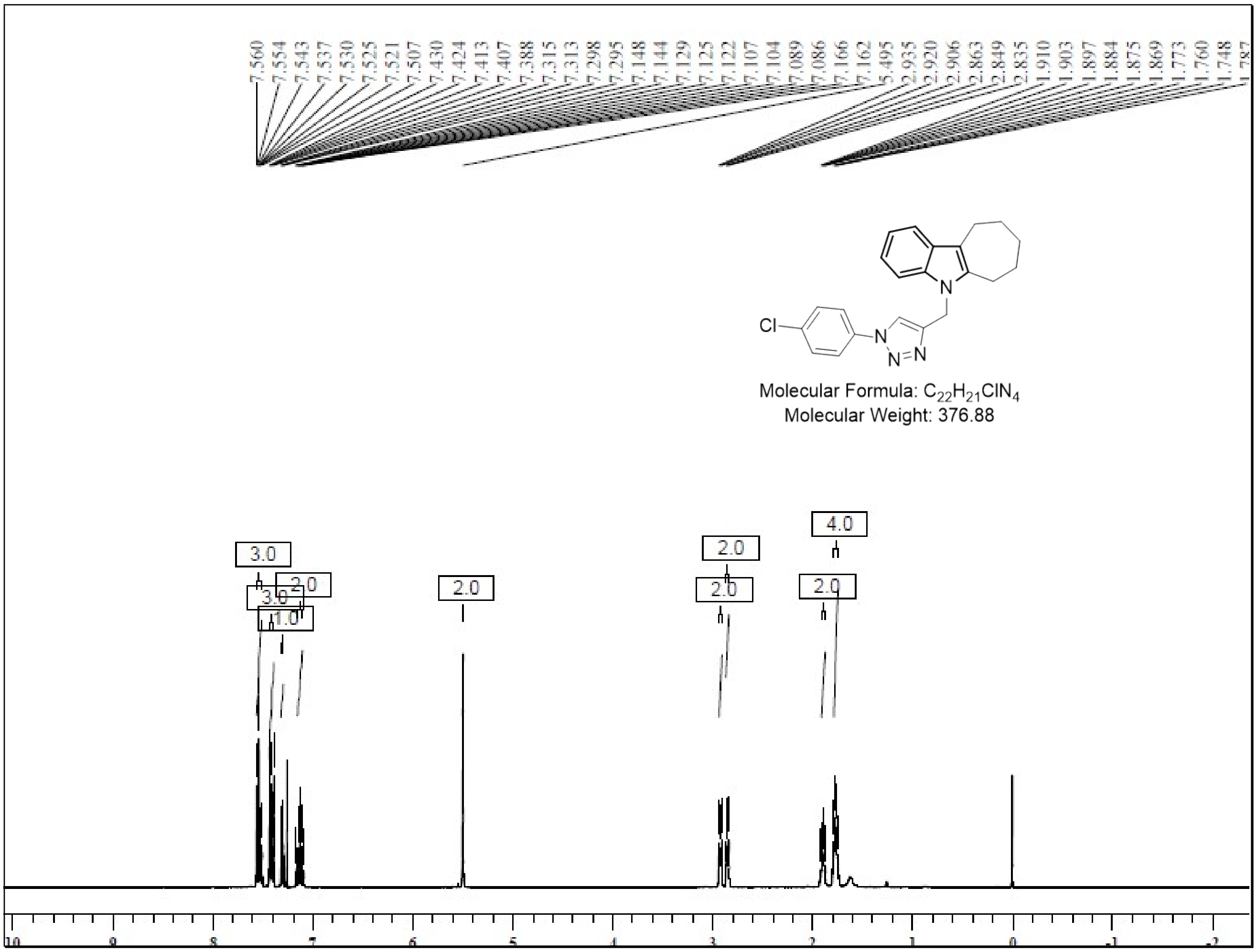

Figure S48: ^{1}H NMR (400 MHz, $CDCl_3$) of 5-((1-(4-chlorophenyl)-1*H*-1,2,3-triazol-4-yl)methyl)-5,6,7,8,9,10-hexahydrocyclohepta[b]indole **(4l)**

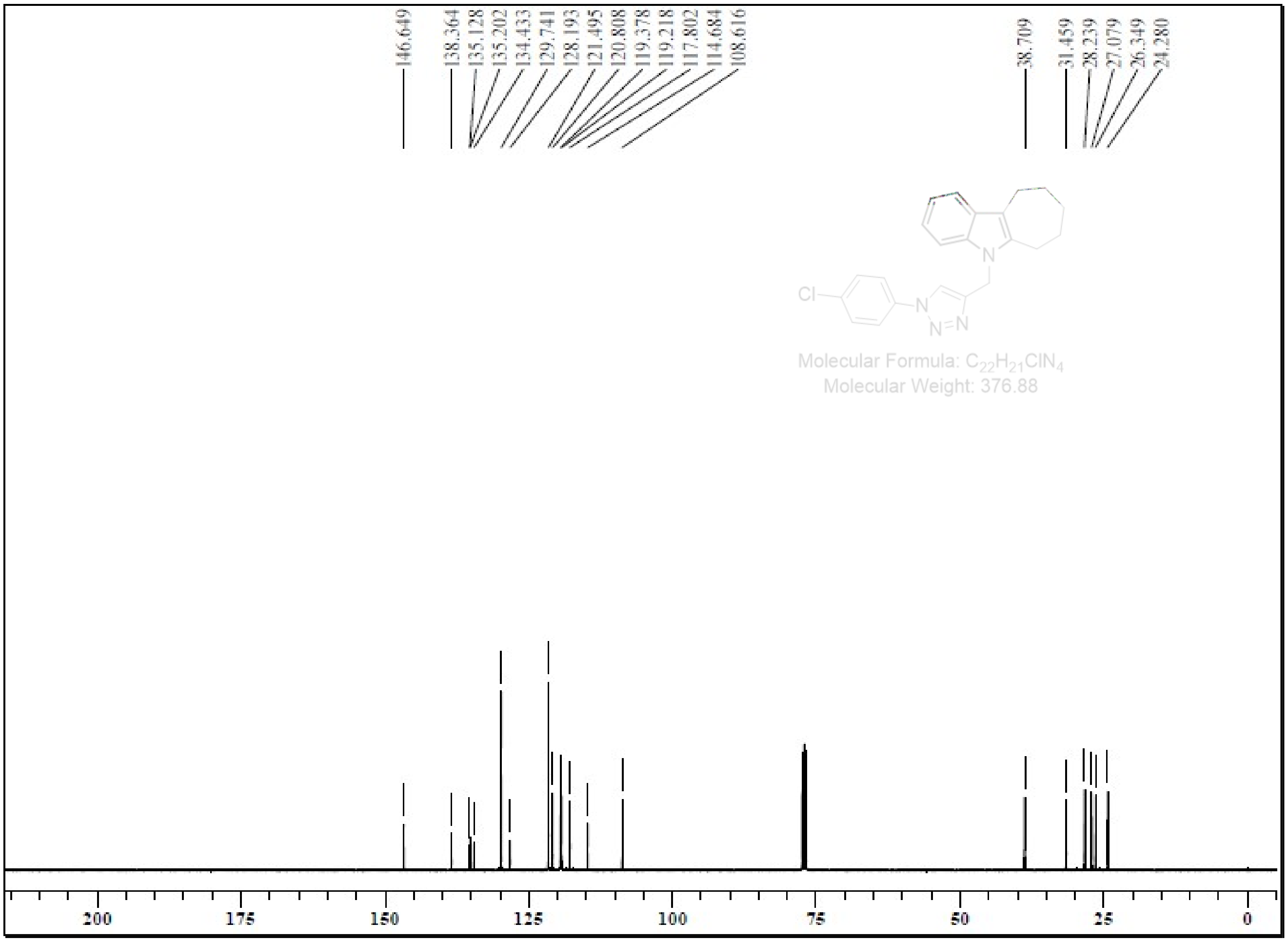

Figure S49: ^{13}C NMR (100 MHz, $CDCl_3$) spectrum of 5-((1-(4-chlorophenyl)-1*H*-1,2,3-triazol-4-yl)methyl)-5,6,7,8,9,10-hexahydrocyclohepta[b]indole **(4l)**

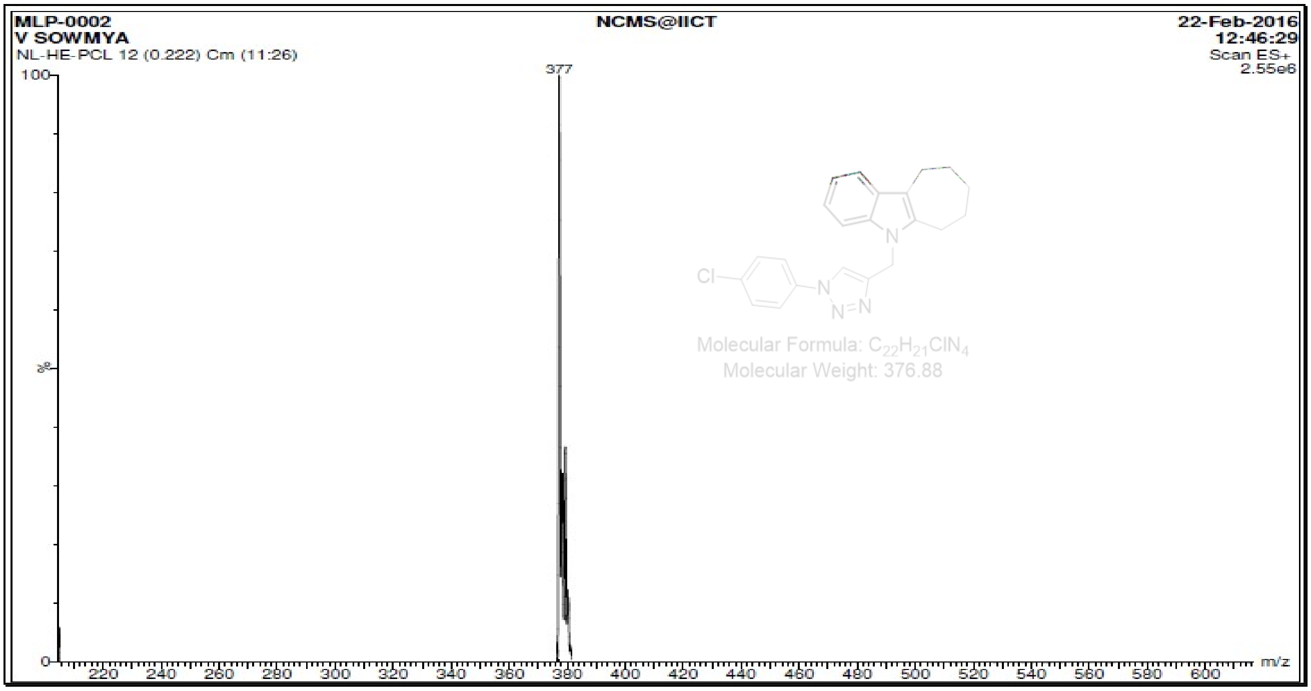

Figure S50: Mass (ES^{+}) spectrum of 5-((1-(4-chlorophenyl)-1*H*-1,2,3-triazol-4-yl)methyl)-5,6,7,8,9,10-hexahydrocyclohepta[b]indole **(4l)**

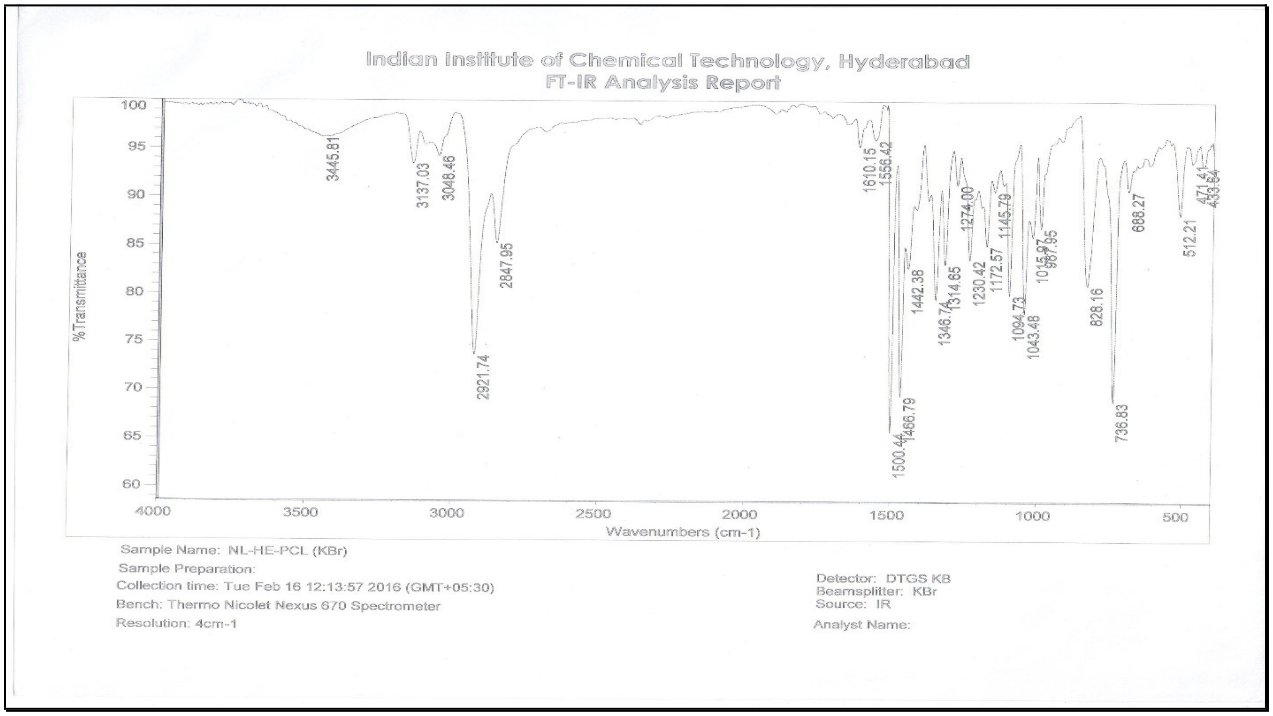

Figure S51: IR (KBr, cm^{-}) spectrum of 5-((1-(4-chlorophenyl)-1*H*-1,2,3-triazol-4-yl)methyl)-5,6,7,8,9,10-hexahydrocyclohepta[b]indole **(4l)**

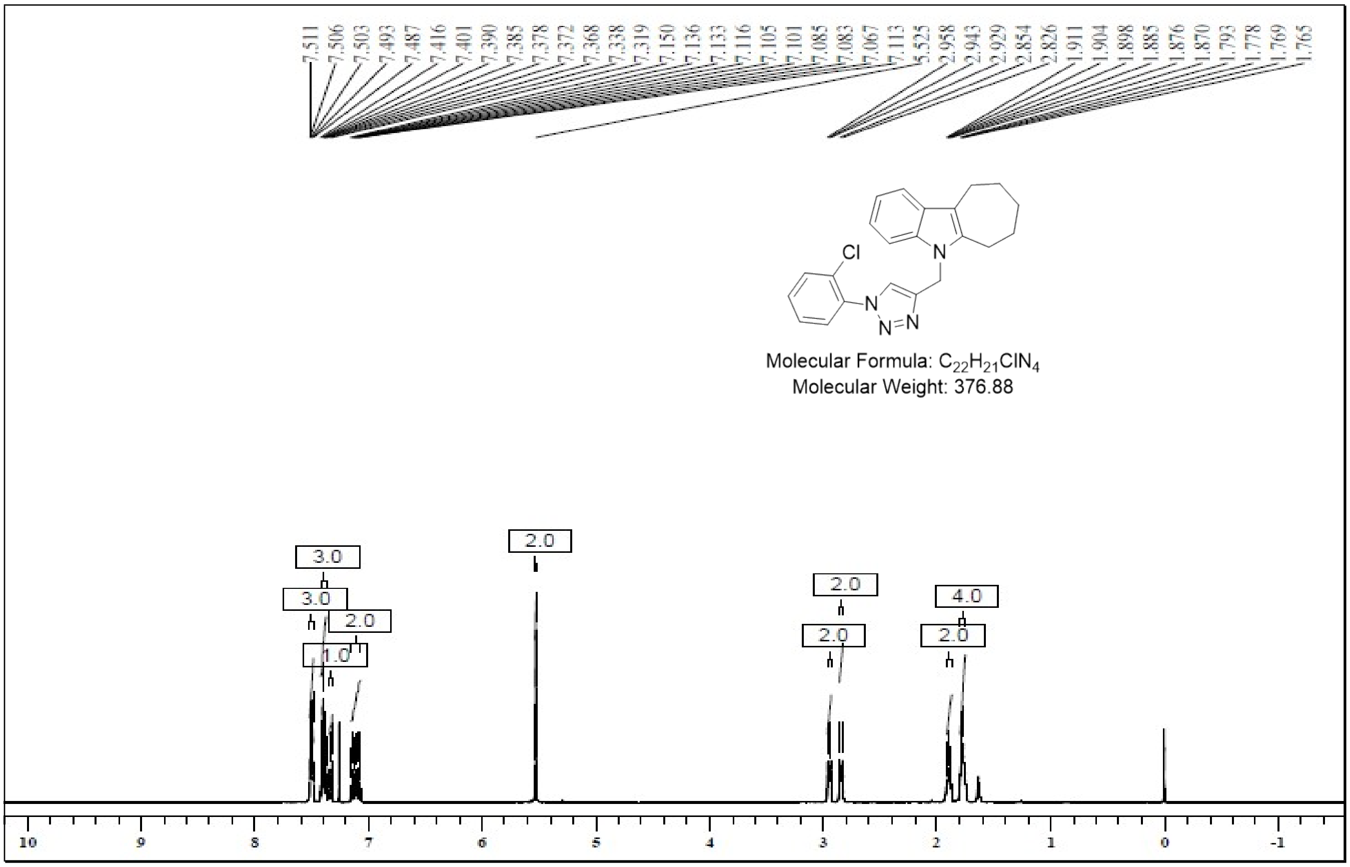

Figure S52: ^{1}H NMR (400 MHz, $CDCl_3$) of 4-(4-((7,8,9,10-tetrahydrocyclohepta[b]indol-5(6*H*)-yl)methyl)-1*H*-1,2,3-triazol-1-yl)phenyl hypochlorite **(4m)**

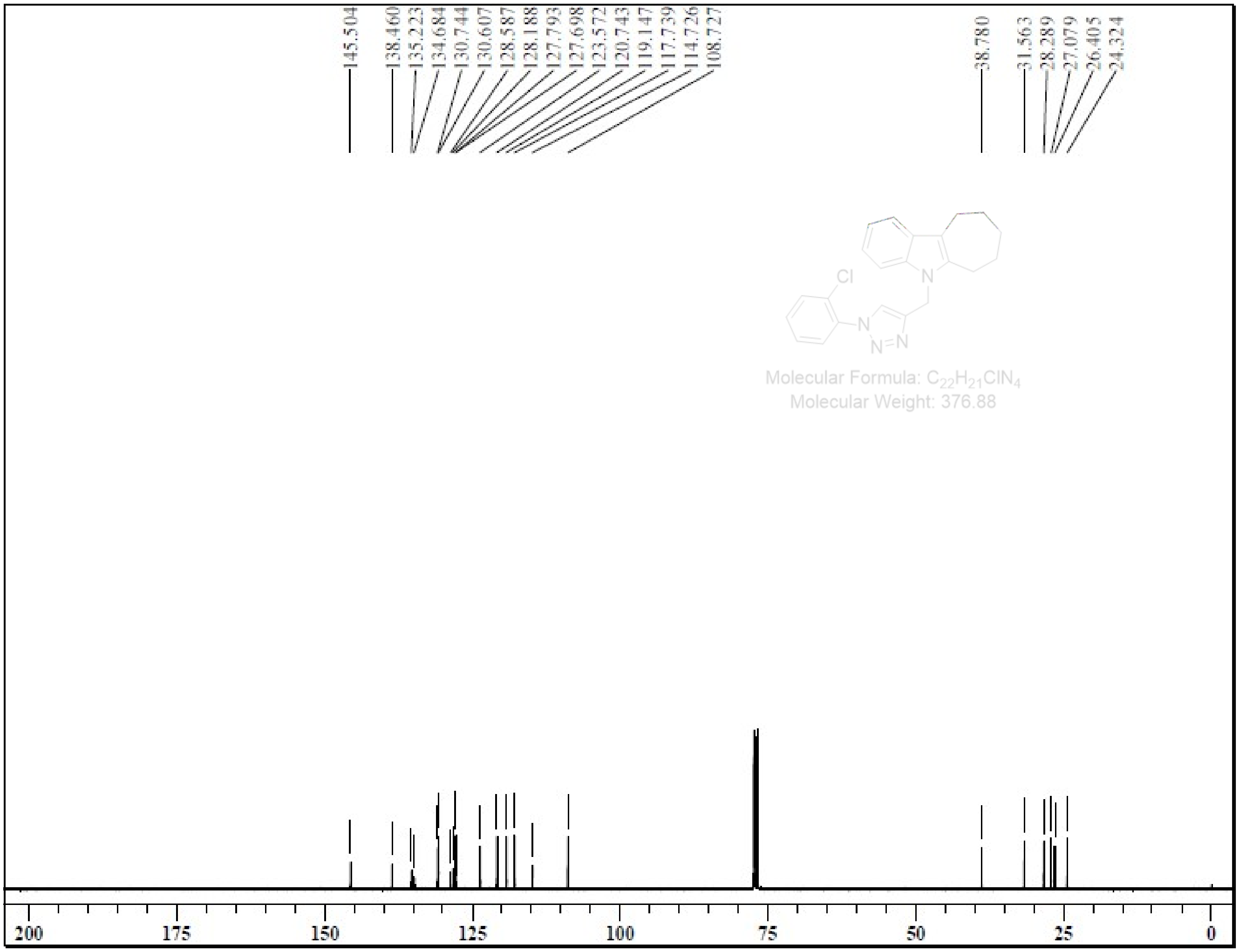

Figure S53: ^{13}C NMR (100 MHz, $CDCl_3$) of 4-(4-((7,8,9,10-tetrahydrocyclohepta[b]indol-5(6*H*)-yl)methyl)-1*H*-1,2,3-triazol-1-yl)phenyl hypochlorite **(4m)**

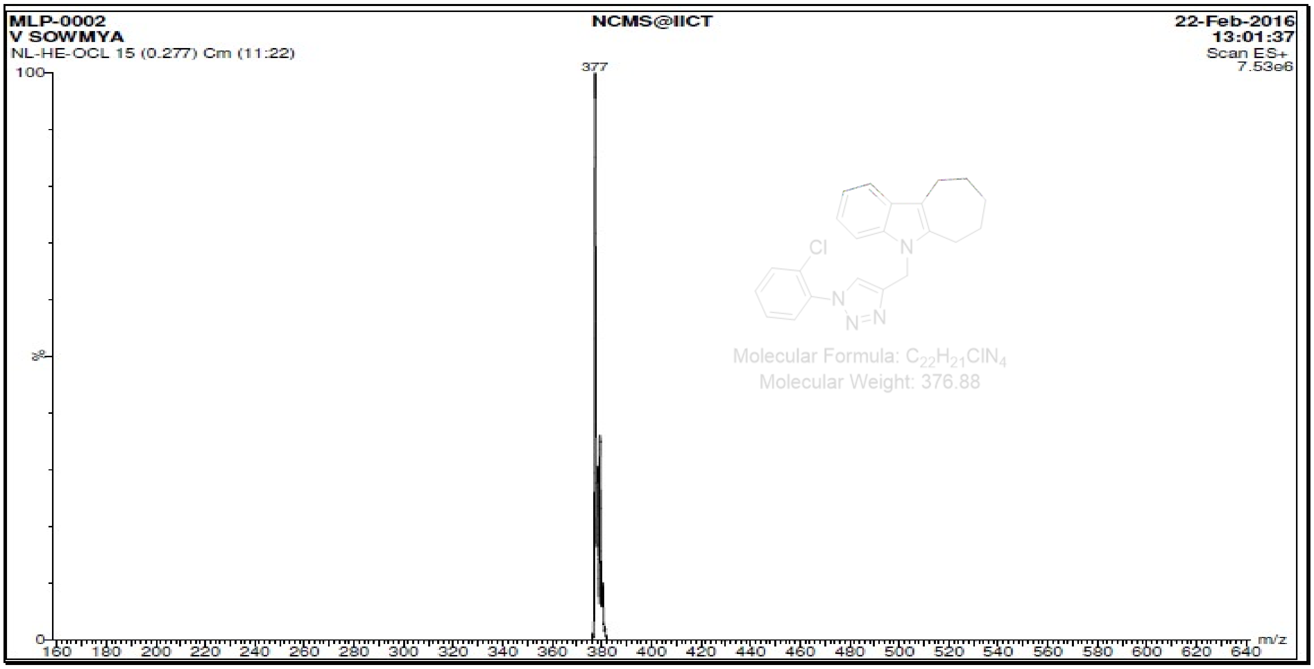

Figure S54: Mass (ES^+) spectrum of 4-(4-((7, 8, 9, 10-tetrahydrocyclohepta[b]indol-5(6*H*)-yl)methyl)- 1*H*-1,2,3-triazol-1-yl)phenyl hypochlorite **(4m)**

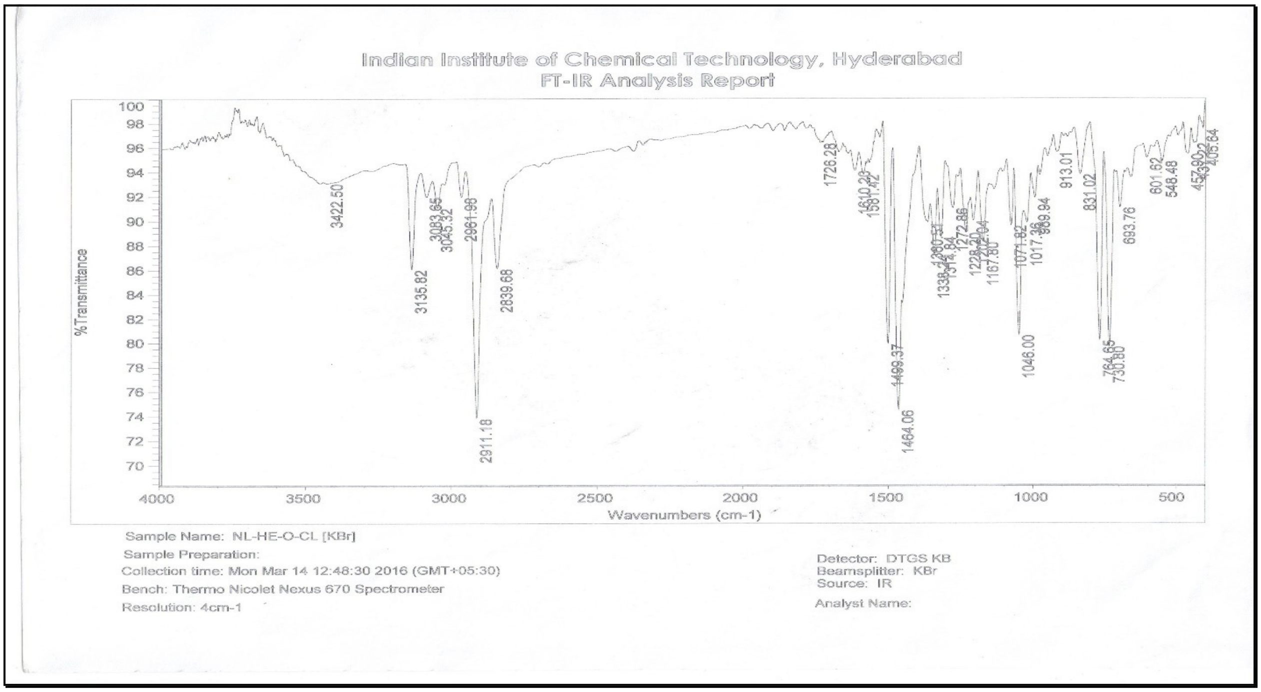

Figure S55: IR (KBr, cm^-) spectrum of 4-(4-((7,8,9,10-tetrahydrocyclohepta[b]indol-5(6*H*)-yl)methyl)-1*H*-1,2,3-triazol-1-yl)phenyl hypochlorite **(4m)**

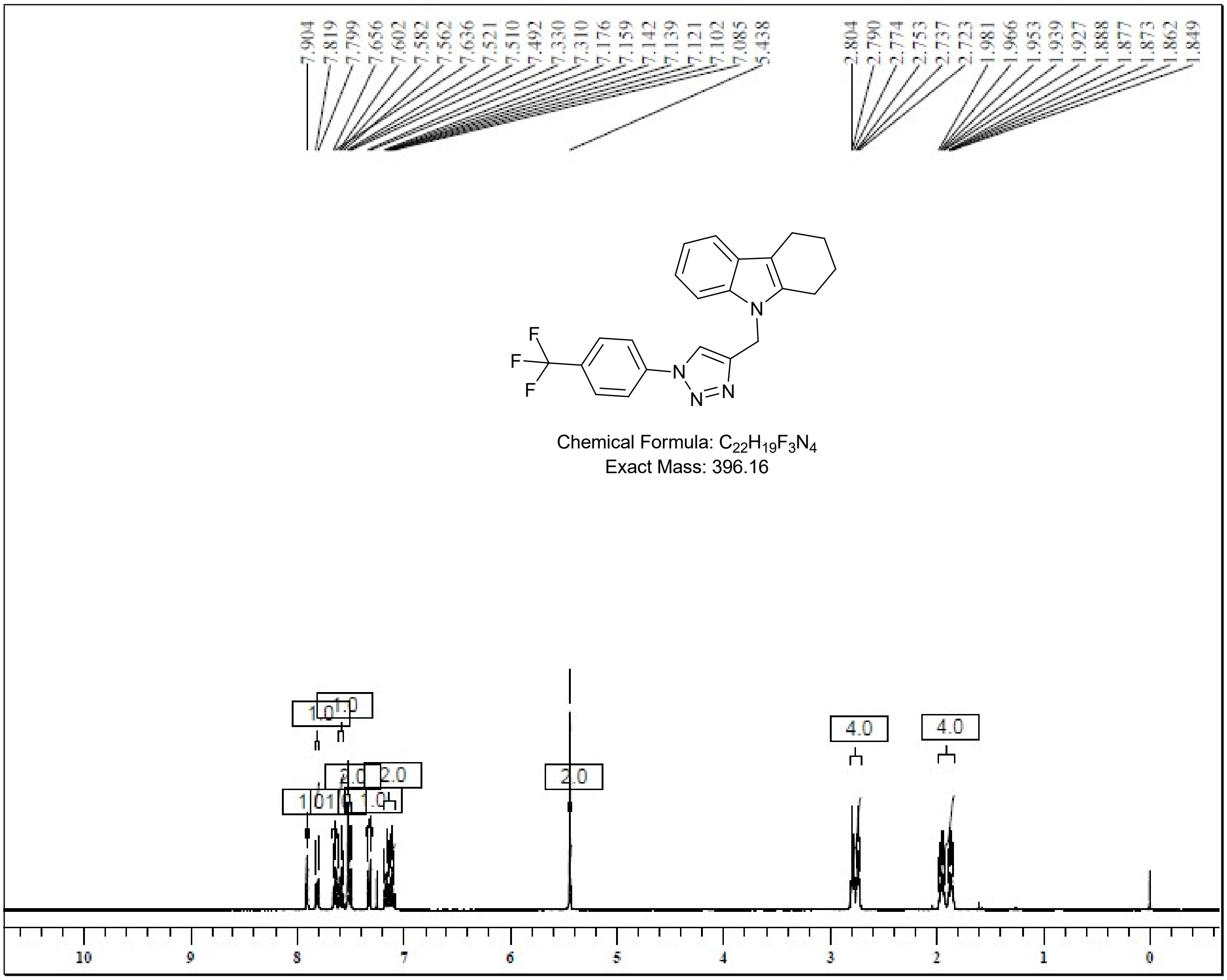

Figure S56: ^{1}H NMR (400 MHz, $CDCl_3$) of 9-((1-(4-(trifluoromethyl)phenyl)-1*H*-1,2,3-triazol-4-yl)methyl)-2,3,4,9-tetrahydro-1*H*-carbazole (**4n**)

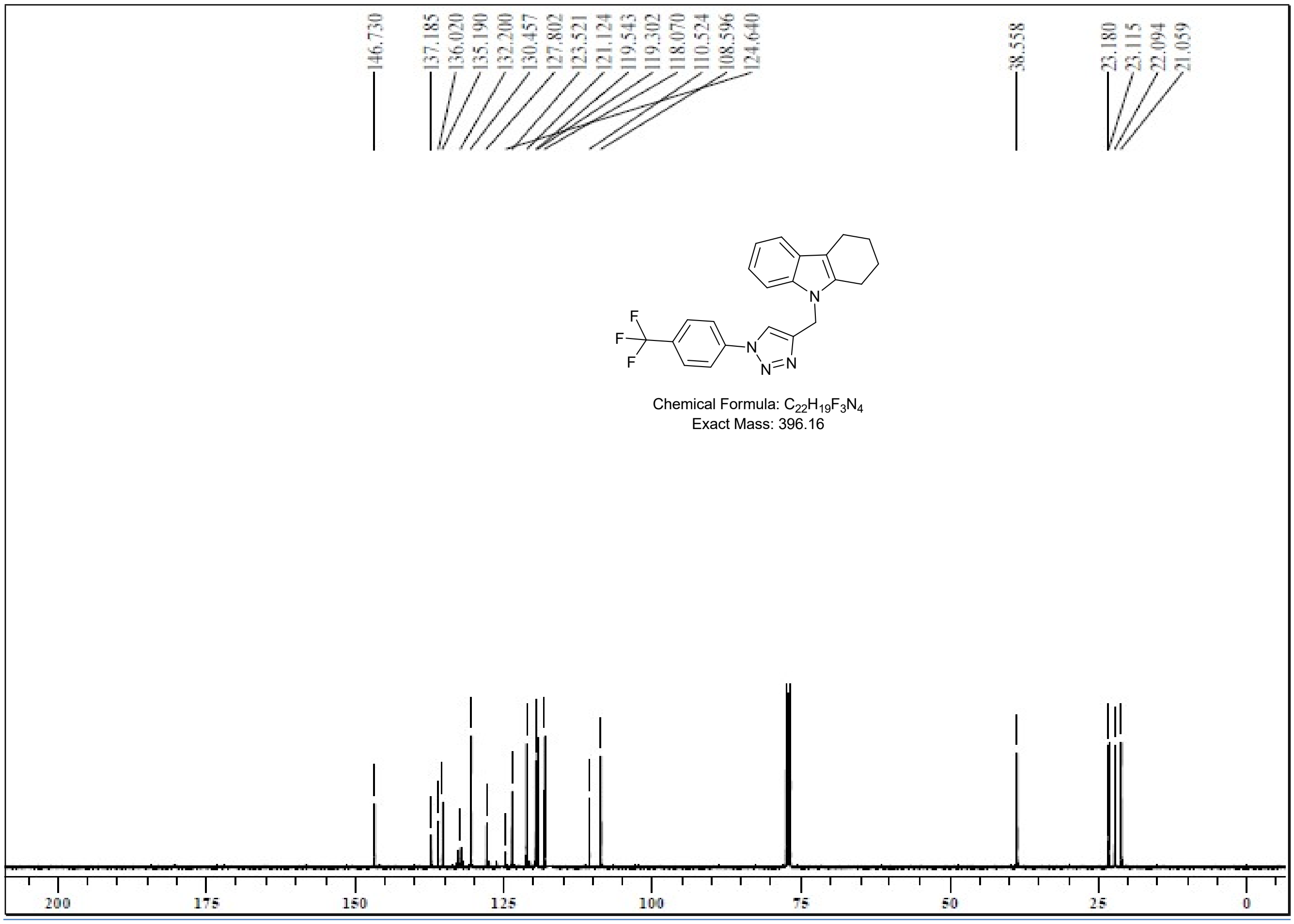

Figure S57: ^{13}C NMR (100 MHz, $CDCl_3$) spectrum of 9-((1-(4-(trifluoromethyl)phenyl)-1*H*-1,2,3-triazol-4-yl)methyl)-2,3,4,9-tetrahydro-1*H*-carbazole (**4n**)

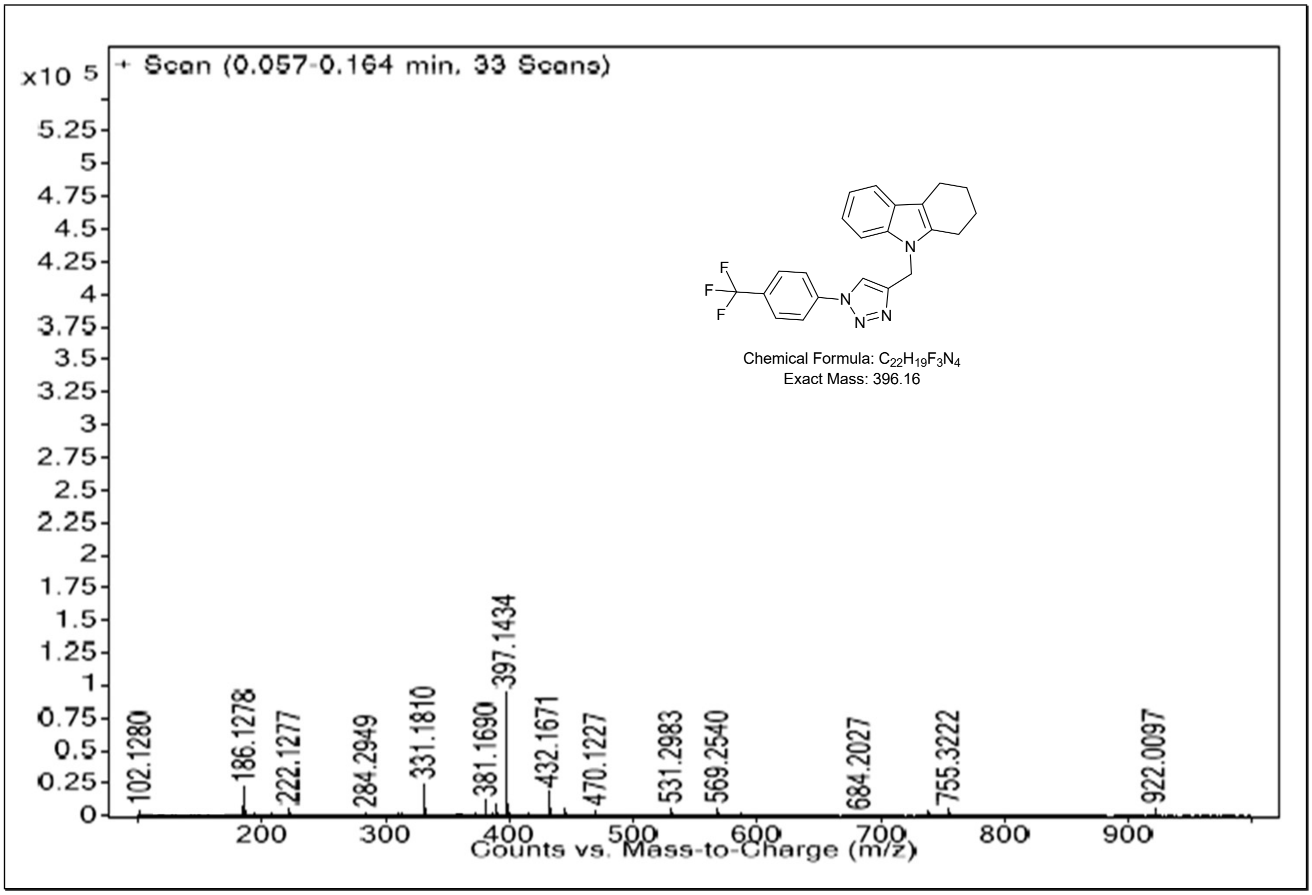

Figure S58: Mass (ES^+) spectrum of 9-((1-(4-(trifluoromethyl)phenyl)-1*H*-1,2,3-triazol-4-yl)methyl)-2,3,4,9-tetrahydro-1*H*-carbazole (**4n**)

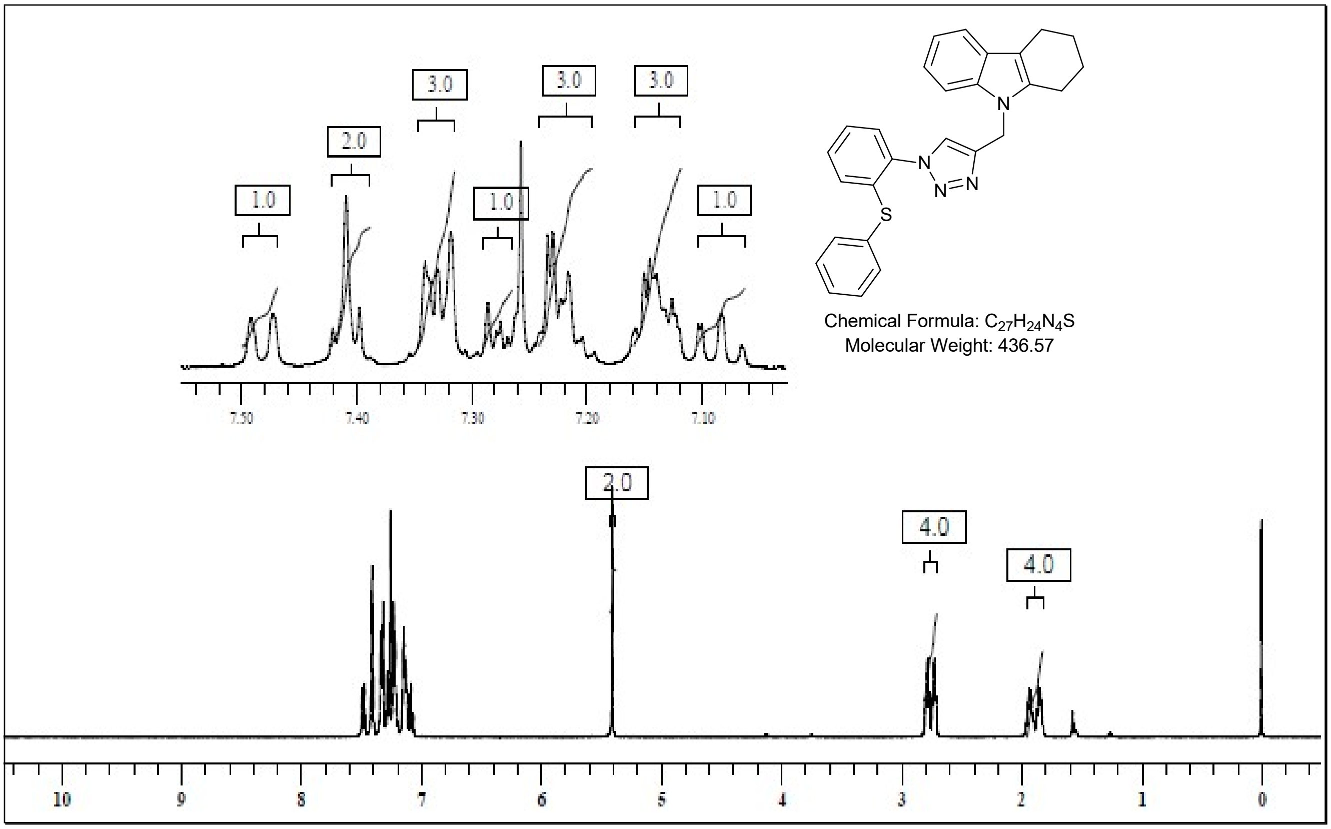

Figure S59: ^{1}H NMR (400 MHz, $CDCl_3$) of 9-((1-(2-(phenylthio)phenyl)-1*H*-1,2,3-triazol-4-yl)methyl)-2,3,4,9-tetrahydro-1*H*-carbazole (**4o**)

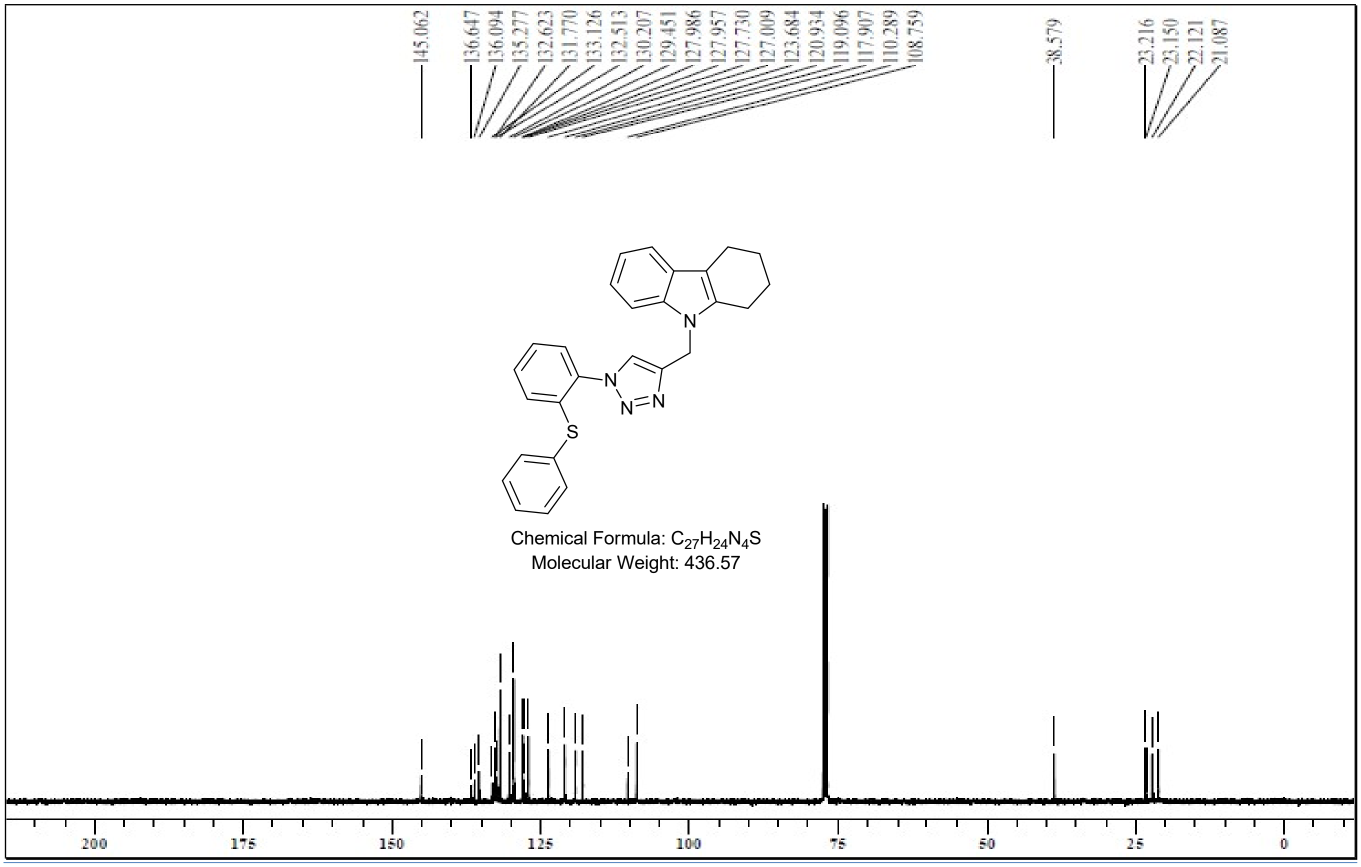

Figure S60: ^{13}C NMR (100 MHz, $CDCl_3$) spectrum of 9-((1-(2-(phenylthio)phenyl)-1*H*-1,2,3-triazol-4-yl)methyl)-2,3,4,9-tetrahydro-1*H*-carbazole (**4o**)

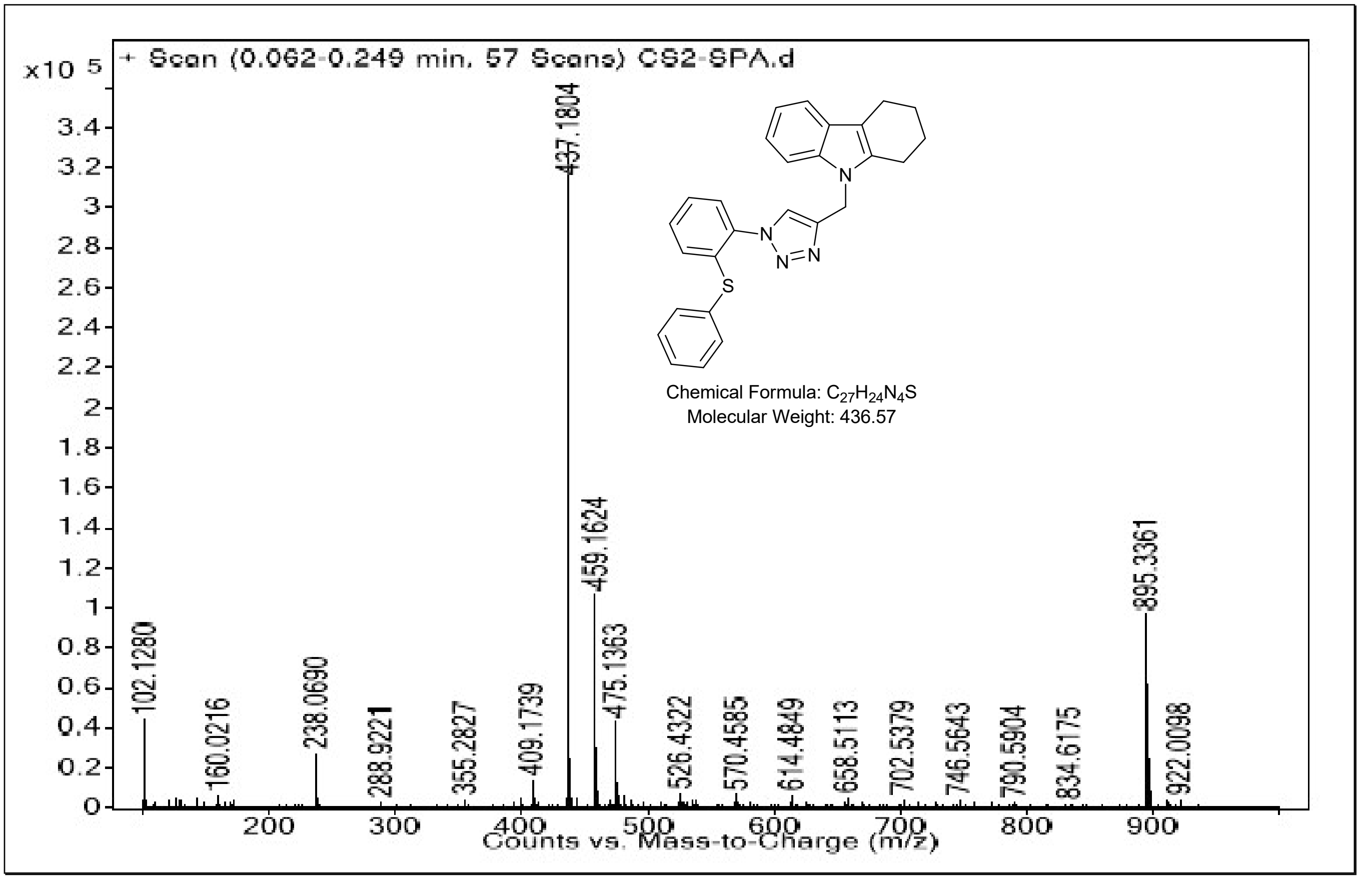

Figure S61: Mass (ES^+) spectrum of 9-((1-(2-(phenylthio)phenyl)-1*H*-1,2,3-triazol-4-yl)methyl)-2,3,4,9-tetrahydro-1*H*-carbazole (**4o**)

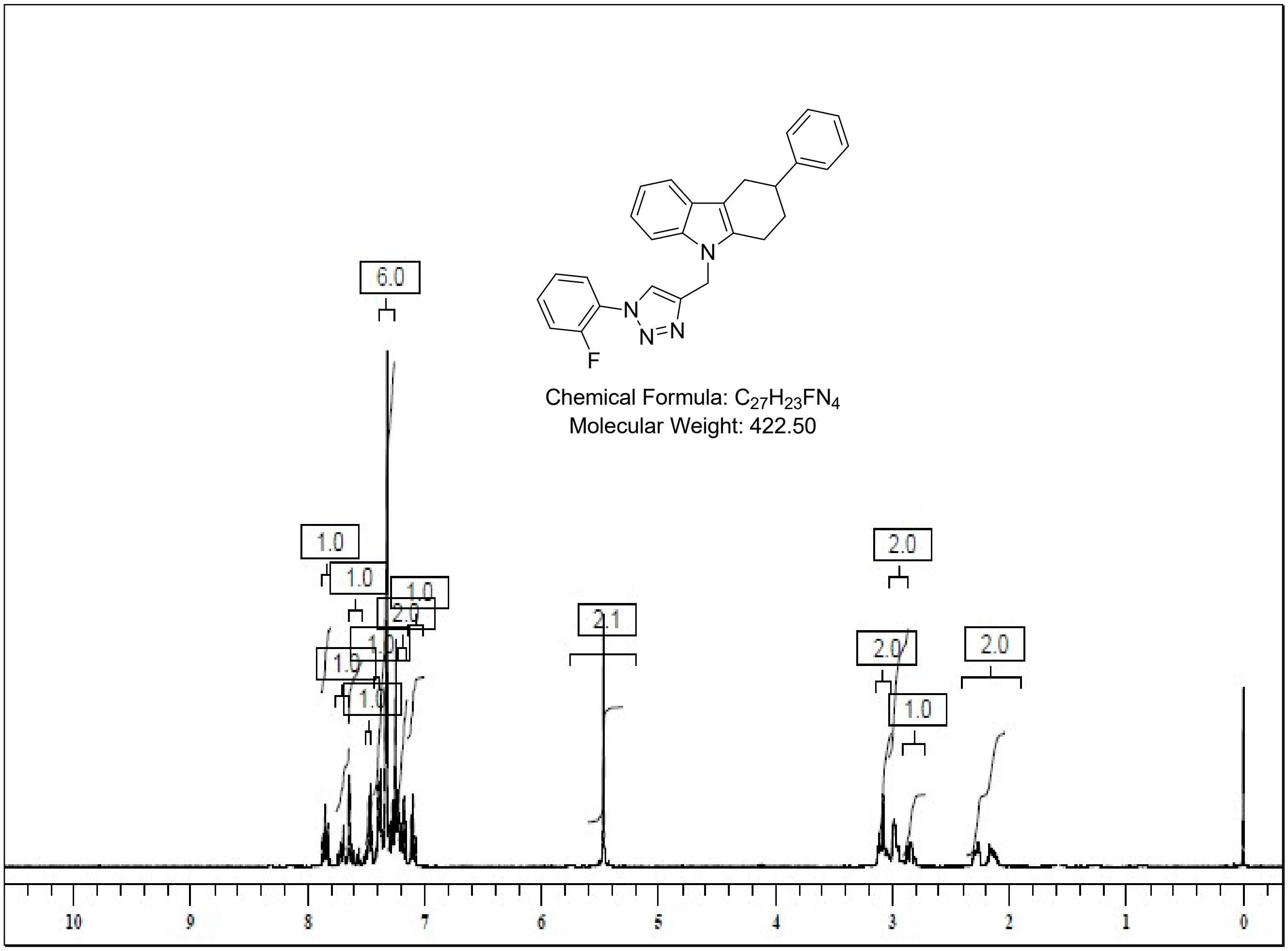

Figure S62: ^{1}H NMR (400 MHz, $CDCl_3$) of 9-((1-(2-fluorophenyl)-1*H*-1,2,3-triazol-4-yl)methyl)-3-phenyl-2,3,4,9-tetrahydro-1*H*-carbazole (**4p**)

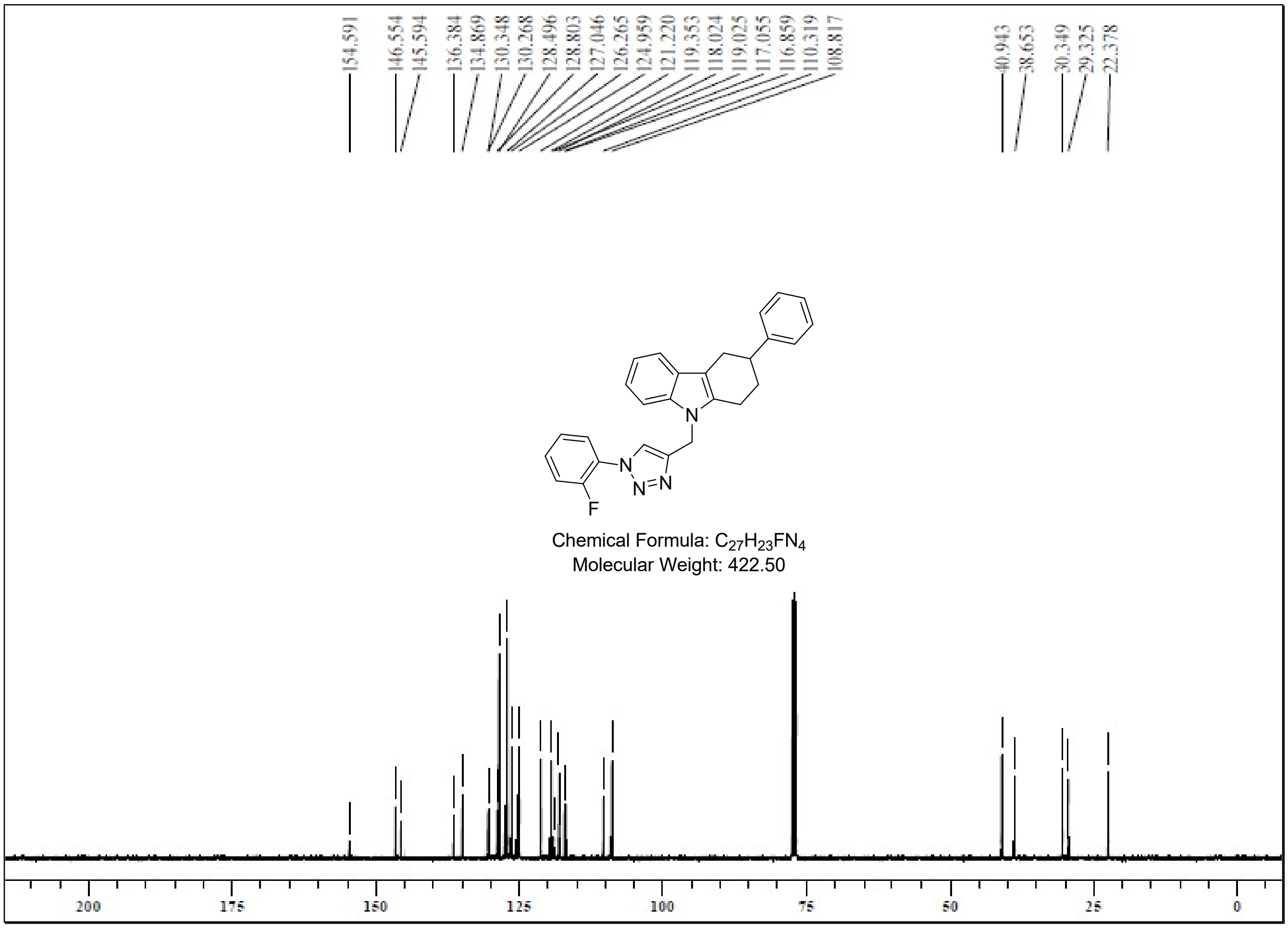

Figure S63: ^{13}C NMR (100 MHz, $CDCl_3$) spectrum of 9-((1-(2-fluorophenyl)-1*H*-1,2,3-triazol-4-yl)methyl)-3-phenyl-2,3,4,9-tetrahydro-1*H*-carbazole (**4p**)

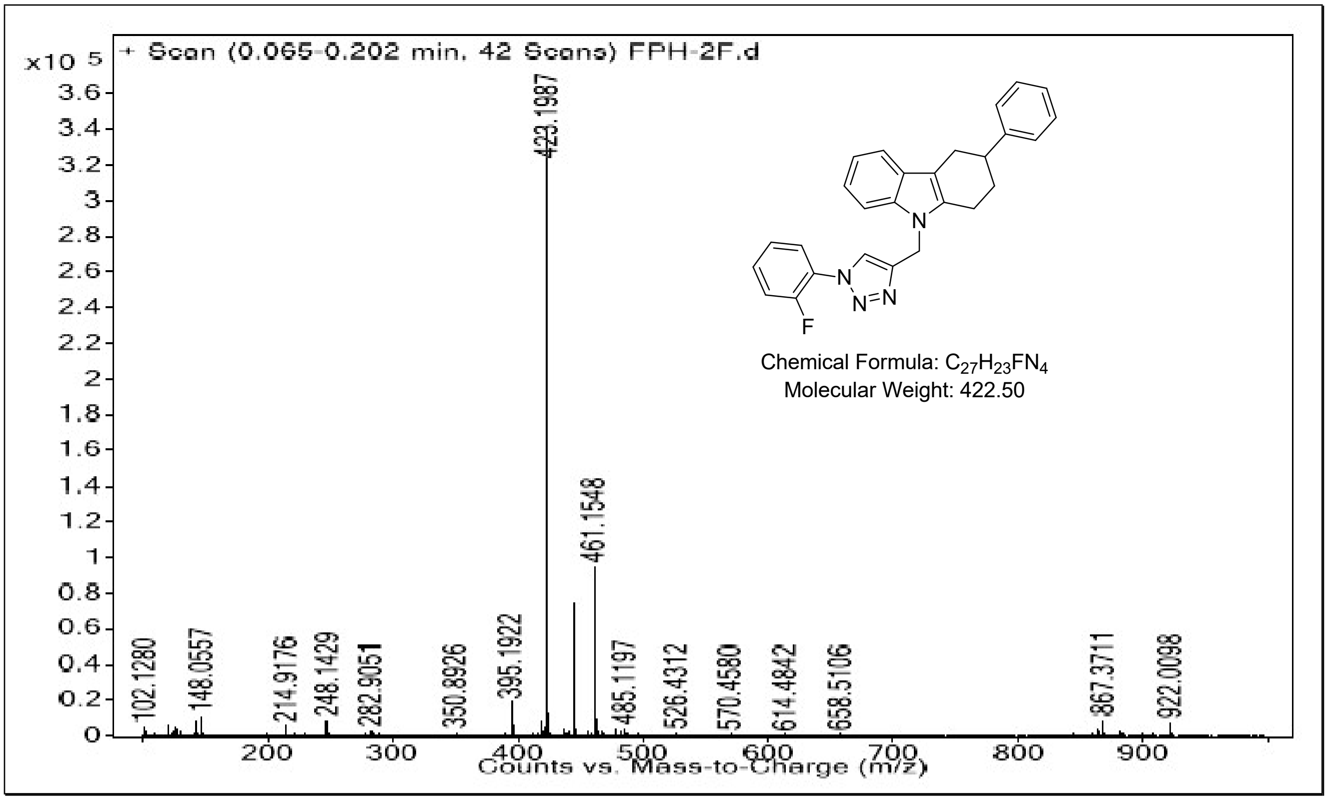

Figure S64: Mass (ES$^+$) spectrum of 9-((1-(2-fluorophenyl)-1*H*-1,2,3-triazol-4-yl)methyl)-3-phenyl-2,3,4,9-tetrahydro-1*H*-carbazole (**4p**)

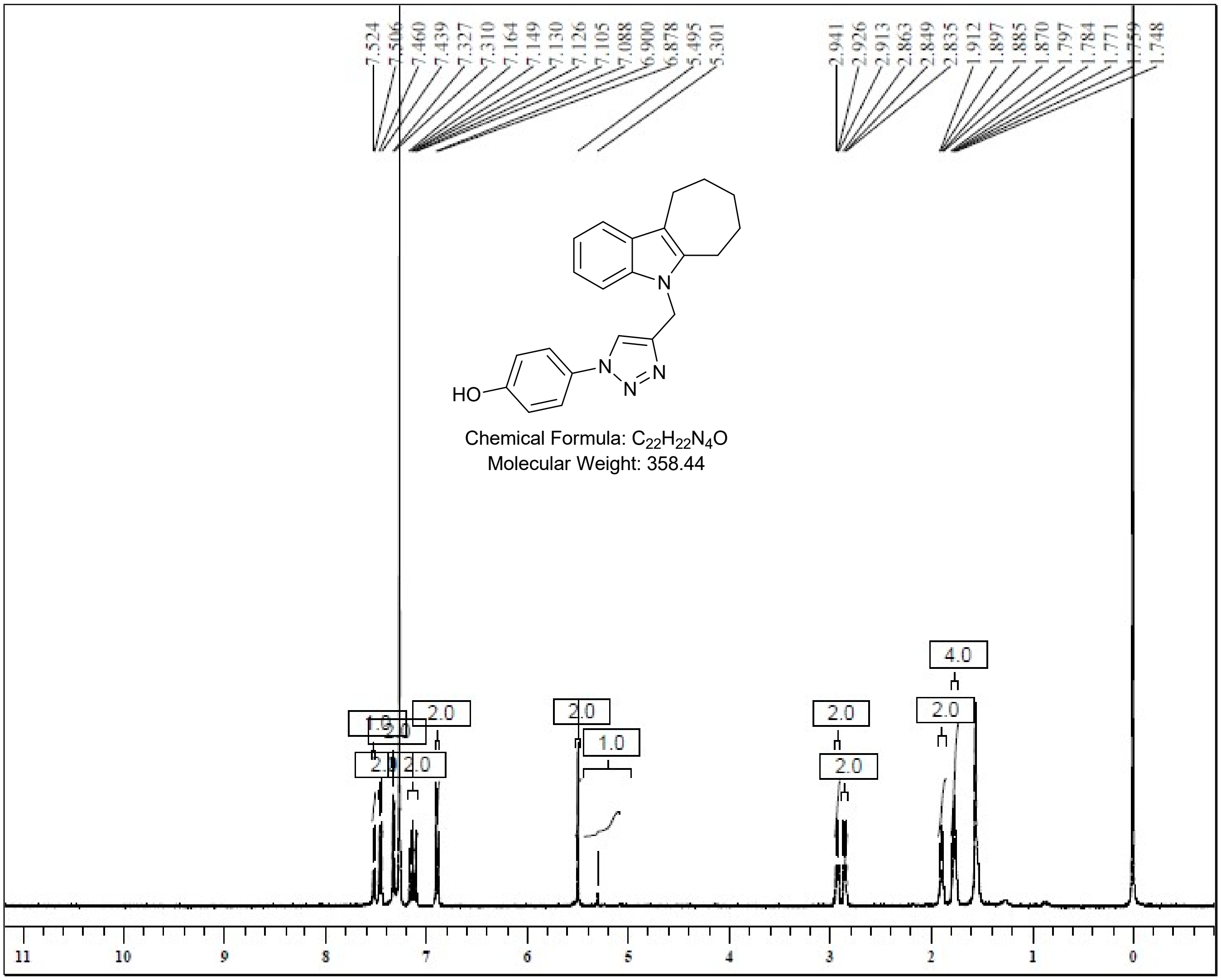

Figure S65: ^{1}H NMR (400 MHz, $CDCl_3$) of 4-(4-((7,8,9,10-tetrahydrocyclohepta[b]indol-5(6*H*)-yl)methyl)-1*H*-1,2,3-triazol-1-yl)phenol (**4q**)

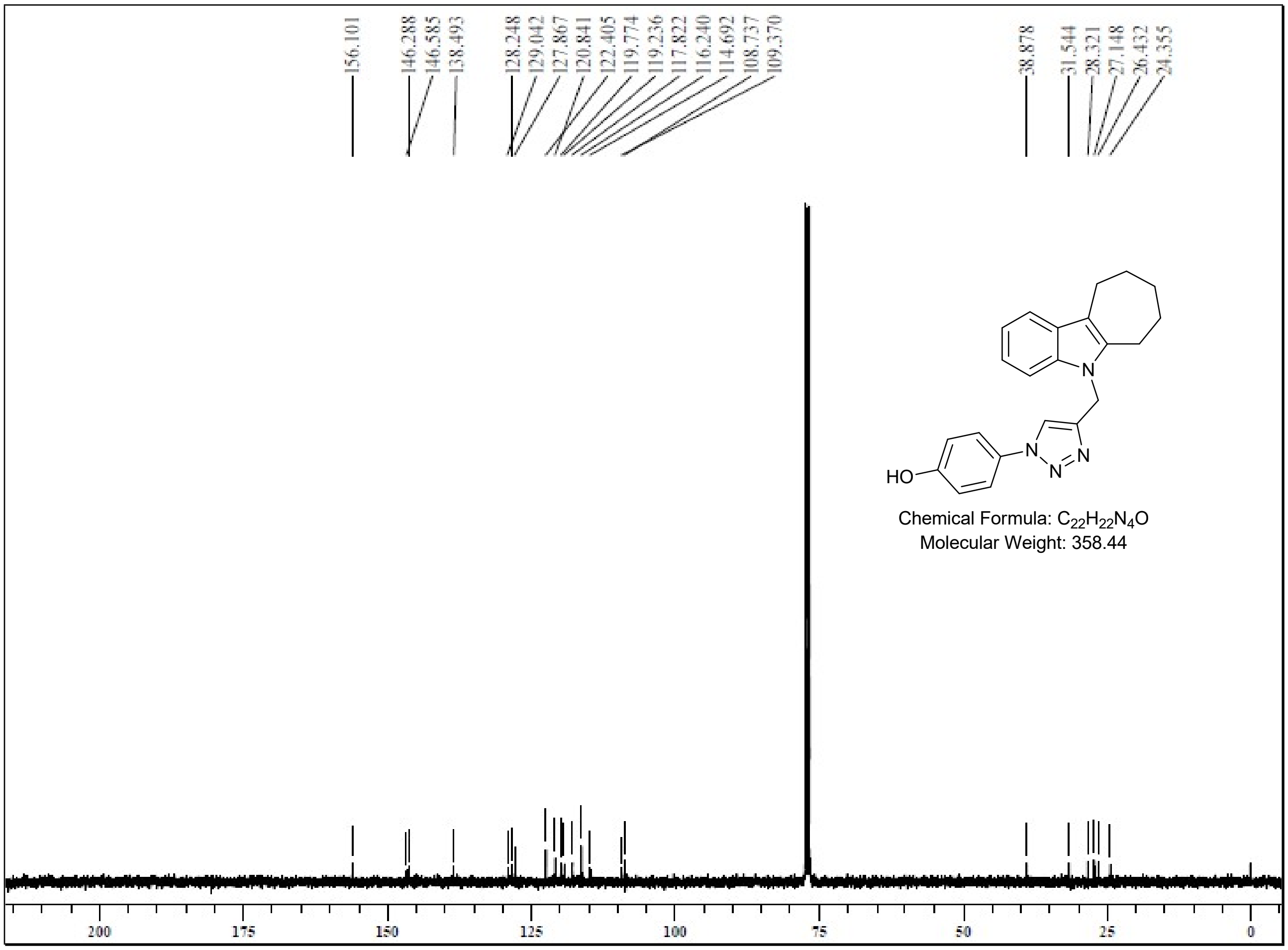

Figure S66: ^{13}C NMR (100 MHz, $CDCl_3$) spectrum of 4-(4-((7,8,9,10-tetrahydrocyclohepta[b]indol-5(6*H*)-yl)methyl)-1*H*-1,2,3-triazol-1-yl)phenol (**4q**)

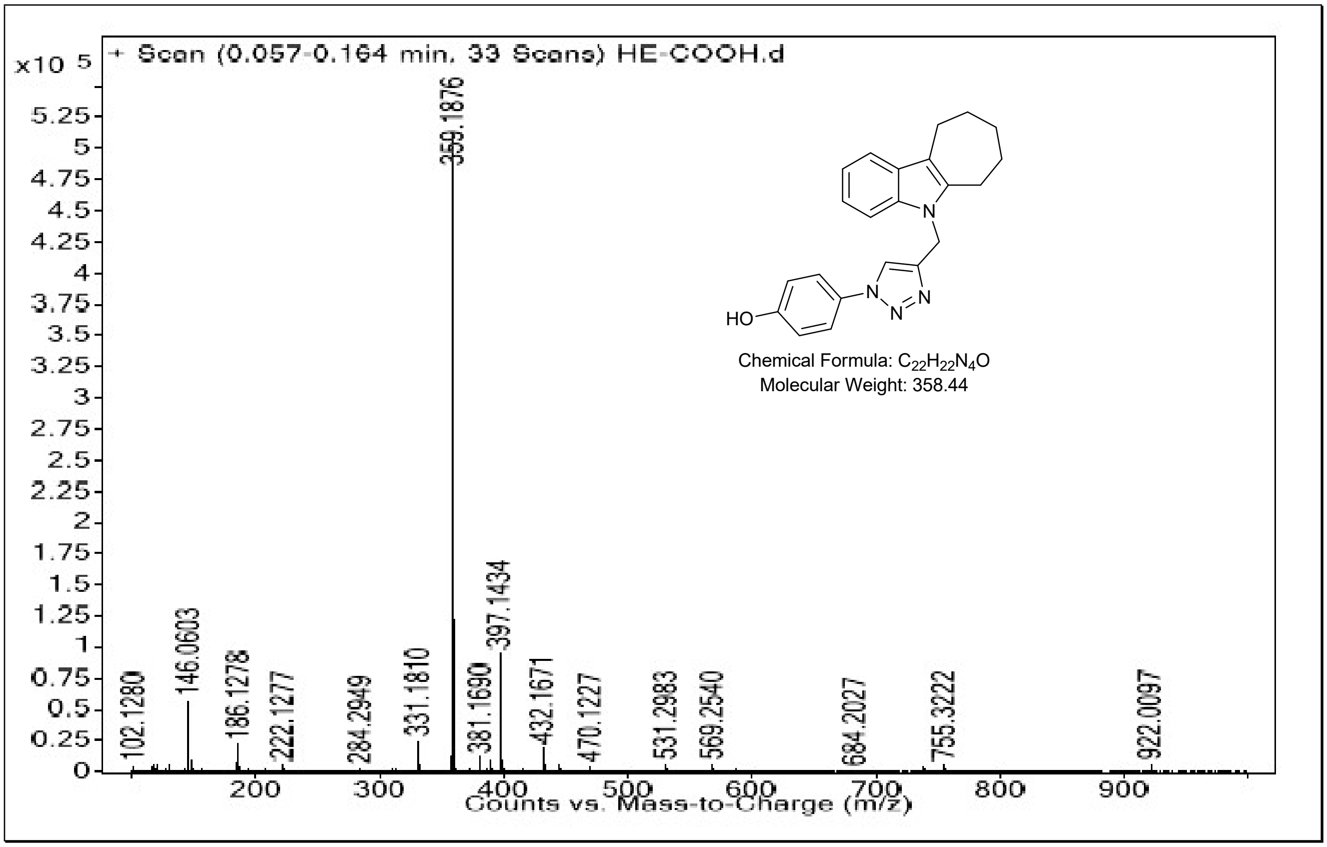

Figure S67: Mass (ES+) spectrum of 4-(4-((7,8,9,10-tetrahydrocyclohepta[b]indol-5(6*H*)-yl)methyl)-1*H*-1,2,3-triazol-1-yl)phenol (**4q**)

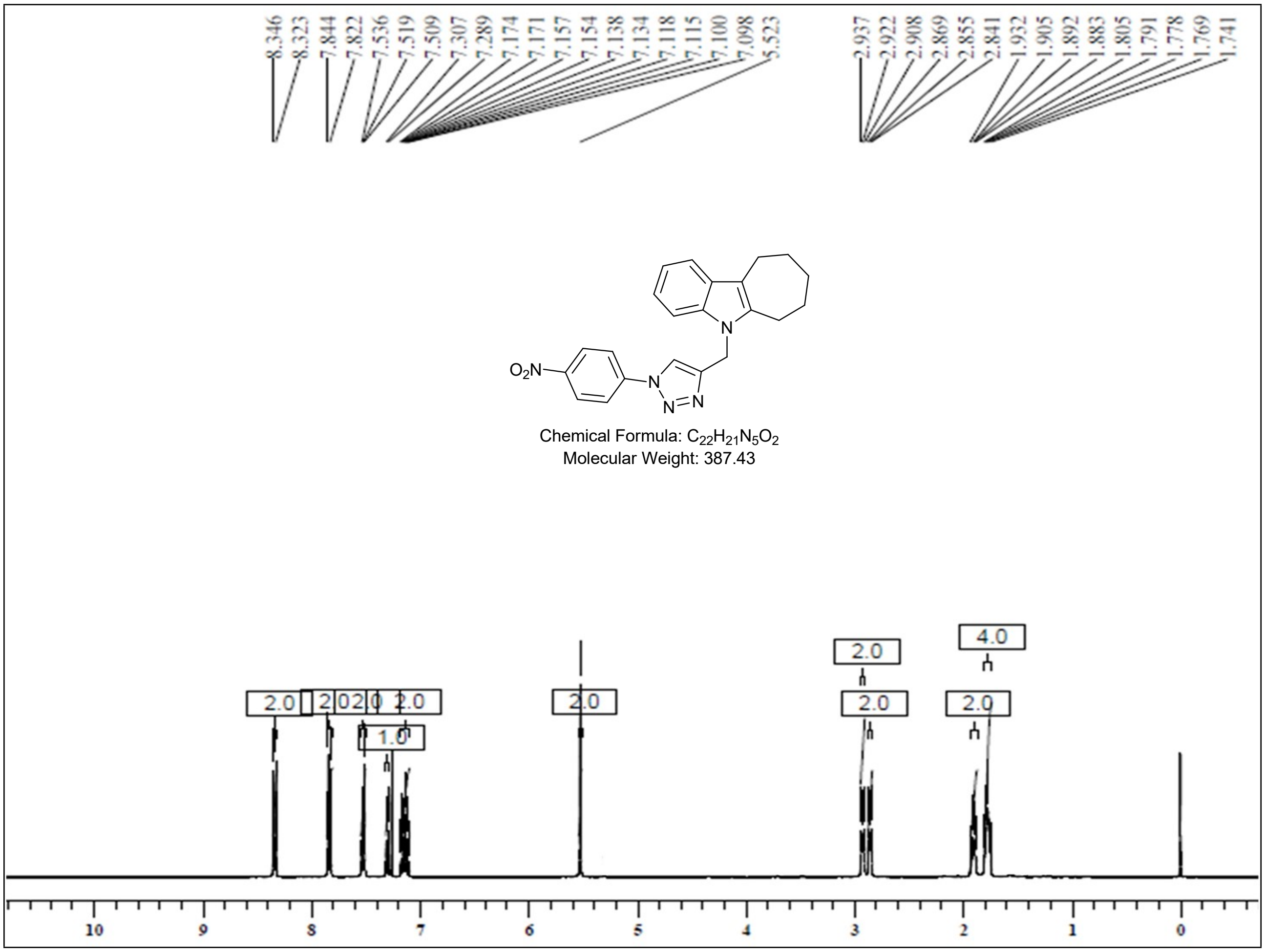

Figure S68: ^{1}H NMR (400 MHz, $CDCl_3$) of 5-((1-(4-nitrophenyl)-1*H*-1,2,3-triazol-4-yl)methyl)-5,6,7,8,9,10 hexahydrocyclohepta[b]indole (**4r**)

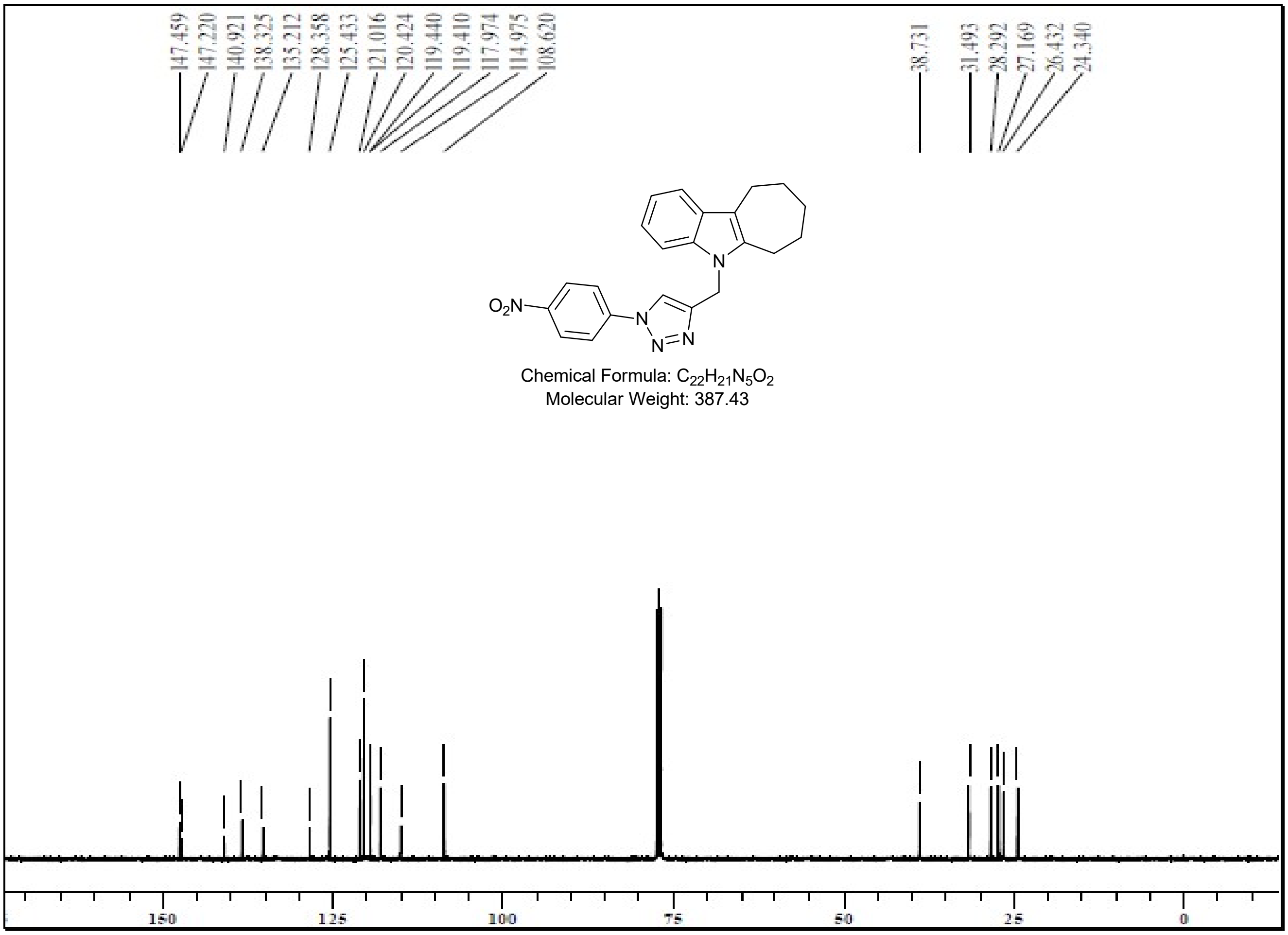

Figure S69: ^{13}C NMR (100 MHz, $CDCl_3$) spectrum of 5-((1-(4-nitrophenyl)-1*H*-1,2,3-triazol-4-yl)methyl)-5,6,7,8,9,10-hexahydrocyclohepta[b]indole (**4r**)

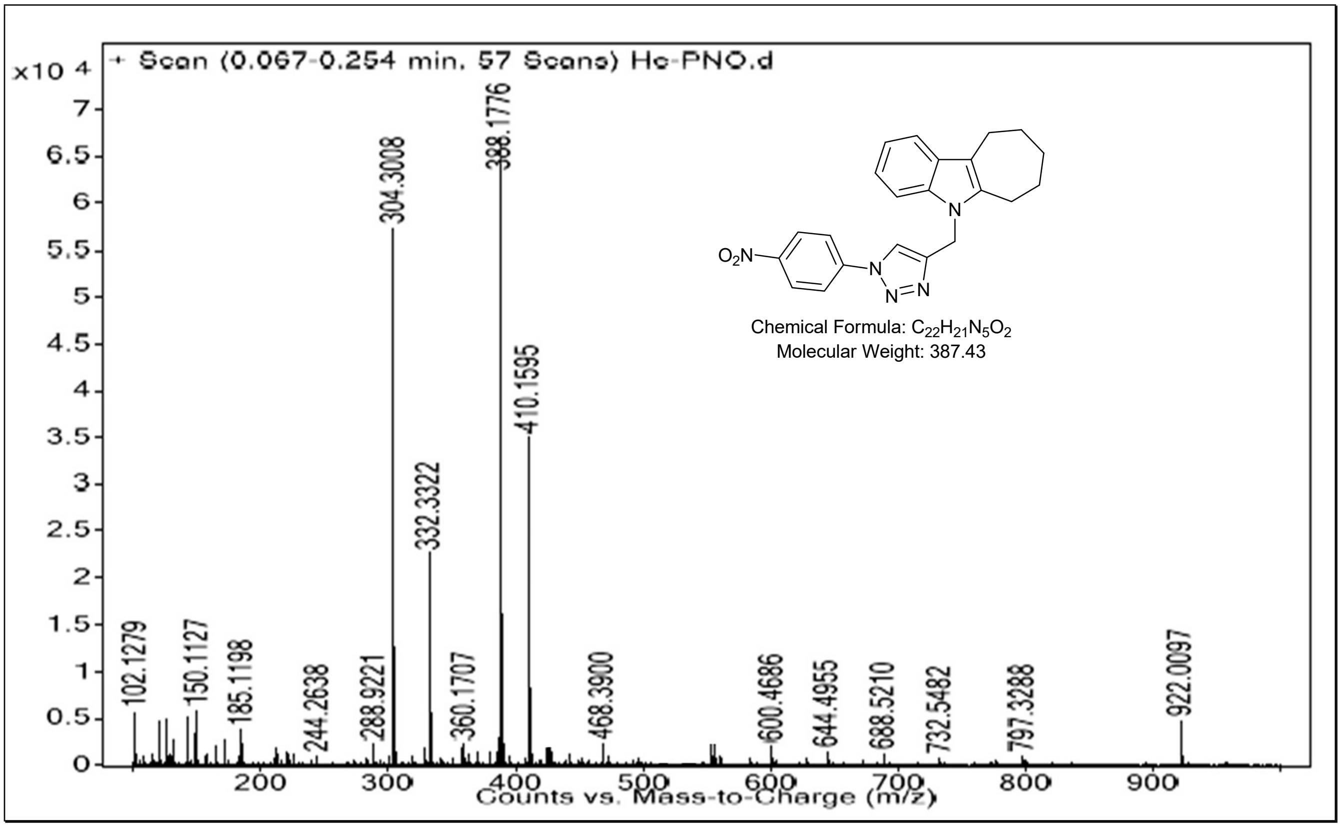

Figure S70: Mass (ES^+) spectrum of 5-((1-(4-nitrophenyl)-1*H*-1,2,3-triazol-4-yl)methyl)-5,6,7,8,9,10-hexahydrocyclohepta[b]indole (**4r**)

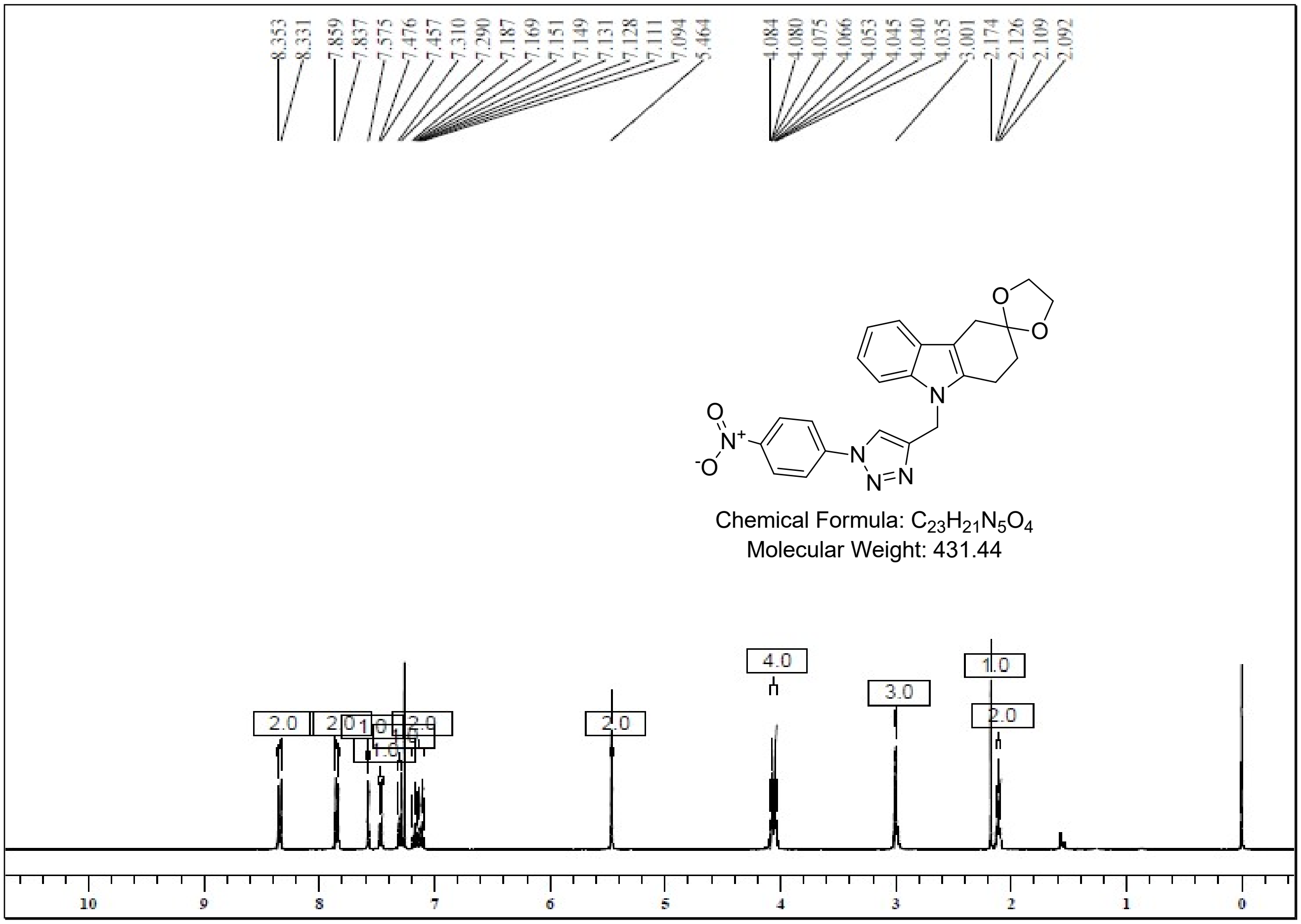

Figure S71: ^{1}H NMR (400 MHz, $CDCl_3$) of 9-((1-(4-nitrophenyl)-1*H*-1,2,3-triazol-4-yl)methyl)-1,2,4,9-tetrahydrospiro[carbazole-3,2'-[1,3]dioxolane] (**4s**)

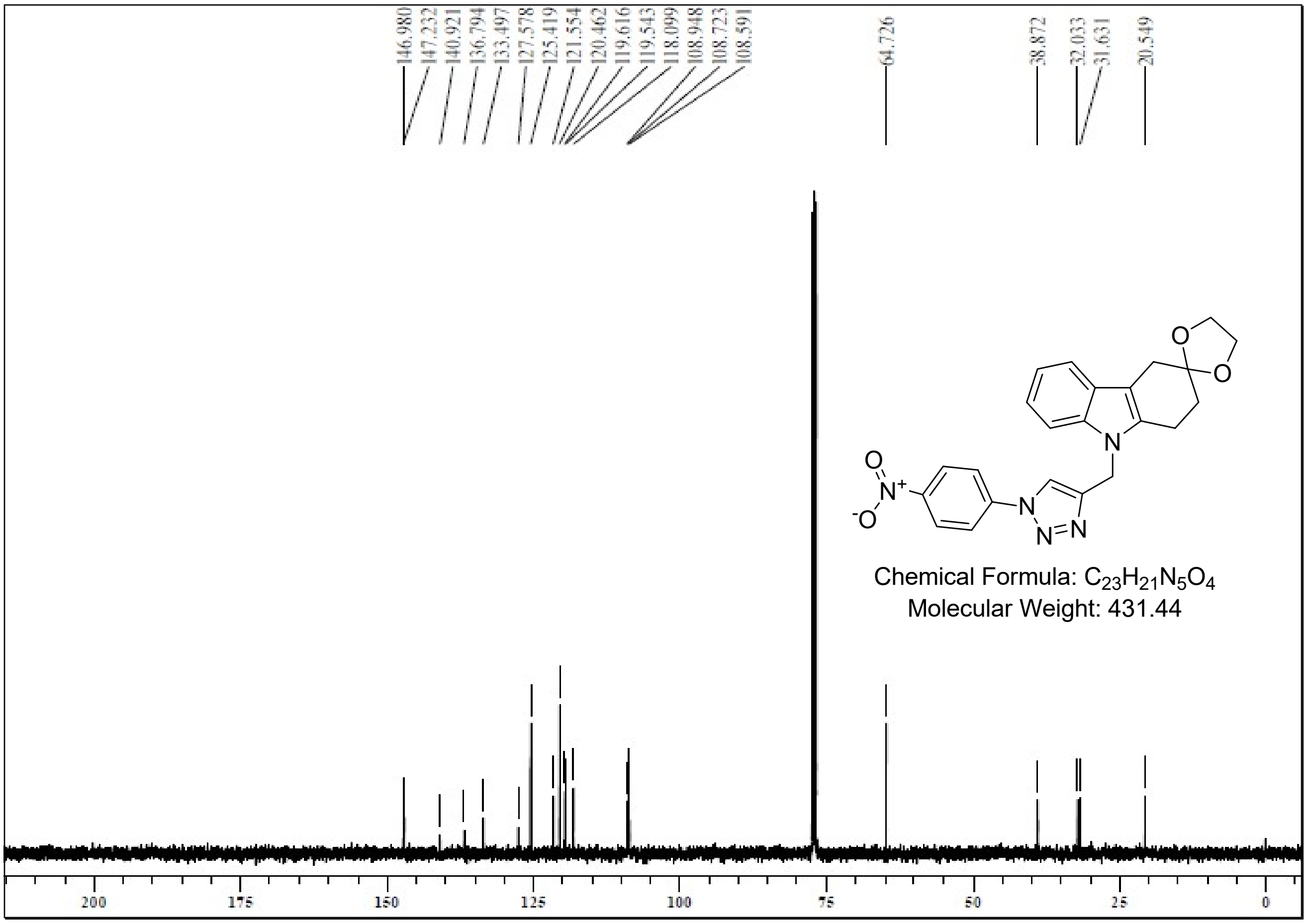

Figure S72: ^{13}C NMR (100 MHz, $CDCl_3$) spectrum of 9-((1-(4-nitrophenyl)-1*H*-1,2,3-triazol-4-yl)methyl)-1,2,4,9-tetrahydrospiro[carbazole-3,2'-[1,3]dioxolane] (**4s**)

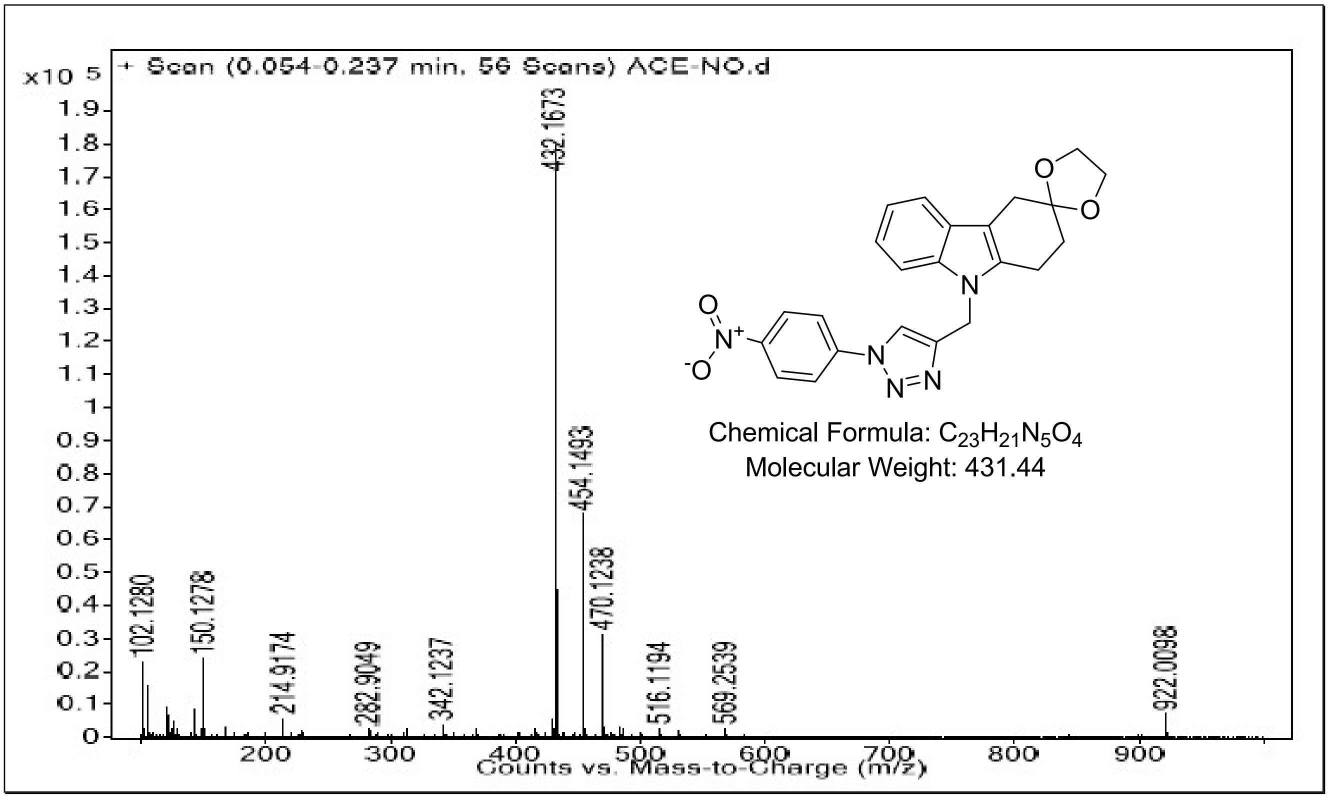

Figure S73: Mass (ES^+) spectrum of 9-((1-(4-nitrophenyl)-1*H*-1,2,3-triazol-4-yl)methyl)-1,2,4,9-tetrahydrospiro[carbazole-3,2'-[1,3]dioxolane] (**4s**)

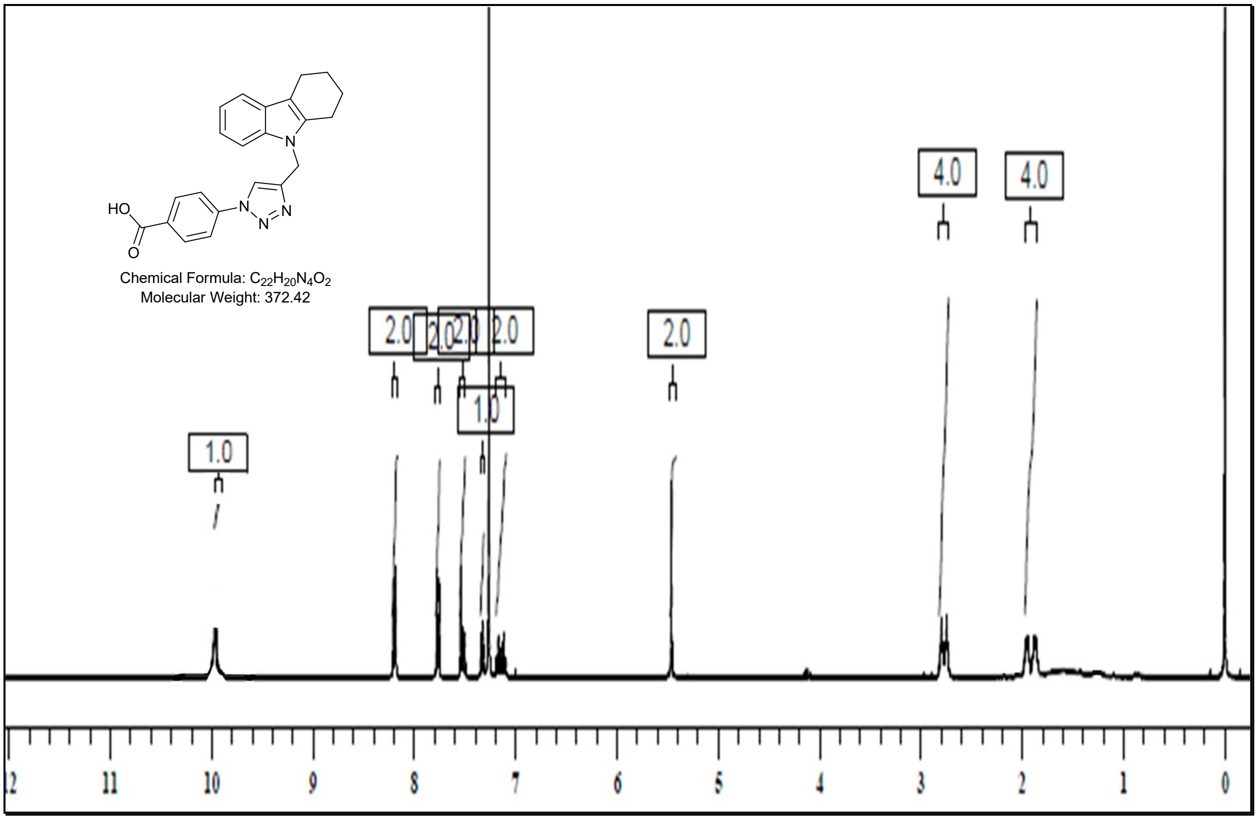

Figure S74: ^{1}H NMR (400 MHz, $CDCl_3$) of 4-(4-((3,4-dihydro-1*H*-carbazol-9(2*H*)-yl)methyl)-1*H*-1,2,3-triazol-1-yl)benzoic acid (**4t**)

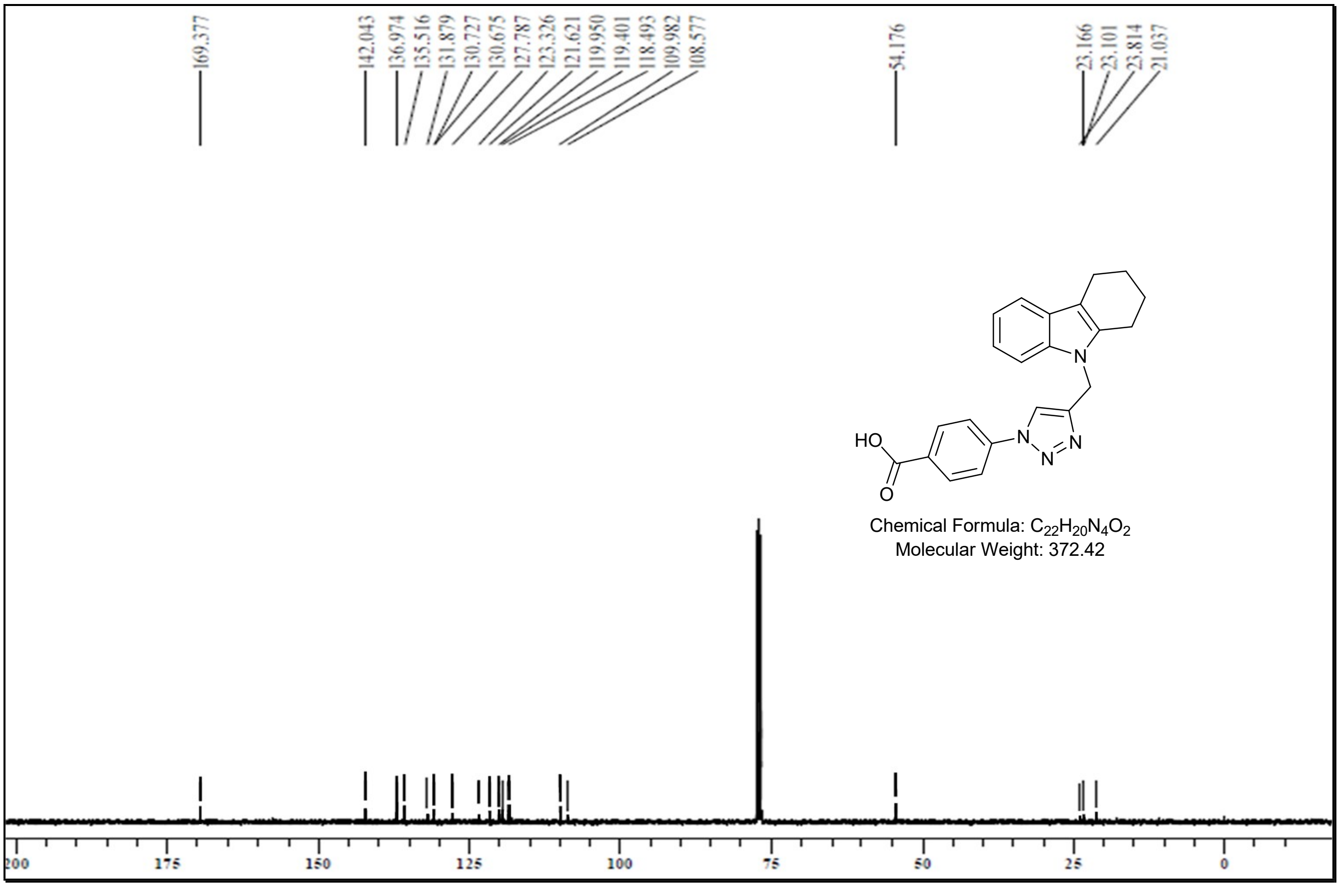

Figure S75: ^{13}C NMR (100 MHz, $CDCl_3$) spectrum of 4-(4-((3,4-dihydro-1*H*-carbazol-9(2*H*)-yl)methyl)-1*H*-1,2,3-triazol-1-yl)benzoic acid (**4t**)

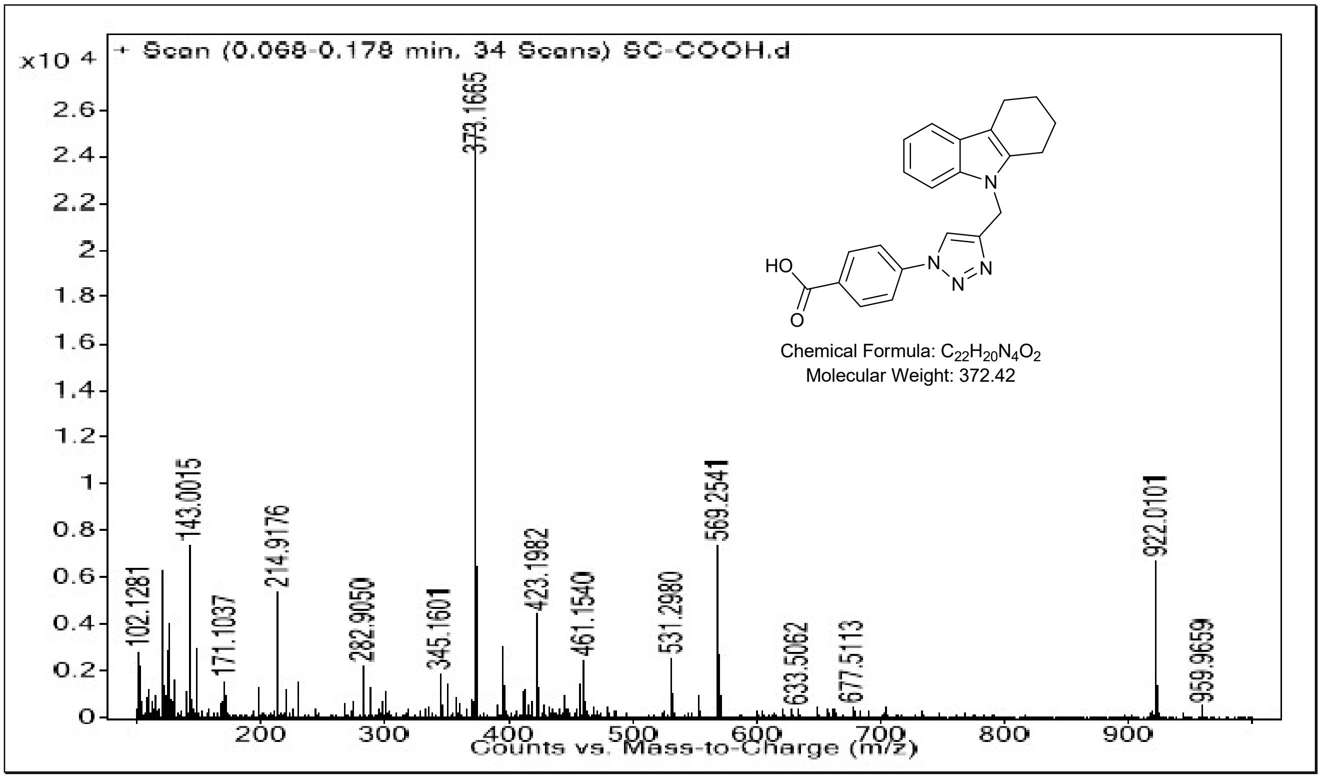

Figure S76: Mass (ES^+) spectrum of 4-(4-((3,4-dihydro-1*H*-carbazol-9(2*H*)-yl)methyl)-1*H*-1,2,3-triazol-1-yl)benzoic acid (**4t**)

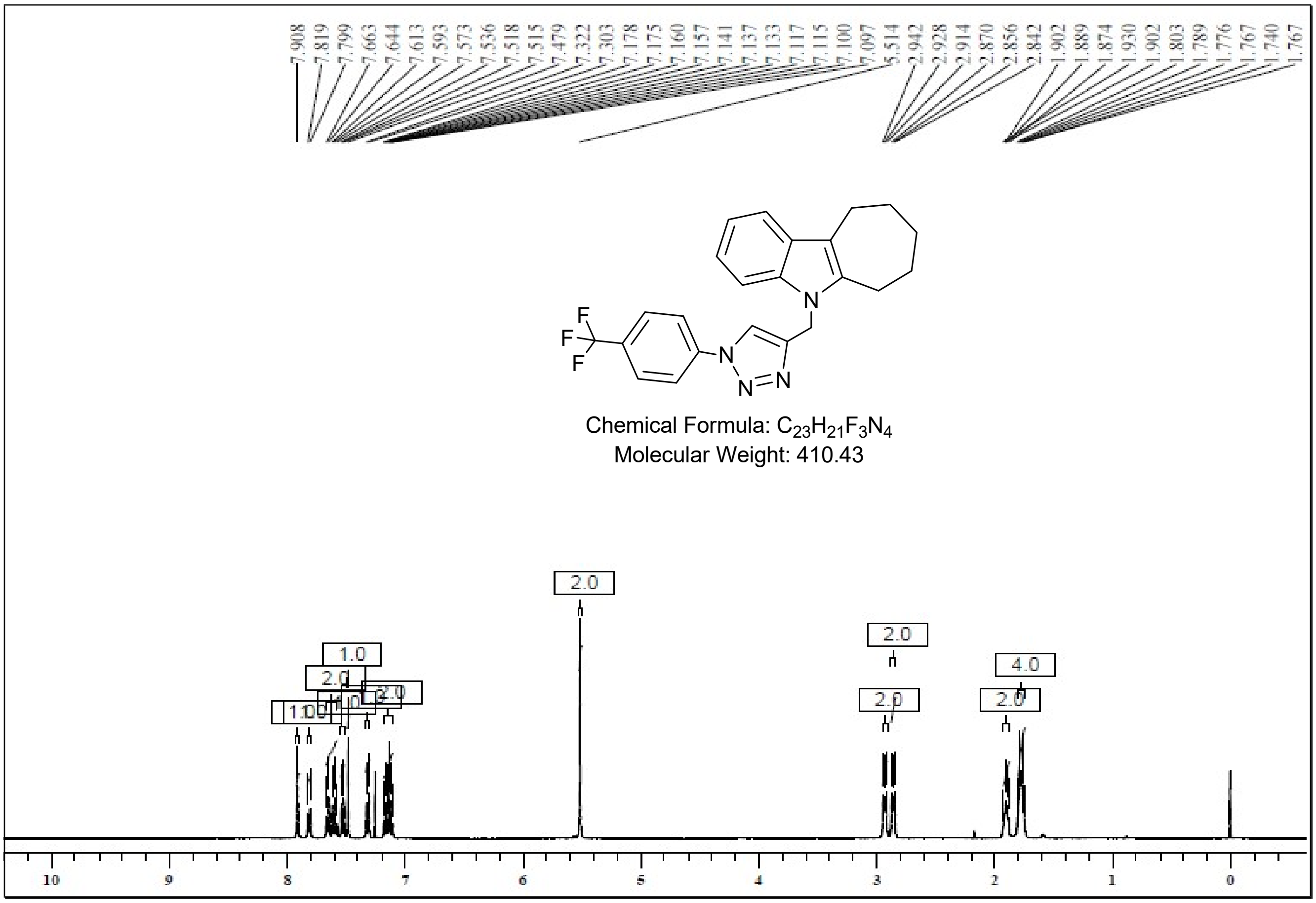

Figure S77: ^{1}H NMR (400 MHz, $CDCl_3$) of 5-((1-(4-(trifluoromethyl)phenyl)-1*H*-1,2,3-triazol-4-yl)methyl)-5,6,7,8,9,10-hexahydrocyclohepta[b]indole (**4u**)

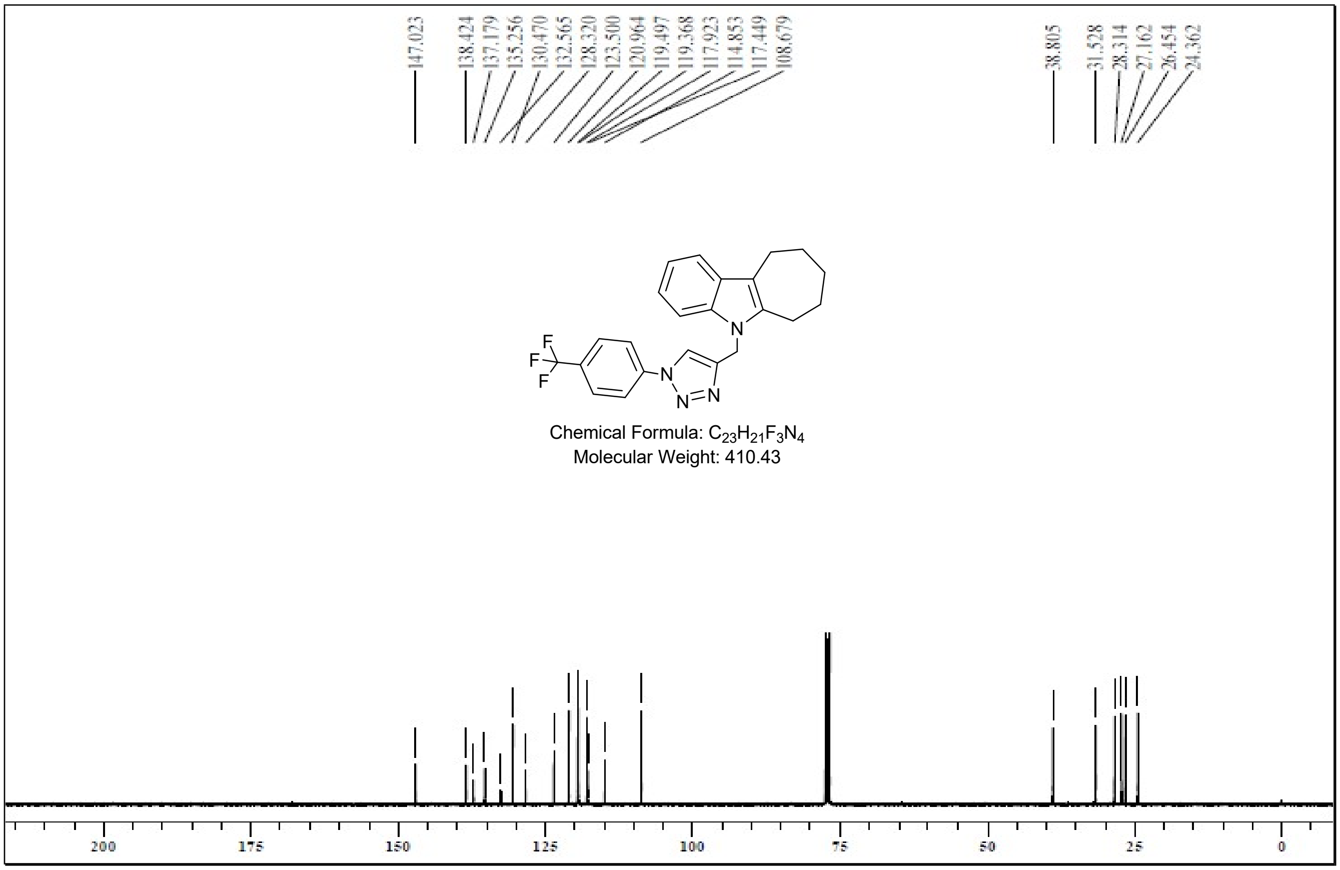

Figure S78: ^{13}C NMR (100 MHz, $CDCl_3$) spectrum of 5-((1-(4-(trifluoromethyl)phenyl)-1*H*-1,2,3-triazol-4-yl)methyl)-5,6,7,8,9,10-hexahydrocyclohepta[b]indole (**4u**)

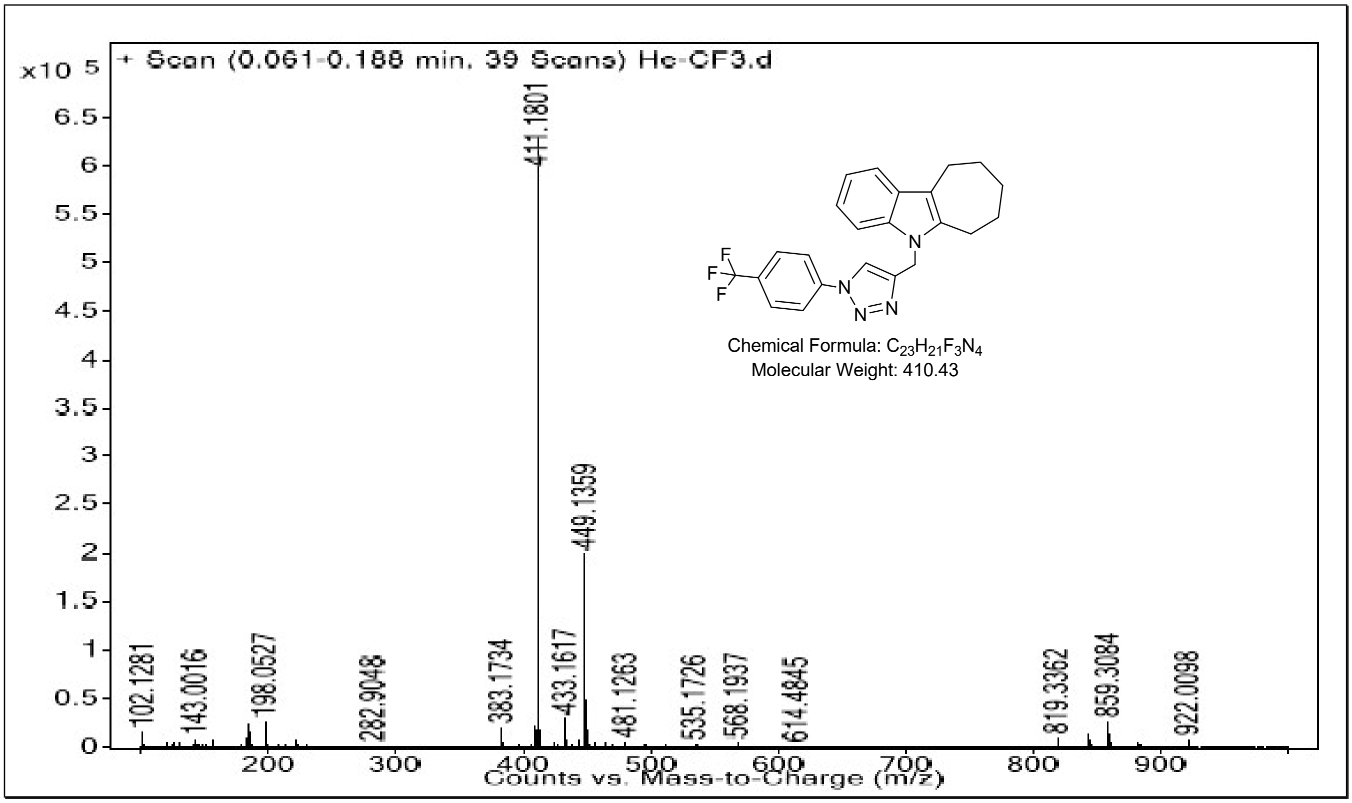

Figure S79: Mass (ES^+) spectrum of 5-((1-(4-(trifluoromethyl)phenyl)-1*H*-1,2,3-triazol-4-yl)methyl)-5,6,7,8,9,10-hexahydrocyclohepta[b]indole (**4u**)
